# Design, Synthesis, Anticancer Evaluation and Molecular Docking of Pyrimidine, Pyrido[4,3-d]pyrimidine and 5,6,7,8-Tetrahydropyrido[3,4-d]pyrimidine Derivatives as Novel KRAS-G12D Inhibitors and PROTACs

**DOI:** 10.3390/ph18050696

**Published:** 2025-05-08

**Authors:** Hailong Yang, Lu Gan, Huabei Zhang

**Affiliations:** Key Laboratory of Radiopharmaceuticals of the Ministry of Education, College of Chemistry, Beijing Normal University, Beijing 100875, China; huaxuehailong@163.com (H.Y.); 202221150066@mail.bnu.edu.cn (L.G.)

**Keywords:** KRAS-G12D, structure-based drug design, inhibitors, molecular docking

## Abstract

**Background**: KRAS-G12D mutations drive 20–50% of pancreatic/biliary cancers yet remain challenging to target due to GTP-pocket conservation and high cellular GTP levels. While allosteric inhibitors targeting the SWII pocket (e.g., MRTX1133) show promise, limited chemical diversity and paradoxical cellular/enzymatic activity relationships necessitate the exploration of novel scaffolds. This study aims to develop KRAS-G12D inhibitors and PROTACs to offer a selection of new chemical entities through systematic structure–activity optimization and evaluate their therapeutic potential through PROTAC derivatization. **Methods**: Eleven compounds featuring heterocyclic cores (pyrimidine/pyrido[4,3-d]pyrimidine/5,6,7,8-tetrahydroprodo[3,4-d]pyrimidine) were designed via structure-based drug design. Antiproliferative activity against KRAS-G12D (Panc1), KRAS-G13D (HCT116) and wild-type (A549) cells was assessed using the CCK-8 assay. KRAS-G12D enzymatic inhibition was measured using a GTPase activity assay. Molecular docking simulations (Sybyl 2.0; PDB:7RPZ) elucidated binding modes. Two PROTACs were synthesized from lead compounds by conjugating E3 ligase linkers. All the novel inhibitors and PROTACs were characterized by means of NMR or HRMS. **Results**: Compound **10c** demonstrated selective anti-proliferation in Panc1 cells (IC_50_ = 1.40 μM) with 4.9-fold greater selectivity over wild-type cells, despite weak enzymatic inhibition (IC_50_ > 10 μM). Docking revealed critical hydrogen bonds between its protonated 3,8-diazabicyclo[3.2.1]octane moiety and Asp12/Gly60. The enzymatic inhibitor **10k** showed potent KRAS-G12D inhibition (IC_50_ = 0.009 μM) through homopiperazine-mediated interactions with Glu92/His95. Derived PROTACs **26a**/**b** exhibited reduced potency (IC_50_ = 3–5 μM vs. parental **10k**: 2.22 μM), potentially due to impaired membrane permeability. **Conclusions**: Eleven novel KRAS-G12D inhibitors with a seven-membered ring pharmacophore were synthesized. Compound **10c** showed strong anti-proliferative activity, while **10k** exhibited potent enzymatic inhibition. Two PROTACs were designed but showed no clear advantage over **10k**. This study provides valuable insights for KRAS-targeted drug development.

## 1. Introduction

Mutations in Ras genes are associated with 30% of human malignancies. These mutations impair the ability of RAS proteins to switch from a GTP-bound ON state to a GDP-bound OFF state, and this GTP-bound ON state will continuously activate downstream signaling pathways, resulting in the constitutive activation of downstream signaling pathways and the formation of cancer. Codons 12, 13, 61 and 146 are the main mutation hotspots, and of these, codon 12 is the most frequently mutated site, as seen for G12D, G12V and G12C. Overall, 20–50% of KRAS-mutated cancers carry a G12D mutation, including 50% of ampullary carcinoma, 48% of appendiceal adenocarcinoma and 44% of cholangiocarcinoma [1,2]. Therefore, the KRAS-G12D protein is a promising target for drug development. However, it is difficult to target the GTP binding pocket directly due to its high conservation rate and the concentration of GTP in the cell being high [3]. Since the identification of the SWII pocket as the effective binding site for inhibitors in 2013 [4], drug development targeting mutated KRAS has accelerated significantly. Sotarasib [5] and adagrasib [6], two prominent KRAS-G12C inhibitors, were successively greenlit for the market by the FDA (Food and Drug Administration) in 2021 and 2022, respectively. The marvelous success of targeting cancers with KRAS-G12C mutations highlighted the potential of targeting KRAS-G12D. However, unlike G12C, which contains a reactive cysteine that enables covalent inhibitor binding, G12D lacks this nucleophilic residue, making it more difficult to selectively and irreversibly target. Additionally, the G12D mutation results in a charged aspartate, which can disrupt binding interactions and complicate selectivity over wild-type KRAS [7]. Due to this reason, as of the time of writing, only HRS-4642 (Jiangsu Hengrui Medicine Co., Ltd., Lianyungang, Jiangsu, China, structure undisclosed) is in a phase 1 clinical trial (NCT05533463), while some inhibitors are in preclinical development, like MRTX1133 (Mirati Therapeutics, Inc., San Diego, CA, USA, 1) [8], RMC-9805 (Revolution Medicines, Inc., Redwood City, CA, USA, structure unknown), TH-Z835 (Tsinghua University, 2) [9], KD-8 (Southern Medical University, 3) [10], BI-KRASG12D (Boehringer Ingelheim, GmbH, Ingelheim am Rhein, Germany, structure unknown), JAB-22000 (Jacobio, Pharmaceuticals Co., Ltd., Beijing, China, structure unknown) and ERAS-5024 (Erasca, Inc., San Diego, CA, USA, structure unknown) [11,12]. The advancements in KRAS-G12D inhibitor research underscore the urgent need to discover new chemical entities for therapeutic options.

**Figure 1:**
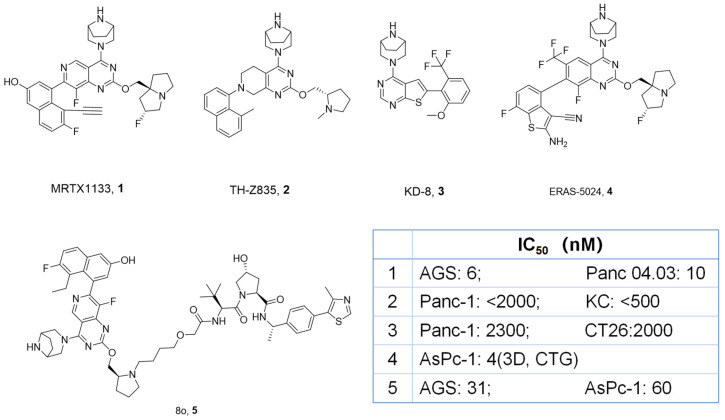
Representative KRAS-G12D inhibitors and PROTAC.

Similarly to inhibitor research, reports on protein degraders targeting KRAS-G12D remain relatively limited compared to those targeting KRAS-G12C. Recently, Zhou et al. reported a novel PROTAC (**5**) targeting KRAS-G12D. This degrader employed a VHL ligand as the E3 ubiquitin ligase-binding moiety, while using a modified structure of MRTX1133 as the warhead. To enhance membrane permeability, the pharmacophore was substantially optimized [13]: the alkynyl group on the naphthalene ring was replaced with an ethyl group, while the fused ring in the alicyclic ring was replaced with a monocyclic ring (Figure 1). The research results demonstrated that **5** could selectively and rapidly degrade the KRAS-G12D protein, and it exhibited favorable pharmacokinetic and pharmacodynamic profiles. In a tumor inhibition study, subcutaneous administration once a day for 22 days achieved a tumor inhibition rate of 68.6%. Although it is still significantly inferior compared to the 89.1% tumor inhibition rate of MRTX1133, it can still represent a promising complementary therapeutic strategy to inhibitors.

In this work, we focused on exploring a wider range of chemical space and pharmacophore effects on the KRAS-G12D protein, leveraging non-covalent interactions and targeting specific pockets through structure-based drug design. Eleven new compounds were designed and synthesized. The in vitro cell antiproliferative activity and KRAS-G12D-inhibitory activities were evaluated; simultaneously, the interactions of the representative compound and the KRAS-G12D protein were analyzed through Sybyl X V2.0 software. Then, one of the compounds was chosen to be transformed into two PROTACs, whose in vitro cell antiproliferative activity was evaluated.

## 2. Results and Discussion

### 2.1. Drug Design

We initially noticed that the inhibitors commonly consist of four parts: an aromatic core and three pharmacophores. The aromatic core may be heteromonocyclic, biheterocyclic, or hetero-tricyclic. Nitrogen-containing heterocycles are key to drug development due to their bioactivity. They function alone with various substituents or as part of polycyclic systems, making them valuable for designing diverse therapeutics [14,15]; one of the pharmacophores acts as a warhead which can form a hydrogen bond with the mutational ASP12 and the two others occupy two pockets [16,17]. Meanwhile, the oral absorption and other physicochemical properties, such as LogP, can be improved by introducing an amido bond. Based the principle described above, compounds **10a**–**k** were designed. Their aromatic cores include pyrimidine, pyrido[4,3-d]pyrimidine and 5,6,7,8-tetrahydroprodo[3,4-d]pyrimidine.

### 2.2. Chemistry

#### 2.2.1. Inhibitors

**Scheme 1:**
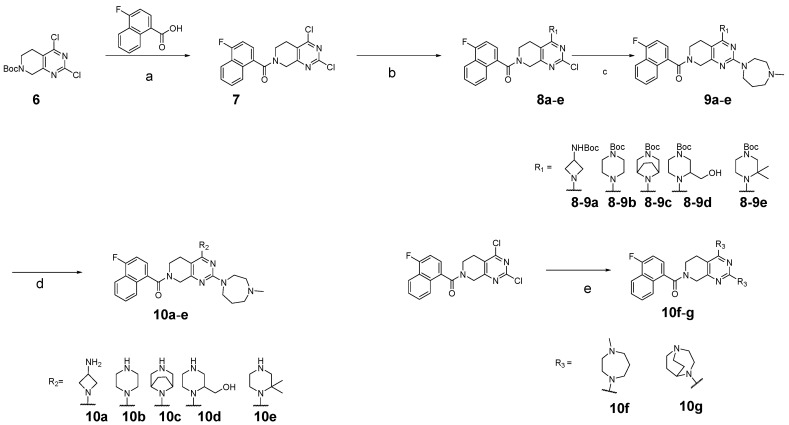
The synthesis of **10a**−**10g**. Reagents and conditions: (a) TFA, DCM, r.t.; HATU, DIEA, DMF, 0 °C~r.t.; (b) DIEA, DMF, 50 °C; (c) Cs_2_CO_3_, DMF, 120 °C; (d) TFA, DCM, r.t.; (e) Cs_2_CO_3_, DMF, 120 °C.

**Scheme 2:**
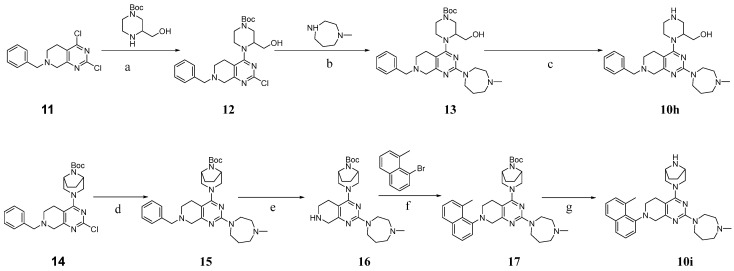
The synthesis of **10h** and **10g**. Reagents and conditions: (a) DIEA, DMF, 60 °C; (b) Cs_2_CO_3_, DMF, 120 °C; (c) TFA, DCM, r.t.; (d) Cs_2_CO_3_, DMF, 120 °C; (e) Pd/C, H_2_, r.t.; (f) Pd_2_(dba)_3_, Xantphos, Cs_2_CO_3_, 1,4-dioxane, 100 °C; (g) TFA, DCM, r.t.

**Scheme 3:**
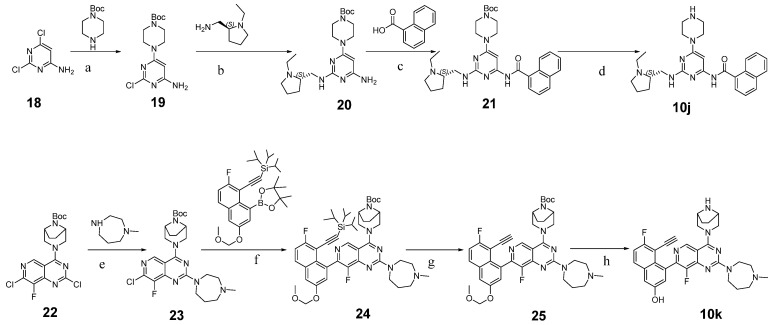
The synthesis of **10j** and **10k**. Reagents and conditions: (a) DIEA, DMF, 50 °C; (b) Cs_2_CO_3_, DMF, 120 °C; (c) HATU, DIEA, r.t. ~50 °C; (d) TFA, DCM, r.t.; (e) DIEA, DMF, 80 °C; (f) Ad_2_ ^n^BuP-Pd-G3, K_3_PO_4_, 1,4-dioxane, 80 °C; (g) CsF, DMF, r.t. ~50 °C; (h) HCl in 1,4-dioxane, DCM, 0 °C~r.t.

The general syntactic route of the designed compounds is shown in Scheme 1, Scheme 2 and Scheme 3.

The key intermediate **7** could be obtained through amide condensation using 4-fluoro-1-naphthoic acid and the TFA salt of 2,4-dichloro-5,6,7,8-tetrahydropyrido[3,4-d]pyrimidine, which was obtained from the commercially available starting material without purification. Then, the 4- and 2-positions of pyrimidine were substituted and the Boc group of R1 was removed to obtain compounds **10a**–**10e**. The ^1^H NMR spectra showed the rich signals of CH_2_ and CH_3_ at 1.50–5.00 ppm and signals of naphthalene at 7.0–8.5 ppm. Compounds **10f** and **10g** can be synthesized through a one-step process from intermediate **7,** since the two nucleophilic groups are identical and the peak characteristics in the ^1^H NMR spectra are similar to those of compounds **10a**–**10e**. To obtain intermediate **17**, which is subjected to the Buchwald–Hartwig cross-coupling reaction, we chose Bn-protected **14** as the starting material, because the Bn can be removed by means of hydrogenation without effects on the Boc group of **15**. Though the amino pyrimidine derivative **19** could be synthesized easily through two substitution reactions, the following amide condensation was difficult (poor yield). This is because the nucleophilicity of the amino was too weak. The synthetic route of compound **10k** followed that of MRTX1133 [8]. High-resolution mass spectrometry (HRMS) characterized the exact mass and elemental composition of all the inhibitors.

#### 2.2.2. PROTACs

**Scheme 4:**
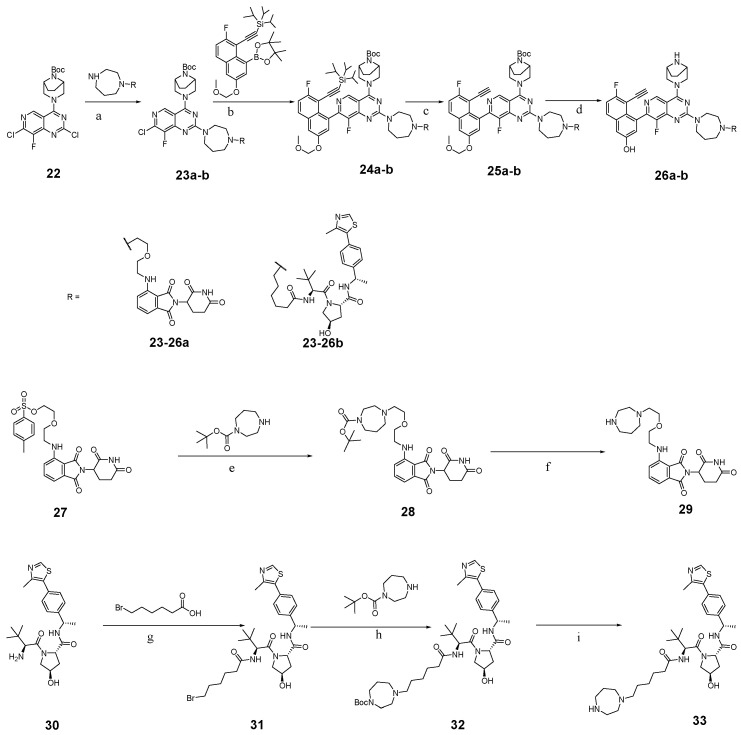
The synthesis of **26a** and **26b**. Reagents and conditions: (a) DIEA, DMF, 80 °C; (b) Ad_2_ ^n^BuP-Pd-G3, K_3_PO_4_, 1,4-dioxane, 80 °C; (c) CsF, DMF, r.t. ~50 °C; (d) TFA, DCM; (e) DIEA, DMSO, 70 °C; (f) TFA, DCM, r.t.; (g) HATU, DIEA, DMF, r.t.; (h) DIEA, DMSO, 70 °C; (i) TFA, DCM, r.t.

As shown in Scheme 4, the PROTACs **26a** and **26b** were synthesized via the key intermediates 29 and 33, respectively. Upon obtaining intermediates **23a** and **23b**, the target compounds were prepared following a procedure analogous to that employed for the synthesis of compound **10k**. The final products were characterized and confirmed by ^1^H NMR or HRMS.

### 2.3. Biological Evaluation

The antiproliferative potency of the newly synthesized compounds was investigated using a CCK-8 assay with MRTX1133 as the positive control. It has been reported that Panc1 is a KRAS-G12D-mutated human pancreatic cell and that A549 is a KRAS WT cell; meanwhile, HCT116 cells carry the KRAS-G13D mutation [10,18,19,20]. Therefore, we used these cell lines to analyze the compounds’ anticancer activity and selectivity. The results are summarized in Table 1. As shown in Table 1, most of the compounds exhibited moderate antiproliferative activity against different KRAS-mutated cells. Compound **10c** presented moderated selectivity toward KRAS-G12D-mutated Panc1 cells (IC_50_ = 1.40 μM) over KRAS-G13D-mutated HCT116 cells (IC_50_ = 5.13 μM) and the wild-type A549 cells (IC_50_ = 6.88 μM).

For the core 5,6,7,8-tetrahydroprodo[3,4-d]pyrimidine, the compound with 3,8-diazabicyclo[3.2.1]octane at position 1 (**10c**) is better than those with piperazine, azetidin-3-amine, and piperazin-2-ylmethanol (**10a**, **10b**, **10d** and **10f**). The IC_50_ for **10c** is 1.40 μM vs. 6.17, 5.87, >20 and 3.48 μM for **10a**, **10b**, **10d** and **10f** in the Panc1 cell line, respectively. The above IC_50_s also demonstrate the importance of protonating the central nitrogen and achieving appropriate spatial conformation for the compound to interact productively with the mutant Asp12 side chain. The superior potency of compound **10c**, featuring a rigid and basic 3,8-diazabicyclo[3.2.1]octane moiety at position 1, indicates that both conformational preorganization and the ability to engage in electrostatic interactions (e.g., via protonated nitrogen) are critical for effective binding to KRAS-G12D-mutated proteins. In contrast, more flexible or less basic analogs such as piperazine (**10a**), azetidine (**10b**), and hydroxymethyl-substituted piperazine (**10f**) showed diminished activity, suggesting that rigid bicyclic scaffolds may favor productive interaction with key residues like Asp12 through both spatial orientation and charge complementarity. For the compounds **10c** and **10i**, amide and amine can be found at their position 5, with IC_50_s of 1.40 μM vs. 3.92 μM, respectively. This shows that the amide group in this position can help to improve the antiproliferative activity, though most inhibitors carry an amine group like TH-Z835 [9]. Interestingly, a F-containing group [21] may also have a positive effect on the activity. Fluorine atoms are known to modulate electronic distribution and enhance binding interactions, while also improving metabolic stability. This indicates that fluorinated analogs warrant further investigation to optimize the drug-like properties of the lead series. These observations provide valuable insights for the future structure-based optimization of KRAS-targeted inhibitors.

**Figure 2:**
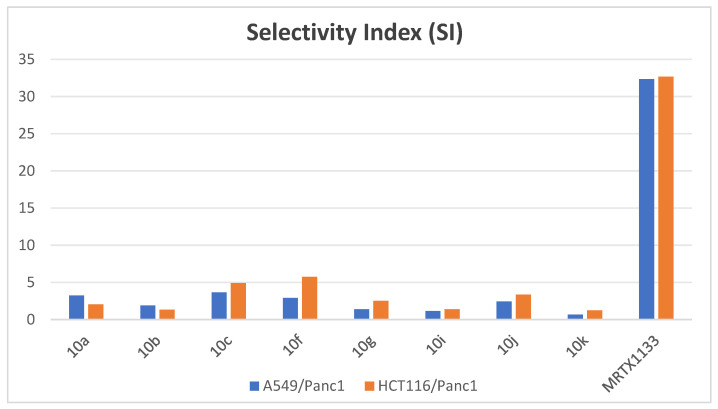
The selectivity index (SI) of the inhibitors.

The bar chart of the selectivity indices of these compounds can further illustrate the properties of these compounds, as shown in Figure 2. Compounds **10c** and **10f** showed strong selectivity indices, with the highest values being 4.9 and 5.7, respectively. Although there is still a considerable gap compared with MRTX1133, they are still worthy of further research.

Furthermore, the inhibitory activities of the eleven novel compounds against KRAS-G12D were tested (Table S1). Among them, compound **10k** showed high inhibition of KRAS-G12D with an IC_50_ of 0.009 μM, while others showed lower IC_50_ values (>10 μM). This result demonstrated that compound **10k** kept most of its key interactions with the KRAS-G12D protein to inhibit the protein’s interaction with the downstream protein. However, to our amazement, compound **10c** exhibited lower KRAS-G12D-protein-inhibitory (IC_50_ > 10 μM) activity than compound **10k** (IC_50_ = 0.009 μM) and MRTX1133 (IC_50_ = 0.0004 μM), but the antiproliferative activity and the selectivity of compound **10c** were significantly better than that of the other two compounds. These results suggest that compound **10c** may exhibit off-target effects in cells, as the KRAS protein is involved in multiple signaling pathways. Moreover, off-target toxicity of small-molecule compounds is both quite common and highly valuable [22,23].

After we obtained **26a** and **26b**, cell proliferation experiments were conducted using cell lines carrying KRAS-G12D mutations, and the results are shown in Table 2. The PROTAC targeting KRAS-G12D designed in this section did not show any advantages over its warhead and the small-molecule inhibitor MRTX1133 in human gastric cancer AGS cells with high expression of KRAS-G12D and human metastatic pancreatic cancer ASPC-1 cells. This might be due to the fact that after developing the warhead into a PROTAC, its membrane permeability was significantly affected [24], preventing it from entering the cells to exert its ubiquitination and protein degradation effects, or after entering the cells, the length and type of the linker were not conducive to the formation of a ternary complex. In the future, more types of linkers can be designed to adjust the lipophilic–hydrophilic balance coefficient of the molecule to optimize its membrane permeability and protein degradation ability [25]. In addition, it should be acknowledged that only two cancer cell lines were selected for testing in this experiment, which is insufficient to comprehensively evaluate these PROTACs. This is also one of the limitations of this experiment.

### 2.4. Molecular Docking

**Figure 3:**
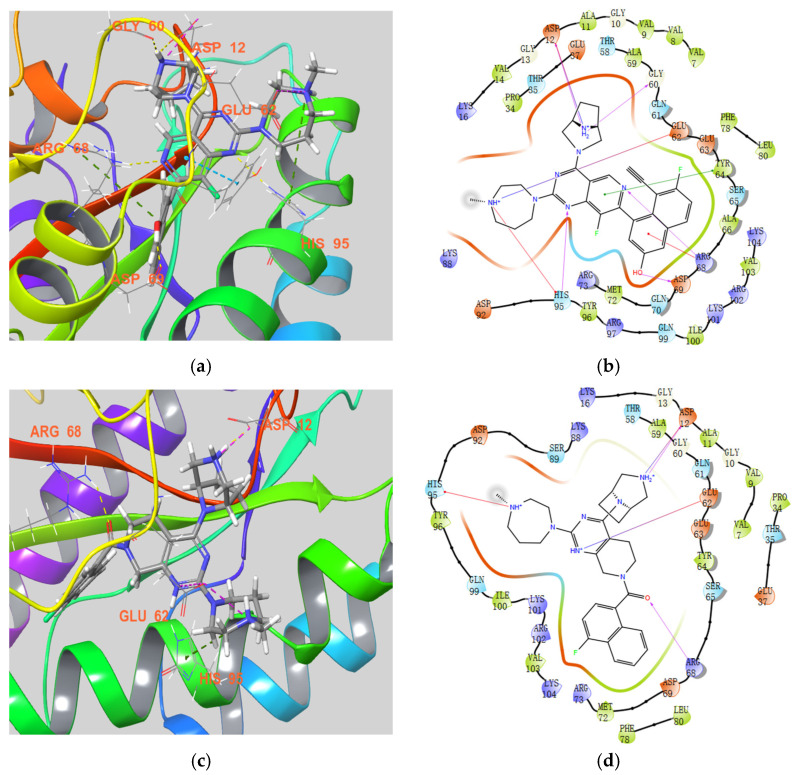

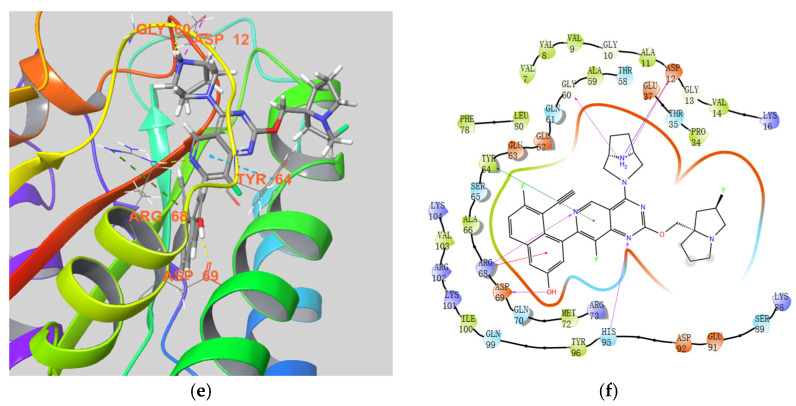
Binding diagram of **10k** (**a**), **10c** (**c**) and MRTX1133 (**e**) within the KRAS-G12D protein cavity (PDB 7RPZ); 2D view of interactions between **10k** (**b**), **10c** (**d**) and MRTX1133 (**f**) with KRAS-G12D residues.

To gain deeper insights into the interactions between the compounds and the KRAS-G12D protein, compound **10k** and **10c** were selected for molecular docking studies, with the docking of MRTX1133 acting as the validation of the protocol. As shown in Figure 3, **10k** effectively occupied the protein binding pocket, with the *N*-methyl group of homopiperazine extending into the solvent-exposed region. This position provides a strategic site for linker arm installation in PROTAC design, consistent with current strategies in targeted protein degradation approaches [26]. The secondary amine of the piperazine moiety, upon quaternization, functioned as both a hydrogen bond acceptor and donor, forming critical interactions with the key residues ASP12 and GLY60, which are crucial for selective KRAS-G12D engagement [27]. Additionally, the nitrogen atom in the homopiperazine ring could undergo quaternization to establish pi-cation with HIS95, a type of non-covalent force increasingly recognized for enhancing binding affinity and specificity in small-molecule design [28]. More importantly, this kind of interaction was not observed in the docking of MRTX1133. The phenolic hydroxyl group on the naphthalene ring of **10k** acted as a hydrogen bond donor interacting with ASP69, while the pyridyl nitrogen atom functioned as a hydrogen bond acceptor engaging ARG68. These multiple polar interactions collectively stabilize the ligand–protein complex and highlight the potential of compound **10k** as a valuable lead for KRAS-G12D-targeted therapies.

Regarding **10c**, due to the absence of a hydroxyl group on the naphthalene ring compared with **10k** and MRTX1133, no hydrogen bond was observed to form with ASP69. However, interestingly, the carbonyl oxygen of the amide bond, acting as a hydrogen bond acceptor, formed a crucial hydrogen bond with ARG68. Furthermore, upon closer examination, the docking scores of **10k**, **10c** and MRTX1133 with KRAS-G12D were −12.46, −8.94 and −12.33, respectively. These results also, to a certain extent, reflect the results of the inhibitory activities of these compounds against the KRAS-G12D enzyme.

Although molecular docking provided initial insights into the binding interactions, we acknowledge that molecular dynamics (MD) simulations could offer a more detailed assessment of binding stability. Due to time and resource limitations, MD simulations were not performed in this study. We plan to include such analyses in future work to further support our findings.

## 3. Materials and Methods

### 3.1. General Instruments

All of the analytical grade chemical reagents used in this study were purchased from commercial sources (Beijing Yusheng Chemical Co., Ltd., Beijing, China & Beijing Tongguang Fine Chemicals Co., Ltd., Beijing, China) and used without further purification. The advancement of the reaction was tracked through thin-layer chromatography (TLC) performed on F-254 fluorescent silica gel plates, with observation conducted under ultraviolet illumination. ^1^H and ^13^C spectra were assessed using JEOL^®^ 400 MHz or JEOL^®^ 600 MHz. Ms spectra were assessed using Waters Quattro Mictro^®^ Quadrupole (Waters Co., Milford, MA, USA) or Shimadzu LCMS-2010 (Shimadzu Co., Kyoto, Japan). Silica gel plates coated with GF254 acrylic adhesive were used to monitor the chemical reactions, and the spots were visualized under UV light at a wavelength of 254 nm.

### 3.2. Chemistry

#### 3.2.1. (2,4-Dichloro-5,8-dihydropyrido[3,4-d]pyrimidin-7(6H)-yl)(4-fluoronaphthalen-1-yl)methanone) (**7**)

In a single-necked flask, under room-temperature conditions, tert-butyl-2,4-dichloro-5,8-dihydropyrido[3,4-d]pyrimidine (1.00 g, 3.29 mmol) was suspended in dichloromethane (10 mL). As stirring was ongoing, trifluoroacetic acid (5 mL) was introduced, and the mixture was stirred continuously at this temperature for approximately 2 h. The dichloromethane and excess trifluoroacetic acid were removed using a rotary evaporator, yielding the trifluoroacetate salt of 2,4-dichloro-5,6,7,8-tetrahydropyrido[3,4-d]pyrimidine. In another single-necked flask, 4-fluoro-1-naphthoic acid (0.63 g, 3.29 mmol) was suspended in DMF (*N*,*N*-dimethylformamide, 10 mL) and cooled to around 0 °C using an ice-salt bath. HATU (O-(7-azabenzotriazol-1-yl)-*N*,*N*,*N*′,*N*′-tetramethyluronium hexafluorophosphate, 1.50 g, 3.95 mmol) and DIEA (*N*,*N*-Diisopropylethylamine, 1.28 g, 9.87 mmol) were added sequentially and slowly. The reaction mixture was stirred continuously at this temperature for about 10 min, after which a DMF (5 mL) solution of the aforementioned trifluoroacetate salt was introduced drop by drop. Following this addition, the reaction-derived mixture was allowed to reach the temperature of the surroundings naturally and was stirred at room temperature for an additional 16 h. The completion of the reaction was monitored by means of TLC (thin-layer chromatography). The reaction mixture was then slowly poured into water (50 mL) to form a slurry, which was filtered. Water (20 mL × 3) was used to wash the filter cake, yielding 0.96 g of **7** (white solid, yield: 78.05%).

#### 3.2.2. Tert-butyl 4-(2-chloro-7-(4-fluoro-1-naphthoyl)-5,6,7,8-tetrahydropyrido[3,4-d]pyrimidin-4-yl)piperazine-1-carboxylate (**8b**)

In a single-necked flask, **7** (100 mg, 0.27 mmol) was dissolved in DMF (5 mL). *N*-Boc-piperazine (50 mg, 0.27 mmol) and DIEA (70 mg, 0.54 mmol) were added sequentially. After the addition was complete, the mixture of the reaction was agitated at a temperature of 50 °C for approximately 5 h. The completion of the reaction was monitored by means of TLC. Subsequently, ethyl acetate (50 mL) and water (20 mL) were used to extract the reaction mixture. The organic layer was rinsed three times with 20 mL portions of saturated saline solution. Subsequently, it was dehydrated using anhydrous sodium sulfate and then reduced in volume via rotary evaporation. Silica gel column chromatography was utilized to purify the crude product (petroleum ether:ethyl acetate = 5:1 to 3:1), yielding 105 mg of **8b** (white solid, yield: 74.47%). ^1^H NMR (600 MHz, Chloroform-d) δ 8.23–8.10 (m, 1H), 7.84 (m, 1H), 7.65–7.53 (m, 2H), 7.47–7.36 (m, 1H), 7.24–7.14 (m, 1H), 5.22–4.68 (m, 1H), 4.38–4.22 (m, 1H), 4.06 (m, 1H), 3.62–3.32 (m, 9H), 2.90–2.74 (m, 1H), 2.49 (m, 1H), 1.48 (m, 9H). ^13^C NMR (100 MHz, Chloroform-d) δ 169.3, 165.9, 162.7, 161.0, 158.0, 154.8, 128.5, 127.1, 125.2, 125.1, 124.8, 124.4, 124.1, 123.94, 121.4, 113.1, 109.2, 109.0, 80.5, 51.5, 57.6, 47.6, 46.5, 44.2, 39.4, 28.5, 26.4.

#### 3.2.3. Tert-butyl 4-(7-(4-fluoro-1-naphthoyl)-2-(4-methyl-1,4-diazepan-1-yl)-5,6,7,8-tetrahydropyrido[3,4-d]pyrimidin-4-yl)piperazine-1-carboxylate (**9b**)

In a single-necked flask, **8b** (50 mg, 0.10 mmol), Cs_2_CO_3_ (65 mg, 0.20 mmol), and *N*-methyl-homopiperazine (23 mg, 0.20 mmol) were mixed in DMF (5 mL). The mixture underwent stirring at 120 °C over a period of 16 h, and the reaction progress was monitored by means of TLC. After the starting materials were mostly consumed, the reactant blend was subjected to extraction using ethyl acetate (50 mL) and water (20 mL). The organic layer was then rinsed thrice with 10-milliliter aliquots of saturated saltwater. After that, it was dehydrated with anhydrous sodium sulfate and finally had its volume reduced. The crude product was purified via thin-layer chromatography using a solvent system of dichloromethane/methanol = 10:1, yielding 38 mg of **8b** (white solid, yield: 63.33%). ^1^H NMR (400 MHz, Chloroform-d) δ 8.21–8.09 (m, 1H), 7.91–7.78 (m, 1H), 7.65–7.51 (m, 2H), 7.45–7.35 (m, 1H), 7.17 (q, *J* = 9.6 Hz, 1H), 4.80 (m, 1H), 4.14 (d, *J* = 9.2 Hz, 1H), 4.00 (m, 2H), 3.85–3.75 (m, 2H), 3.66–3.58 (m, 1H), 3.56–3.50 (m, 2H), 3.45 (t, *J* = 4.8 Hz, 2H), 3.33 (t, *J* = 4.8 Hz, 3H), 3.24 (d, *J* = 5.8 Hz, 2H), 2.88–2.80 (m, 1H), 2.78–2.71 (m, 2H), 2.70–2.58 (m, 2H), 2.49 (s, 1H), 2.39 (d, *J* = 5.8 Hz, 3H), 2.16–1.93 (m, 2H), 1.47 (d, *J* = 11.6 Hz, 9H). ^13^C NMR (101 MHz, Chloroform-d) δ 169.2, 165.8, 161.7, 161.0, 159.4, 154.9, 131.3, 130.1, 128.2, 126.9, 124.8, 124.1, 120.6, 109.0, 103.9, 103.4, 80.1, 58.3, 57.1, 51.6, 51.4, 47.7, 47.5, 46.3, 45.4, 45.0, 44.6, 40.4, 29.8, 28.5, 26.8, 25.6.

#### 3.2.4. (4-Fluoronaphthalen-1-yl)(2-(4-methyl-1,4-diazepan-1-yl)-4-(piperazin-1-yl)-5,8-dihydropyrido[3,4-d]pyrimidin-7(6H)-yl)methanone (**10b**)

In a single-necked flask, **9b** (25 mg, 0.04 mmol) was solubilized in 5 mL of DCM. As the solution was being stirred, 2 mL of trifluoroacetic acid was gradually added drop by drop. Once the addition of trifluoroacetic acid was finished, the resulting reaction mixture was continuously agitated at an ambient temperature for 2 h. The disappearance of the starting material was confirmed by TLC. The solvent was then removed using a rotary evaporator. The remaining substance was neutralized using a saturated sodium bicarbonate solution. Subsequently, extraction was carried out with a combination of ethyl acetate (30 mL) and water (10 mL). The organic layer was then rinsed three times, each time with 10 mL of saturated saline solution. After that, it was dried using anhydrous sodium sulfate and then concentrated. The unrefined product obtained was then purified through the use of silica gel column chromatography (dichloromethane/methanol = 10:1 to 5:1), yielding 15 mg of **10b** (white solid, yield: 71.43%). Mp 128.5–131.2 °C. ^1^H NMR (400 MHz, Chloroform-d) δ 8.20–8.11 (m, 1H), 7.90–7.81 (m, 1H), 7.63–7.52 (m, 2H), 7.45–7.36 (m, 1H), 7.22–7.12 (m, 1H), 4.82 (d, *J* = 8.6 Hz, 1H), 4.13 (d, *J* = 9.2 Hz, 1H), 4.01 (t, *J* = 5.4 Hz, 1H), 3.92 (t, *J* = 4.9 Hz, 1H), 3.85–3.72 (m, 2H), 3.67–3.59 (m, 1H), 3.40–3.32 (m, 3H), 3.29–3.20 (m, 2H), 2.98 (t, *J* = 4.9 Hz, 2H), 2.94–2.86 (m, 2H), 2.74 (t, *J* = 5.4 Hz, 2H), 2.65–2.56 (m, 2H), 2.55–2.49 (m, 1H), 2.45–2.30 (m, 4H), 2.04–1.85 (m, 2H), 1.41–1.33 (m, 1H). ^13^C NMR (101 MHz, Chloroform-d) δ 169.2, 165.7, 161.4, 160.7, 159.1, 158.1, 131.4, 130.3, 128.3, 126.9, 124.9, 124.1, 121.2, 109.2, 103.6, 64.3, 58.6, 57.4, 52.0, 49.0, 47.6, 46.7, 46.0, 45.7, 45.3, 40.5, 29.8, 27.5. ESI-HRMS *m*/*z* calculated for C_28_H_35_FN_7_O^+^ 504.2882 [M + H]^+^, found 504.2886 [M + H]^+^.

#### 3.2.5. Tert-butyl 4-(2-chloro-7-(4-fluoro-1-naphthoyl)-5,6,7,8-tetrahydropyrido[3,4-d]pyrimidin-4-yl)-3-(hydroxymethyl)piperazine-1-carboxylate (**8d**)

The synthesis of compound **8d** was carried out following a procedure analogous to that of **8b**. The starting material, 1-Boc-3-hydroxymethylpiperazine, was used as the reactant. The purification process involved column chromatography with a gradient elution system of petroleum ether/ethyl acetate (3:1 to 1:1). The target compound **8d** was obtained as a white solid with a yield of 62.92%. ^1^H NMR (400 MHz, Chloroform-d) δ 8.21–8.09 (m, 1H), 7.89–7.76 (m, 1H), 7.65–7.53 (m, 2H), 7.44–7.36 (m, 1H), 7.21–7.11 (m, 1H), 5.15–4.76 (m, 1H), 4.27 (s, 2H), 4.21–3.83 (m, 4H), 3.83–3.68 (m, 2H), 3.67–3.47 (m, 1H), 3.47–3.21 (m, 2H), 3.19–2.96 (m, 2H), 2.86–2.80 (m, 1H), 2.64–2.36 (m, 1H), 1.46 (d, *J* = 12.0 Hz, 9H).

#### 3.2.6. Tert-butyl 4-(7-(4-fluoro-1-naphthoyl)-2-(4-methyl-1,4-diazepan-1-yl)-5,6,7,8-tetrahydropyrido[3,4-d]pyrimidin-4-yl)-3-(hydroxymethyl)piperazine-1-carboxylate (**9d**)

Compound **9d** was synthesized using a similar procedure to that employed for **9b**, with **8d** serving as the starting material. The target compound **9d** was obtained as a white solid with a yield of 64.38%. ^1^H NMR (400 MHz, Methanol-d_4_) δ 8.28–8.08 (m, 1H), 7.87–7.70 (m, 1H), 7.66–7.56 (m, 2H), 7.55–7.38 (m, 1H), 7.36–7.17 (m, 1H), 4.80–4.72 (m, 1H), 4.14–3.92 (m, 4H), 3.92–3.84 (m, 2H), 3.79 (t, *J* = 6.3 Hz, 1H), 3.74–3.67 (m, 2H), 3.67–3.52 (m, 3H), 3.29–3.25 (m, 2H), 3.24–3.07 (m, 2H), 2.94–2.83 (m, 2H), 2.75–2.57 (m, 4H), 2.40 (m, 3H), 2.05–1.82 (m, 2H), 1.44 (d, *J* = 10.3 Hz, 9H). ^13^C NMR (101 MHz, Methanol-d_4_) δ 161.8, 161.4, 152.5, 151.1, 150.9, 147.4, 123.2, 121.9, 120.1, 118.9, 116.4, 116.2, 115.6, 111.9, 100.7, 95.5, 72.0, 50.6, 49.6, 48.5, 47.5, 43.3, 36.9, 36.8, 36.0, 33.8, 31.6, 19.1, 18.3, 16.3.

#### 3.2.7. (4-Fluoronaphthalen-1-yl)(4-(2-(hydroxymethyl)piperazin-1-yl)-2-(4-methyl-1,4-diazepan-1-yl)-5,8-dihydropyrido[3,4-d]pyrimidin-7(6H)-yl)methanone (**10d**)

Referring to the process for **10b**, **10d** was obtained as a white solid with a yield of 79.32%. Mp 183.2–184.7 °C. ^1^H NMR (600 MHz, Methanol-d_4_) δ 8.24–8.15 (m, 1H), 7.86 (d, *J* = 25.0 Hz, 1H), 7.75–7.60 (m, 2H), 7.52 (d, *J* = 25.0 Hz, 1H), 7.30 (t, *J* = 9.1 Hz, 1H), 4.24–4.06 (m, 2H), 4.04–3.84 (m, 5H), 3.81–3.60 (m, 4H), 3.23–3.07 (m, 3H), 3.06–2.95 (m, 3H), 2.93–2.72 (m, 5H), 2.51 (m, 4H), 2.11–1.91 (m, 3H), 1.60 (s, 1H). ^13^C NMR (151 MHz, Methanol-d_4_) δ 170.0, 165.7, 160.9, 159.3, 158.4, 131.0, 130.0, 129.5, 128.2, 127.0, 124.5, 124.3, 123.6, 120.5, 108.7, 104.0, 72.5, 63.1, 59.9, 57.6, 56.5, 54.2, 51.4, 44.8, 44.1, 42.6, 40.1, 29.4, 26.0. ESI-HRMS *m*/*z* calculated for C_29_H_37_FN_7_O_2_^+^ 534.2987 [M + H]^+^, found 534.2995 [M + H]^+^.

#### 3.2.8. Tert-butyl 4-(2-chloro-7-(4-fluoro-1-naphthoyl)-5,6,7,8-tetrahydropyrido[3,4-d]pyrimidin-4-yl)-3,3-dimethylpiperazine-1-carboxylate (**8e**)

**8e** was synthesized and processed using a method similar to that of **8b**. The starting material, 1-Boc-3,3-dimethylpiperazine, was used as the reactant. The desired product was isolated as a white solid in 64.28% yield.

#### 3.2.9. Tert-butyl 4-(7-(4-fluoro-1-naphthoyl)-2-(4-methyl-1,4-diazepan-1-yl)-5,6,7,8-tetrahydropyrido[3,4-d]pyrimidin-4-yl)-3,3-dimethylpiperazine-1-carboxylate (**9e**)

The preparation of compound **9e**, including its synthesis and post-treatment, was conducted using a method similar to the one employed for intermediate **9b**. Ultimately, the desired compound **9e** was isolated as a white solid, achieving a yield of 44.18%.

#### 3.2.10. (4-(2,2-Dimethylpiperazin-1-yl)-2-(4-methyl-1,4-diazepan-1-yl)-5,8-dihydropyrido[3,4-d]pyrimidin-7(6H)-yl)(4-fluoronaphthalen-1-yl)methanone (**10e**)

Compound **10e** was synthesized and post-treated using a method similar to that for intermediate **10b**, yielding a white solid at 78.65%. Mp 151.3–133.8 °C. ESI-HRMS *m*/*z* calculated for C_29_H_37_FN_7_O_2_^+^ 532.3195 [M + H]^+^, found 532.3172 [M + H]^+^.

#### 3.2.11. Tert-butyl 4-(6-amino-2-chloropyrimidin-4-yl)piperazine-1-carboxylate (**19**)

**19** was synthesized and processed post-synthetically using a method akin to that for intermediate **8b**. The starting material, 4-amino-2,6-dichloropyrimidine, was used as the reactant. The target compound **19** was obtained as a white solid with a yield of 79.28%. ^1^H NMR (400 MHz, DMSO-d_6_) δ 6.79 (s, 2H), 5.75 (s, 1H), 3.69–3.50 (m, 4H), 3.33 (d, *J* = 4.2 Hz, 4H), 1.41 (s, 9H). ^13^C NMR (101 MHz, DMSO-d_6_) δ 165.0, 160.8, 158.1, 153.9, 92.4, 79.0, 43.0, 28.1.

#### 3.2.12. Tert-butyl (S)-4-(6-amino-2-(((1-ethylpyrrolidin-2-yl)methyl)amino)pyrimidin-4-yl)piperazine-1-carboxylate (**20**)

Compound **20** was synthesized similarly to **9b**, using (S)-2-aminomethyl-1-ethylpyrrolidine as the starting material. The product was isolated as a white solid with 58.92% yield. ^1^H NMR (400 MHz, Chloroform-d) δ 5.15 (s, 1H), 4.95 (s, 1H), 4.29 (s, 2H), 3.68 (m, 4H), 3.42 (m, 5H), 3.28 (d, *J* = 9.3 Hz, 2H), 2.97–2.71 (m, 2H), 2.40–2.18 (m, 2H), 2.01–1.64 (m, 4H), 1.47 (s, 9H), 1.14 (t, *J* = 7.2 Hz, 3H). ^13^C NMR (101 MHz, Chloroform-d) δ 164.4, 164.0, 161.9, 155.1, 79.8, 74.7, 63.8, 53.7, 48.9, 43.7, 42.8, 28.5, 22.9, 13.4.

#### 3.2.13. Tert-butyl (S)-4-(6-(1-naphthamido)-2-(((1-ethylpyrrolidin-2-yl)methyl)amino)pyrimidin-4-yl)piperazine-1-carboxylate (**21**)

In a single-necked flask under nitrogen protection, 100 mg (0.53 mmol) of 4-fluoro-1-naphthoic acid was dispersed in 5 mL of DMF. An ice-salt bath was employed to bring the temperature of the resulting mixture down to around 0 °C. HATU (240 mg, 0.63 mmol) and DIEA (164 mg, 1.26 mmol) were added slowly and sequentially. Once the addition process concluded, the blend was continuously agitated at the same temperature for 10 min. Subsequently, a solution composed of **20** (244 mg, 0.80 mmol) in 2 mL of DMF was slowly introduced drop by drop. After that, the reaction mixture was left to reach room temperature on its own and then transferred to a 50 °C oil bath, where it was stirred for 16 h. The mixture from the reaction was subjected to extraction using a combination of ethyl acetate (50 mL) and water (20 mL). The organic layer obtained was then rinsed with a saturated sodium chloride solution (20 mL × 3). Subsequently, it was dried using anhydrous sodium sulfate and then the solvent was removed to concentrate the mixture. The resulting unpurified product was then refined through the application of silica gel plate chromatography (dichloromethane/methanol = 10:1), yielding **21** (140 mg, white solid, yield: 45.90%). ^1^H NMR (600 MHz, Chloroform-d) δ 9.10 (s, 1H), 8.13 (dd, *J* = 8.4, 1.4 Hz, 1H), 8.04 (m, 1H), 7.92 (d, *J* = 8.4 Hz, 1H), 7.59–7.53 (m, 2H), 7.49 (s, 1H), 7.43 (dd, *J* = 7.8, 5.2 Hz, 1H), 7.16 (dd, *J* = 9.9, 7.8 Hz, 1H), 3.84–3.78 (m, 1H), 3.76 (t, *J* = 5.4 Hz, 4H), 3.63 (m, 1H), 3.44 (t, *J* = 5.2 Hz, 5H), 3.35 (s, 1H), 3.11 (dd, *J* = 13.0, 7.0 Hz, 1H), 2.84–2.73 (m, 2H), 2.04 (s, 1H), 1.86 (s, 2H), 1.66 (s, 1H), 1.46 (s, 9H), 1.16 (s, 3H). ^13^C NMR (151 MHz, Chloroform-d) δ 164.7, 164.2, 162.8, 160.1, 158.9, 154.9, 136.0, 131.2, 128.6, 127.4, 125.1, 124.9, 124.2, 121.1, 109.6, 91.8, 80.3, 66.5, 53.7, 50.1, 43.6, 42.1, 36.6, 31.6, 29.8, 28.5, 22.6, 11.3.

#### 3.2.14. (S)-*N*-(2-(((1-Ethylpyrrolidin-2-yl)methyl)amino)-6-(piperazin-1-yl)pyrimidin-4-yl)-1-naphthamide (**10j**)

Compound **10j** was prepared using a method similar to **10b**, yielding 75.56% of the product as a white solid. Mp 133.7–136.3 °C. ^1^H NMR (400 MHz, Chloroform-d) δ 8.17–8.11 (m, 1H), 7.99 (d, *J* = 8.0 Hz, 1H), 7.62–7.51 (m, 2H), 7.44 (dd, *J* = 8.0, 5.3 Hz, 1H), 7.16 (t, *J* = 10.2, 8.0 Hz, 1H), 3.83 (t, *J* = 5.3 Hz, 4H), 3.71–3.61 (m, 1H), 3.62–3.45 (m, 2H), 3.20–3.05 (m, 1H), 2.91 (t, *J* = 5.3 Hz, 4H), 2.83–2.75 (m, 1H), 2.66–2.49 (m, 1H), 2.42–2.32 (m, 1H), 2.08–1.91 (m, 3H), 1.74–1.61 (m, 1H), 1.43–1.39 (m, 1H), 0.89–0.85 (m, 3H). ESI-HRMS *m*/*z* calculated for C_26_H_33_FN_7_O^+^ 478.2725 [M + H]^+^, found 478.2775 [M + H]^+^.

#### 3.2.15. Tert-butyl 4-(7-benzyl-2-chloro-5,6,7,8-tetrahydropyrido[3,4-d]pyrimidin-4-yl)-3-(hydroxymethyl)piperazine-1-carboxylate (**12**)

In a single-necked flask, 2,4-dichloro-5,6,7,8-tetrahydro-7-(phenylmethyl)-pyrido[3,4-d]pyrimidine (2.00 g, 6.80 mmol) was dissolved in DMF (20 mL). 3-Hydroxymethyl-1-Boc-piperazine (1.47 g, 6.80 mmol) and DIEA (1.77 g, 13.60 mmol) were added. The reaction mixture was stirred at 60 °C under nitrogen protection overnight, and the progress was monitored by TLC. The reaction mixture underwent liquid–liquid extraction with ethyl acetate (200 mL) and water (100 mL). After phase separation, the organic phase was washed with brine (saturated NaCl solution, 50 mL × 3) and subsequently dried over anhydrous sodium sulfate. Following solvent evaporation to concentrate the crude material, purification was achieved by means of silica gel thin-layer chromatography (petroleum ether:ethyl acetate = 3:1 to 0:1), yielding **12** (2.66 g, white solid, yield: 82.74%). ^1^H NMR (400 MHz, Chloroform-d) δ 7.36–7.32 (m, 3H), 7.32–7.27 (m, 2H), 4.26–4.19 (m, 1H), 4.18–3.96 (m, 1H), 3.93–3.74 (m, 2H), 3.73–3.67 (m, 3H), 3.67–3.53 (m, 2H), 3.29 (t, *J* = 13.0 Hz, 1H), 3.21–2.90 (m, 2H), 2.87–2.73 (m, 1H), 2.73–2.59 (m, 3H), 2.42–2.05 (m, 1H), 1.47 (s, 9H). ^13^C NMR (101 MHz, Chloroform-d) δ 171.2, 166.0, 165.8, 162.6, 157.0, 137.1, 129.2, 128.5, 127.6, 113.8, 80.7, 62.2, 60.4, 57.9, 55.7, 49.5, 42.6, 28.4, 27.3, 21.1, 14.2.

#### 3.2.16. Tert-butyl 4-(7-benzyl-2-(4-methyl-1,4-diazepan-1-yl)-5,6,7,8-tetrahydropyrido[3,4-d]pyrimidin-4-yl)-3-(hydroxymethyl)piperazine-1-carboxylate (**13**)

In a single-necked flask, **12** (1.50 g, 3.17 mmol) was dissolved in DMF (15 mL). *N*-methylhomopiperazine (722 mg, 6.34 mmol) and Cs_2_CO_3_ (2.07 g, 6.34 mmol) were added. The mixture was placed in a 120 °C oil bath and stirred for 16 h. The crude reaction mixture was partitioned between ethyl acetate and water. The ethyl acetate layer was then sequentially rinsed with saturated NaCl solution (3 × 50 mL), dried with anhydrous Na_2_SO_4_, and evaporated to dryness. Final purification was accomplished through flash column chromatography on silica gel (dichloromethane/methanol = 8:1 to 5:1), yielding the product (0.92 g, white solid, yield: 52.67%). ^1^H NMR (400 MHz, DMSO-d_6_) δ 7.40–7.18 (m, 5H), 4.78–3.94 (m, 1H), 3.94–3.66 (m, 3H), 3.66–3.52 (m, 6H), 3.52–3.37 (m, 3H), 3.30–3.04 (m, 4H), 3.04–2.75 (m, 3H), 2.63–2.51 (m, 2H), 2.45–2.25 (m, 4H), 2.17 (s, 3H), 1.75 (p, *J* = 6.0 Hz, 2H), 1.36 (s, 9H). ^13^C NMR (151 MHz, DMSO-d_6_) δ 165.1, 163.4, 159.1, 154.9, 138.7, 129.4, 128.8, 127.6, 103.6, 79.4, 62.1, 58.9, 58.6, 58.2, 57.2, 55.5, 50.9, 46.7, 45.9, 45.8, 42.6, 28.5, 27.7, 26.3.

#### 3.2.17. (1-(7-Benzyl-2-(4-methyl-1,4-diazepan-1-yl)-5,6,7,8-tetrahydropyrido [3,4-d]pyrimidin-4-yl)piperazin-2-yl)methanol (**10h**)

Compound **10h** was synthesized according to a process similar to that for **10b**. The desired compound **10h** was acquired as a white solid, achieving a yield of 75.92%. ^1^H NMR (600 MHz, Methanol-d_4_) δ 7.48 (d, *J* = 7.4 Hz, 2H), 7.39 (t, *J* = 7.4 Hz, 2H), 7.37–7.33 (m, 1H), 4.45 (s, 2H), 4.04 (s, 2H), 3.96 (s, 2H), 3.91–3.76 (m, 2H), 3.67–3.52 (m, 3H), 3.47–3.38 (m, 2H), 3.30–3.25 (m, 2H), 3.24–3.15 (m, 2H), 3.11–3.05 (m, 1H), 3.03–2.98 (m, 2H), 2.94 (d, *J* = 12.7 Hz, 1H), 2.90 (s, 3H), 2.80–2.68 (m, 2H), 2.24–2.14 (m, 2H). ^13^C NMR (151 MHz, Methanol-d_4_) δ 168.0, 162.8, 160.4, 135.9, 131.3, 129.8, 129.4, 103.2, 66.7, 62.5, 57.7, 57.0, 52.9, 50.9, 46.2, 45.9, 45.1, 44.1, 43.3, 43.2, 25.7, 21.3. Mp 113.7–116.2 °C. ESI-HRMS *m*/*z* calculated for C_25_H_38_N_7_O^+^ 452.3132 [M + H]^+^, found 452.3129 [M + H]^+^.

#### 3.2.18. (2,4-Bis(4-methyl-1,4-diazepan-1-yl)-5,8-dihydropyrido[3,4-d]pyrimidin-7(6H)-yl)(4-fluoronaphthalen-1-yl)methanone (**10f**)

In a single-necked flask, **7** (40 mg, 0.11 mmol) was suspended in DMF (5 mL). Cs_2_CO_3_ (108 mg, 0.33 mmol) and *N*-methylhomopiperazine (25 mg, 0.22 mmol) were added. The mixture was stirred at 120 °C for 16 h and the reaction progress was monitored by TLC. After the reaction was nearly complete, the mixture underwent ethyl acetate/water extraction. The organic extract underwent three brine washes (5 mL each), was dried over sodium sulfate, and evaporated. Final isolation employed silica gel column chromatography (dichloromethane/methanol = 8:1 to 5:1), yielding **10f** (45 mg, white solid, yield: 77.23%). Mp 115.5–118.2 °C. ^1^H NMR (600 MHz, Chloroform-d) δ 8.15 (t, *J* = 8.1 Hz, 1H), 7.91–7.82 (m, 1H), 7.61–7.53 (m, 2H), 7.44–7.35 (m, 1H), 7.22–7.09 (m, 1H), 4.86–4.06 (m, 2H), 4.04–3.85 (m, 2H), 3.81–3.69 (m, 3H), 3.67–3.53 (m, 4H), 3.35–3.23 (m, 1H), 2.90–2.65 (m, 4H), 2.63–2.54 (m, 4H), 2.52–2.43 (m, 2H), 2.42–2.26 (m, 6H), 2.02–1.95 (m, 2H), 1.95–1.82 (m, 2H). Mp 115.2–118.2 °C. ESI-HRMS *m*/*z* calculated for C_30_H_39_FN_7_O^+^ 532.3195 [M + H]^+^, found 532.3196 [M + H]^+^.

#### 3.2.19. (2,4-Di(1,4-diazabicyclo[3.2.2]nonan-4-yl)-5,8-dihydropyrido[3,4-d]pyrimidin-7(6H)-yl)(4-fluoronaphthalen-1-yl)methanone (**10g**)

Compound **10g** was prepared from 1,4-diazabicyclo[3.2.2]nonane following a method analogous to that used for **10f**, affording a white solid with 69.38% yield. Mp 123.6–126.2 °C. ESI-HRMS *m*/*z* calculated for C_32_H_39_FN_7_O^+^ 556.3195 [M + H]^+^, found 556.3193 [M + H]^+^.

#### 3.2.20. Tert-butyl (1-(2-chloro-7-(4-fluoro-1-naphthoyl)-5,6,7,8-tetrahydropyrido[3,4-d]pyrimidin-4-yl)azetidin-3-yl)carbamate (**8a**)

Compound **8a** was obtained using a method similar to that of **8b**, starting from 3-(Boc-amino)azetidine. The product was isolated as a white solid with 77.18% yield.

#### 3.2.21. Tert-butyl (1-(7-(4-fluoro-1-naphthoyl)-2-(4-methyl-1,4-diazepan-1-yl)-5,6,7,8-tetrahydropyrido[3,4-d]pyrimidin-4-yl)azetidin-3-yl)carbamate (**9a**)

Compound **9a** was synthesized from **8a** using a procedure similar to that for **9b**, yielding a white solid with 64.37% yield.

#### 3.2.22. (4-(3-Aminoazetidin-1-yl)-2-(4-methyl-1,4-diazepan-1-yl)-5,8-dihydropyrido[3,4-d]pyrimidin-7(6H)-yl)(4-fluoronaphthalen-1-yl)methanone (**10a**)

The synthesis of compound **10a** was carried out following a procedure analogous to that for **10b**. The starting material, **9a**, was used as the reactant. The target compound **10a** was obtained as a white solid with a yield of 81.53%. Mp 119.5–121.4 °C. ^1^H NMR (600 MHz, Methanol-d_4_) δ 8.22–8.15 (m, 1H), 7.87–7.78 (m, 1H), 7.68–7.62 (m, 2H), 7.55–7.45 (m, 1H), 7.35–7.25 (m, 1H), 4.75 (d, *J* = 10.7 Hz, 1H), 4.53–4.44 (m, 1H), 4.37 (t, *J* = 7.9 Hz, 1H), 4.23–4.08 (m, 1H), 4.04–4.00 (m, 2H), 3.95–3.91 (m, 1H), 3.88 (t, *J* = 6.4 Hz, 1H), 3.82 (s, 1H), 3.69–3.63 (m, 1H), 3.63–3.50 (m, 1H), 3.41 (dt, *J* = 11.3, 5.9 Hz, 1H), 3.25 (t, *J* = 5.3 Hz, 1H), 3.17–3.13 (m, 1H), 3.06 (s, 1H), 3.03–2.99 (m, 1H), 2.87–2.80 (m, 1H), 2.78–2.76 (m, 2H), 2.66 (s, 1H), 2.57–2.40 (m, 2H), 2.20–2.12 (m, 1H), 2.03–1.97 (m, 1H). ESI-HRMS *m*/*z* calculated for C_27_H_33_FN_7_O^+^ 490.2725 [M + H]^+^, found 490.2730 [M + H]^+^.

#### 3.2.23. Tert-butyl (1R,5S)-8-(2-chloro-7-(4-fluoro-1-naphthoyl)-5,6,7,8-tetrahydropyrido[3,4-d]pyrimidin-4-yl)-3,8-diazabicyclo[3.2.1]octane-3-carboxylate (**8c**)

**8c** was synthesized by adhering to the method used for compound **8b**. Commencing with tert-butyl 3,8-diazabicyclo[3.2.1]octane-3-carboxylate as the starting material, compound **8c** was obtained as a white solid with a yield of 71.78%. ^1^H NMR (600 MHz, Chloroform-d) δ 8.28–8.10 (m, 1H), 7.83 (m, 1H), 7.65–7.51 (m, 2H), 7.46–7.33 (m, 1H), 7.21–7.08 (m, 1H), 5.11–4.74 (m, 1H), 4.69–4.43 (m, 2H), 4.28–4.00 (m, 2H), 3.90 (dd, *J* = 37.2, 13.1 Hz, 1H), 3.75 (dd, *J* = 28.3, 13.1 Hz, 1H), 3.51–3.30 (m, 1H), 3.28–3.14 (m, 1H), 3.12–2.93 (m, 1H), 2.88–2.73 (m, 1H), 2.64–2.31 (m, 1H), 2.09–1.72 (m, 4H), 1.45 (d, *J* = 13.8 Hz, 9H).^13^C NMR (151 MHz, Chloroform-d) δ 169.2, 162.5, 162.1, 158.1, 155.9, 131.2, 129.3, 128.4, 127.0, 125.1, 124.8, 124.4, 124.1, 121.4, 111.6, 109.1, 109.0, 80.4, 55.7, 55.4, 51.5, 49.8, 48.9, 46.9, 44.1, 39.3, 28.5, 26.7, 26.1.

#### 3.2.24. Tert-butyl (1R,5S)-8-(7-(4-fluoro-1-naphthoyl)-2-(4-methyl-1,4-diazepan-1-yl)-5,6,7,8-tetrahydropyrido[3,4-d]pyrimidin-4-yl)-3,8-diazabicyclo[3.2.1]octane-3-carboxylate (**9c**)

Compound **9c** was prepared from **8c** using the same method as **9b**, affording a white solid with 59.77% yield.

#### 3.2.25. (4-((1R,5S)-3,8-Diazabicyclo[3.2.1]octan-8-yl)-2-(4-methyl-1,4-diazepan-1-yl)-5,8-dihydropyrido[3,4-d]pyrimidin-7(6H)-yl)(4-fluoronaphthalen-1-yl)methanone (**10c**)

The synthesis of compound **10c** followed the procedure for **10b**, using **9c** as the starting material, yielding **10c** (white solid, 80.55% yield). Mp 147.4–150.1 °C. ^1^H NMR (600 MHz, Chloroform-d) δ 8.21–8.11 (m, 1H), 7.91–7.82 (m, 1H), 7.62–7.49 (m, 2H), 7.47–7.34 (m, 1H), 7.23–7.05 (m, 1H), 4.82 (q, *J* = 18.5 Hz, 1H), 4.38–4.32 (m, 1H), 4.26–4.17 (m, 1H), 4.18–3.96 (m, 2H), 3.88 (s, 1H), 3.75 (d, *J* = 33.5 Hz, 2H), 3.60 (s, 1H), 3.44–3.21 (m, 1H), 3.15 (d, *J* = 12.1 Hz, 1H), 3.07 (dd, *J* = 25.4, 12.1 Hz, 1H), 2.81–2.61 (m, 4H), 2.59–2.51 (m, 2H), 2.49–2.44 (m, 1H), 2.34 (d, *J* = 47.4 Hz, 3H), 2.06–1.79 (m, 7H), 1.46–1.36 (m, 1H). ESI-HRMS *m*/*z* calculated for C_30_H_37_FN_7_O^+^ 530.3027 [M + H]^+^, found 530.3043 [M + H]^+^.

#### 3.2.26. Tert-butyl (1R,5S)-3-(7-benzyl-2-(4-methyl-1,4-diazepan-1-yl)-5,6,7,8-tetrahydropyrido[3,4-d]pyrimidin-4-yl)-3,8-diazabicyclo[3.2.1]octane-8-carboxylate (**15**)

**15** was synthesized following the procedure for **9b**, using tert-butyl 3-(3-[2-chloro-5,6,7,8-tetrahydro-7-(benzyl)pyrido[3,4-d]pyridin-4-yl])diazabicyclo[3.2.1]octane-8-carboxylate as the starting material, affording compound **23** as a white solid with 71.57% yield. ^1^H NMR (600 MHz, DMSO-d_6_) δ 7.76–7.27 (m, 4H), 4.71–4.24 (m, 2H), 4.16 (s, 2H), 4.03–3.39 (m, 9H), 3.39–3.21 (m, 6H), 3.11–2.99 (m, 2H), 2.95–2.82 (m, 1H), 2.81–2.68 (m, 3H), 2.66–2.54 (m, 1H), 2.46–1.96 (m, 2H), 1.91–1.66 (m, 3H), 1.40 (s, 9H).

#### 3.2.27. Tert-butyl (1R,5S)-3-(2-(4-methyl-1,4-diazepan-1-yl)-5,6,7,8-tetrahydropyrido[3,4-d]pyrimidin-4-yl)-3,8-diazabicyclo[3.2.1]octane-8-carboxylate (**16**)

In a single-necked flask, **15** (130 mg, 0.24 mmol) was dissolved in methanol (10 mL). To the solution was added palladium on carbon (10%, 20 mg). The flask was purged with hydrogen gas three times to remove air and stirred at room temperature under a hydrogen atmosphere for 16 h. The reaction progress was monitored by TLC, indicating completion. The mixture was filtered through a pad of Celite, and the filter cake was washed with methanol (3 × 5 mL). The filtrate was concentrated under reduced pressure to afford crude **16** (white solid), which was used directly in the next step without further purification. 1H NMR (600 MHz, DMSO-d_6_) δ 4.15 (s, 2H), 3.87–3.60 (m, 6H), 3.60–3.15 (m, 5H), 3.07–2.90 (m, 2H), 2.83 (t, *J* = 5.5 Hz, 1H), 2.63 (d, *J* = 18.2 Hz, 1H), 2.60–2.53 (m, 2H), 2.49–2.46 (m, 1H), 2.46–2.38 (m, 2H), 2.30–2.19 (m, 2H), 2.00–1.85 (m, 1H), 1.84–1.80 (m, 2H), 1.79 (s, 3H), 1.41 (s, 9H).

#### 3.2.28. Tert-butyl (1R,5S)-3-(2-(4-methyl-1,4-diazepan-1-yl)-7-(8-methylnaphthalen-1-yl)-5,6,7,8-tetrahydropyrido[3,4-d]pyrimidin-4-yl)-3,8-diazabicyclo[3.2.1]octane-8-carboxylate (**17**)

In a single-necked flask, **16** (100 mg, 0.22 mmol), 1-bromo-8-methylnaphthalene (73 mg, 0.33 mmol), tris(dibenzylideneacetone)dipalladium(0) (18 mg, 0.02 mmol), 4,5-bis(diphenylphosphino)-9,9-dimethylxanthene (XPhos) (12 mg, 0.02 mmol), cesium carbonate (144 mg, 0.44 mmol) and 1,4-dioxane (5 mL) were added sequentially. The air in the flask was replaced with nitrogen, and the reaction proceeded at 100 °C for 16 h before being filtered. The collected solids were rinsed with ethyl acetate (20 mL × 3), while the combined filtrates were concentrated and subjected to silica gel column chromatography (gradient:DCM:MeOH = 15:1 to 8:1) to afford **17** (33 mg, brown solid, 25.19% yield). ^1^H NMR (600 MHz, Methanol-d_4_) δ 8.34–7.82 (m, 1H), 7.73 (m, 1H), 7.61–7.43 (m, 2H), 7.43–7.34 (m, 1H), 7.30–7.17 (m, 1H), 4.30–4.18 (m, 2H), 4.10–3.92 (m, 4H), 3.88–3.70 (m, 3H), 3.68–3.41 (m, 4H), 3.38–3.32 (m, 1H), 3.24 (t, *J* = 5.2 Hz, 1H), 3.11–2.96 (m, 2H), 2.88 (s, 2H), 2.86–2.78 (m, 3H), 2.75–2.69 (m, 1H), 2.62–2.57 (m, 1H), 2.21–2.13 (m, 2H), 2.06–1.70 (m, 4H), 1.48–1.44 (m, 9H), 1.27–1.23 (m, 2H).

#### 3.2.29. 4-((1R,5S)-3,8-Diazabicyclo[3.2.1]octan-3-yl)-2-(4-methyl-1,4-diazepan-1-yl)-7-(8-methylnaphthalen-1-yl)-5,6,7,8-tetrahydropyrido[3,4-d]pyrimidine (**10i**)

The synthesis of compound **10i** followed the procedure for **10b**, yielding **10i** (white solid, 77.23% yield). ^1^H NMR (600 MHz, Methanol-d_4_) δ 8.23 (m, 1H), 7.98 (dd, *J* = 35.3, 7.6 Hz, 1H), 7.89–7.65 (m, 1H), 7.62 (td, *J* = 7.6, 3.6 Hz, 1H), 7.59–7.37 (m, 1H), 7.36–7.12 (m, 1H), 5.12 (s, 1H), 4.52–4.10 (m, 5H), 4.03–3.35 (m, 10H), 3.35–3.30 (m, 4H), 3.27–3.14 (m, 1H), 2.99–2.90 (m, 3H), 2.85–2.57 (m, 1H), 2.57–1.85 (m, 7H). Mp 125.1–128.2 °C. ESI-HRMS *m*/*z* calculated for C_30_H_40_N_7_^+^ 498.3340 [M + H]^+^, found 498.3345 [M + H]^+^.

#### 3.2.30. Tert-butyl (1R,5S)-3-(7-chloro-8-fluoro-2-(4-methyl-1,4-diazepan-1-yl)pyrido[4,3-d]pyrimidin-4-yl)-3,8-diazabicyclo[3.2.1]octane-8-carboxylate (**23**)

In a single-necked flask, tert-butyl 3-(2,7-dichloro-8-fluoropyrido[4,3-d]pyrimidin-4-yl)-3,8-diazabicyclo[3.2.1]octane-8-carboxylate (0.50 g, 1.17 mmol) was suspended in DMF (10 mL). *N*-methylhomopiperazine (267 mg, 2.34 mmol) and DIEA (456 mg, 3.51 mmol) were introduced into the reaction vessel, followed by stirring at 80 °C for 16 h. After completion, the crude product was partitioned between ethyl acetate (100 mL) and water (30 mL). The organic layer underwent three brine washes (30 mL each), was dried over Na_2_SO_4_ and was concentrated in vacuo. Purification via silica gel column chromatography yielded the target compound (gradient: DCM:MeOH = 8:1 to 5:1) to afford compound **23** (440 mg, white solid, 74.58% yield). ^1^H NMR (600 MHz, Chloroform-d) δ 8.51 (s, 1H), 4.45–4.21 (m, 4H), 4.17–3.78 (m, 4H), 3.65–3.41 (m, 2H), 2.93–2.61 (m, 4H), 2.50–2.38 (m, 3H), 2.18–2.06 (m, 2H), 1.99–1.89 (m, 2H), 1.78–1.69 (m, 2H), 1.50 (s, 9H). ^13^C NMR (151 MHz, Chloroform-d) δ 163.9, 163.8, 159.6, 153.3, 150.5, 150.5, 148.9, 147.1, 143.6, 143.5, 137.2, 137.1, 137.1, 109.1, 80.2, 58.2, 58.0, 57.2, 57.0, 54.0, 53.3, 46.6, 46.3, 46.2, 28.4, 27.2, 26.5.

#### 3.2.31. Tert-butyl (1R,5S)-3-(8-fluoro-7-(7-fluoro-3-(methoxymethoxy)-8-((triisopropylsilyl)ethynyl)naphthalen-1-yl)-2-(4-methyl-1,4-diazepan-1-yl)pyrido[4,3-d]pyrimidin-4-yl)-3,8-diazabicyclo[3.2.1]octane-8-carboxylate (**24**)

In a single-necked flask, **23** (400 mg, 0.79 mmol), ((2-fluoro-6-(methoxymethoxy)-8-(4,4,5,5-tetramethyl-1,3,2-dioxaborolan-2-yl)naphthalen-1-yl)ethynyl)triisopropylsilane (609 mg, 1.19 mmol), K_3_PO_4_ (502 mg, 2.37 mmol), Ad_2_nBuP-Pd-G3 (58 mg, 0.08 mmol) and 1,4-dioxane (5 mL) were combined. Under a nitrogen atmosphere, the reaction mixture was maintained at 80 °C with continuous stirring for 5 h. The reaction was diluted with ethyl acetate (30 mL), and the solid was filtered under reduced pressure. The collected solids were rinsed with ethyl acetate (3 × 10 mL), and the combined washes were concentrated. The residue was purified via silica gel column chromatography (gradient: DCM:MeOH = 10:1 to 7:1) to afford **24** (430 mg, white solid, 63.70% yield). ^1^H NMR (600 MHz, Chloroform-d) δ 8.89 (s, 1H), 7.77 (dd, *J* = 9.0, 5.6 Hz, 1H), 7.48 (d, *J* = 2.6 Hz, 1H), 7.32–7.29 (m, 1H), 7.29–7.28 (m, 1H), 7.28–7.27 (m, 1H), 7.24 (s, 1H), 5.33–5.24 (m, 3H), 4.69–3.93 (m, 10H), 2.73–2.52 (m, 4H), 2.02–2.01 (m, 2H), 2.00 (s, 3H), 1.76–1.72 (m, 4H), 1.51 (s, 9H), 1.25–1.24 (m, 3H), 0.87 (dd, *J* = 15.8, 7.4 Hz, 18H).

#### 3.2.32. 4-(4-((1R,5S)-3,8-Diazabicyclo[3.2.1]octan-3-yl)-8-fluoro-2-(4-methyl-1,4-diazepan-1-yl)pyrido[4,3-d]pyrimidin-7-yl)-5-ethynyl-6-fluoronaphthalen-2-ol (**10k**)

In a single-necked flask, **24** (100 mg, 0.12 mmol) was suspended in DMF (5 mL). Following the addition of CsF (89 mg, 0.58 mmol), the reaction was maintained at 50 °C in an oil bath for 2 h, monitored by TLC until complete conversion to the desilylated intermediate occurred. After triturating the reaction mixture in water (20 mL), the resulting solid was collected by filtration and subjected to three distilled water washes (5 mL each), affording **25**. **25** was redissolved in dichloromethane (10 mL), and the solution was cooled to 0 °C in an ice bath. A solution of HCl in 1,4-dioxane (4 mol/L, 1.2 mL) was introduced dropwise. After full addition, the reaction temperature was gradually raised to an ambient temperature and maintained with stirring for 5 h. The mixture was concentrated under reduced pressure, adjusted to pH 8–9 with saturated sodium bicarbonate and processed with ethyl acetate (50 mL)-water (10 mL) biphasic extraction. After brine (10 mL × 3) washing (saturated) and drying (Na_2_SO_4_), the organic extract was concentrated, with the resulting residue chromatographed on silica gel (gradient: DCM:MeOH = 10:1 to 7:1) to afford **10k** (31 mg, pale yellow solid, 47.23% yield). ^1^H NMR (600 MHz, Methanol-d4) δ 8.75 (s, 1H), 7.87–7.81 (m, 1H), 7.35–7.27 (m, 2H), 7.18 (t, *J* = 2.7 Hz, 1H), 4.50 (d, *J* = 12.9 Hz, 1H), 4.44–4.34 (m, 1H), 4.04 (d, *J* = 14.1 Hz, 2H), 3.97 (s, 2H), 3.69 (s, 2H), 3.61 (dd, *J* = 25.7, 12.9 Hz, 3H), 2.93–2.80 (m, 2H), 2.75–2.63 (m, 2H), 2.44 (s, 3H), 2.11–2.03 (m, 2H), 1.96–1.82 (m, 5H). Mp 198.2–201.3 °C. ESI-HRMS *m*/*z* calculated for C_31_H_32_F_2_N_7_O^+^ 556.2631 [M + H]^+^, found 556.2628 [M + H]^+^.

#### 3.2.33. Tert-butyl 4-(2-(2-((2-(2,6-dioxopiperidin-3-yl)-1,3-dioxoisoindolin-4-yl)amino)ethoxy)ethyl)-1,4-diazepane-1-carboxylate (**28**)

In a single-necked flask, 1,4-diazepane-1-carboxylic acid tert-butyl ester (1.00 g, 5.00 mmol), **27** (2.58 g, 5.00 mmol), DMSO (10 mL) and DIEA (1.3 g, 10.00 mmol) were added sequentially. Under a nitrogen purge, the reaction was maintained at 70 °C with continuous stirring for 4 h, with reaction progress monitored by TLC until completion. The reaction mixture was extracted with ethyl acetate (100 mL)/water (50 mL). The organic extract underwent brine (50 mL × 3) washing, Na_2_SO_4_ drying and vacuum concentration. Purification by column chromatography afforded product **28** (2.12 g, yellow solid, 78.08% yield). ^1^H NMR (400 MHz, Chloroform-d) δ 7.48 (dd, *J* = 8.5, 7.1 Hz, 1H), 7.09 (d, *J* = 7.1 Hz, 1H), 6.89 (d, *J* = 8.5 Hz, 1H), 6.53–6.43 (m, 1H), 4.96–4.83 (m, 1H), 3.66 (t, *J* = 5.5 Hz, 2H), 3.59 (t, *J* = 5.5 Hz, 2H), 3.50 (t, *J* = 5.5 Hz, 2H), 3.46–3.43 (m, 4H), 3.39 (t, *J* = 6.3 Hz, 2H), 3.00–2.95 (m, 1H), 2.93–2.90 (m, 1H), 2.87–2.81 (m, 1H), 2.77–2.75 (m, 2H), 2.70–2.66 (m, 2H), 2.15–2.08 (m, 1H), 1.93–1.83 (m, 2H), 1.44 (s, 9H).

#### 3.2.34. 4-((2-(2-(1,4-Diazepan-1-yl)ethoxy)ethyl)amino)-2-(2,6-dioxopiperidin-3-yl)isoindoline-1,3-dione (**29**)

In a single-necked flask, **28** (2.0 g, 3.68 mmol) was dissolved in DCM (10 mL). We added TFA (5 mL) dropwise to the stirring solution and continued stirring at an ambient temperature for 1 h. Reaction completion was confirmed by TLC monitoring. The DCM and trifluoroacetic acid were evaporated under reduced pressure. The residue was adjusted to alkaline pH with saturated sodium bicarbonate solution and extracted with dichloromethane (100 mL)/water (50 mL). The organic layer underwent brine (50 mL × 3) washing, dehydration with anhydrous Na_2_SO_4_ and solvent evaporation to afford the crude product **29** (2.40 g), which was directly used in the next step.

#### 3.2.35. (2S,4R)-1-((S)-2-(6-Bromohexanamido)-3,3-dimethylbutanoyl)-4-hydroxy-*N*-((S)-1-(4-(4-methylthiazol-5-yl)phenyl)ethyl)pyrrolidine-2-carboxamide (**31**)

In a single-necked flask, 6-bromohexanoic acid (300 mg, 1.44 mmol) was dissolved in DMF. The mixture was cooled to 0 °C using an ice-salt bath. While stirring, HATU (657 mg, 1.73 mmol) and DIEA (281 mg, 2.16 mmol) were slowly added sequentially. After complete addition, the reaction was maintained at this temperature for 5 min. Following gradual addition of the VHL ligand (700 mg, 1.44 mmol), the reaction was warmed to an ambient temperature and stirred for 12 h. The crude product was then partitioned between ethyl acetate (50 mL) and water (30 mL). The organic extract underwent brine washing (20 mL × 3), Na_2_SO_4_ drying and vacuum concentration. Purification by column chromatography afforded product **31** (680 mg, off-white solid, 76.23% yield). ^1^H NMR (600 MHz, Chloroform-d) δ 8.67 (s, 1H), 7.43 (d, *J* = 7.9 Hz, 1H), 7.38 (d, *J* = 8.1 Hz, 2H), 7.35 (d, *J* = 8.1 Hz, 2H), 6.28 (d, *J* = 8.8 Hz, 1H), 5.06 (t, *J* = 7.2 Hz, 1H), 4.67 (t, *J* = 7.8 Hz, 1H), 4.56 (d, *J* = 8.8 Hz, 1H), 4.49 (qd, *J* = 4.6, 3.2, 2.4 Hz, 1H), 4.01 (d, *J* = 11.2 Hz, 1H), 3.61 (dd, *J* = 11.2, 4.0 Hz, 1H), 3.41–3.30 (m, 2H), 2.50 (d, *J* = 1.0 Hz, 3H), 2.44 (ddd, *J* = 12.8, 7.5, 4.9 Hz, 1H), 2.17 (t, *J* = 7.5 Hz, 2H), 2.09–1.98 (m, 1H), 1.82 (p, *J* = 7.1 Hz, 2H), 1.60 (q, *J* = 7.6 Hz, 2H), 1.46 (d, *J* = 7.0 Hz, 3H), 1.42 (dt, *J* = 12.3, 4.4 Hz, 2H), 1.02 (s, 9H). ^13^C NMR (151 MHz, Chloroform-d) δ 173.1, 171.9, 169.7, 162.5, 150.3, 148.3, 143.1, 131.5, 130.7, 129.4, 126.4, 69.8, 58.5, 57.4, 56.5, 48.7, 36.4, 36.0, 35.5, 35.1, 33.4, 32.2, 31.3, 27.6, 26.4, 24.5, 22.1, 15.9.

#### 3.2.36. Tert-butyl 4-(6-(((S)-1-((2S,4R)-4-hydroxy-2-(((S)-1-(4-(4-methylthiazol-5-yl)phenyl)ethyl)carbamoyl)pyrrolidin-1-yl)-3,3-dimethyl-1-oxobutan-2-yl)amino)-6-oxohexyl)-1,4-diazepane-1-carboxylate (**32**)

Compound **32** was obtained in a 71.24% yield as an off-white solid, using **31** as the starting material and employing the same procedure as that for compound **28**. ^1^H NMR (600 MHz, Chloroform-d) δ 8.65 (s, 1H), 7.44 (dd, *J* = 25.3, 7.8 Hz, 1H), 7.36 (q, *J* = 7.8 Hz, 4H), 6.28–6.19 (m, 1H), 5.27 (s, 1H), 5.06 (m, 1H), 4.68 (t, *J* = 8.0 Hz, 1H), 4.57 (d, *J* = 8.4 Hz, 1H), 3.99 (d, *J* = 10.5 Hz, 1H), 3.60 (d, *J* = 10.5 Hz, 1H), 3.51–3.45 (m, 2H), 3.43–3.35 (m, 4H), 2.89–2.82 (m, 2H), 2.60–2.55 (m, 2H), 2.50 (s, 3H), 2.47–2.43 (m, 1H), 2.23–2.10 (m, 2H), 2.07–2.00 (m, 1H), 1.87–1.72 (m, 4H), 1.61–1.57 (m, 2H), 1.47–1.45 (m, 2H), 1.44 (s, 9H), 1.40 (s, 3H), 1.02 (s, 9H). ^13^C NMR (151 MHz, Chloroform-d) δ 173.5, 172.2, 169.9, 169.8, 155.6, 150.4, 148.6, 143.3, 131.6, 131.0, 129.6, 126.5, 79.4, 69.9, 58.7, 57.5, 56.8, 55.9, 54.6, 48.9, 45.9, 45.1, 36.4, 35.7, 35.3, 28.6, 27.1, 27.0, 26.6, 22.7, 22.3, 16.1, 14.2.

#### 3.2.37. Tert-butyl 4-(6-(((S)-1-((2S,4R)-4-hydroxy-2-(((S)-1-(4-(4-methylthiazol-5-yl)phenyl)ethyl)carbamoyl)pyrrolidin-1-yl)-3,3-dimethyl-1-oxobutan-2-yl)amino)-6-oxohexyl)-1,4-diazepane-1-carboxylate (**33**)

Compound **33** was synthesized following the procedure described for compound **29**, using **32** as the starting material. The crude product **33** was obtained and directly used in the next step without further purification.^1^H NMR (600 MHz, Chloroform-d) δ 8.66 (d, *J* = 1.1 Hz, 1H), 7.43 (d, *J* = 7.8 Hz, 1H), 7.39–7.37 (m, 2H), 7.36–7.33 (m, 2H), 6.31 (d, *J* = 8.8 Hz, 1H), 5.07 (t, *J* = 7.2 Hz, 2H), 4.67 (t, *J* = 8.0 Hz, 1H), 4.59 (d, *J* = 8.8 Hz, 1H), 4.49 (s, 1H), 3.99 (d, *J* = 11.0 Hz, 1H), 3.60 (dd, *J* = 11.0, 3.7 Hz, 1H), 3.13–2.99 (m, 4H), 2.72 (q, *J* = 6.3, 5.8 Hz, 2H), 2.70–2.61 (m, 2H), 2.55–2.50 (m, 4H), 2.50–2.47 (m, 2H), 2.40 (m, 1H), 2.26–2.15 (m, 2H), 2.12–2.05 (m, 1H), 1.84 (t, *J* = 6.0 Hz, 2H), 1.61 (m, 2H), 1.49–1.45 (m, 4H), 1.44 (t, *J* = 7.0 Hz, 2H), 1.33 (dt, *J* = 14.3, 7.0 Hz, 2H), 1.02 (s, 9H). ^13^C NMR (151 MHz, Chloroform-d) δ 173.4, 171.7, 169.9, 150.2, 148.4, 143.2, 131.5, 130.8, 129.5, 126.4, 126.3, 69.7, 58.7, 57.5, 57.4, 57.0, 54.5, 54.1, 48.7, 46.8, 45.8, 35.8, 35.3, 28.0, 26.4, 24.5, 22.1, 16.0.

#### 3.2.38. Tert-butyl (1R,5S)-3-(7-chloro-2-(4-(2-(2-((2-(2,6-dioxopiperidin-3-yl)-1,3-dioxoisoindolin-4-yl)amino)ethoxy)ethyl)-1,4-diazepan-1-yl)-8-fluoropyrido[4,3-d]pyrimidin-4-yl)-3,8-diazabicyclo[3.2.1]octane-8-carboxylate (**23a**)

Compound **23a** was prepared from starting material **29** using the method applied for compound **23**, yielding a yellow solid with a 52.78% yield. ^1^H NMR (600 MHz, Chloroform-d) δ 8.50 (s, 1H), 7.57–7.45 (m, 1H), 7.11 (d, *J* = 7.1 Hz, 1H), 6.90 (t, *J* = 9.1 Hz, 1H), 6.47 (d, *J* = 6.6 Hz, 1H), 4.90 (dd, *J* = 12.1, 5.4 Hz, 1H), 4.46–4.20 (m, 4H), 4.12–3.76 (m, 4H), 3.71–3.66 (m, 2H), 3.66–3.62 (m, 1H), 3.60–3.48 (m, 2H), 3.44 (t, *J* = 5.4 Hz, 2H), 3.09–2.59 (m, 8H), 2.16–1.98 (m, 2H), 1.95–1.89 (m, 2H), 1.78–1.71 (m, 2H), 1.69–1.58 (m, 3H), 1.50 (s, 9H). ^13^C NMR (151 MHz, Chloroform-d) δ 171.1, 168.4, 159.6, 153.5, 143.8, 136.2, 132.7, 116.9, 111.9, 110.4, 109.3, 80.5, 69.5, 69.4, 55.5, 54.9, 53.5, 49.0, 46.3, 42.4, 31.5, 28.6, 22.9.

#### 3.2.39. Tert-butyl (1R,5S)-3-(2-(4-(2-(2-((2-(2,6-dioxopiperidin-3-yl)-1,3-dioxoisoindolin-4-yl)amino)ethoxy)ethyl)-1,4-diazepan-1-yl)-8-fluoro-7-(7-fluoro-3-(methoxymethoxy)-8-((triisopropylsilyl)ethynyl)naphthalen-1-yl)pyrido[4,3-d]pyrimidin-4-yl)-3,8-diazabicyclo[3.2.1]octane-8-carboxylate (**24a**)

Compound **24a** was prepared from **23a** as the starting material, using the method outlined for compound **24**, resulting in a yellow solid with a yield of 42.38%. ^1^H NMR (600 MHz, DMSO-d_6_) δ 11.09 (s, 1H), 8.86 (s, 1H), 8.07 (dd, *J* = 9.1, 5.7 Hz, 1H), 7.76–7.67 (m, 1H), 7.54 (q, *J* = 10.4, 9.1 Hz, 2H), 7.29 (s, 1H), 7.13 (t, *J* = 8.4 Hz, 1H), 7.02 (t, *J* = 9.1 Hz, 1H), 6.60 (s, 1H), 5.35 (s, 2H), 5.11–4.97 (m, 1H), 4.63–4.52 (m, 1H), 4.27 (d, *J* = 26.5 Hz, 2H), 4.15–4.01 (m, 2H), 3.98–3.88 (m, 5H), 3.89–3.77 (m, 3H), 3.60–3.58 (m, 1H), 3.54 (t, *J* = 5.9 Hz, 2H), 3.49–3.44 (m, 2H), 3.31–3.29 (m, 1H), 3.28–3.24 (m, 1H), 3.19–3.15 (m, 3H), 2.91–2.83 (m, 1H), 2.82–2.73 (m, 1H), 2.72–2.59 (m, 4H), 2.02–1.97 (m, 1H), 1.91–1.87 (m, 2H), 1.72–1.60 (m, 2H), 1.47–1.43 (m, 9H), 1.24–1.21 (m, 3H), 1.07–1.06 (m, 18H).

#### 3.2.40. 4-((2-(2-(4-(4-((1R,5S)-3,8-Diazabicyclo[3.2.1]octan-3-yl)-7-(8-ethynyl-7-fluoro-3-hydroxynaphthalen-1-yl)-8-fluoropyrido[4,3-d]pyrimidin-2-yl)-1,4-diazepan-1-yl)ethoxy)ethyl)amino)-2-(2,6-dioxopiperidin-3-yl)isoindoline-1,3-dione (**26a**)

Compound **26a** was synthesized following the procedure described for compound **10k**, using **24a** as the starting material. The target compound **26a** was obtained as a yellow solid with a yield of 64.25%. ^1^H NMR (400 MHz, DMSO-d_6_) δ 11.09 (s, 1H), 7.61–7.54 (m, 1H), 7.14 (d, *J* = 8.5 Hz, 1H), 7.04 (d, *J* = 7.0 Hz, 1H), 6.60 (s, 1H), 5.05 (dd, *J* = 12.9, 5.3 Hz, 1H), 3.62 (t, *J* = 5.4 Hz, 2H), 3.59–3.55 (m, 2H), 3.55–3.52 (m, 2H), 3.52–3.44 (m, 8H), 3.42–3.36 (m, 2H), 2.99–2.79 (m, 1H), 2.65–2.51 (m, 2H), 2.12–1.94 (m, 1H). Mp 127.2–130.4 °C. ESI-HRMS *m*/*z* calculated for C_47_H_47_F_2_N_10_O_6_^+^ 885.3643 [M + H]^+^, found 885.3638 [M + H]^+^.

#### 3.2.41. Tert-butyl (1R,5S)-3-(7-chloro-8-fluoro-2-(4-(6-(((S)-1-((2S,4R)-4-hydroxy-2-(((S)-1-(4-(4-methylthiazol-5-yl)phenyl)ethyl)carbamoyl)pyrrolidin-1-yl)-3,3-dimethyl-1-oxobutan-2-yl)amino)-6-oxohexyl)-1,4-diazepan-1-yl)pyrido[4,3-d]pyrimidin-4-yl)-3,8-diazabicyclo[3.2.1]octane-8-carboxylate (**23b**)

Compound **23b** was obtained using **33** as the starting material and the method outlined for compound **23**, yielding a pale yellow solid at 27.20%.

#### 3.2.42. Tert-butyl (1R,5S)-3-(8-fluoro-7-(7-fluoro-3-(methoxymethoxy)-8-((triisopropylsilyl)ethynyl)naphthalen-1-yl)-2-(4-(6-(((S)-1-((2S,4R)-4-hydroxy-2-(((S)-1-(4-(4-methylthiazol-5-yl)phenyl)ethyl)carbamoyl)pyrrolidin-1-yl)-3,3-dimethyl-1-oxobutan-2-yl)amino)-6-oxohexyl)-1,4-diazepan-1-yl)pyrido[4,3-d]pyrimidin-4-yl)-3,8-diazabicyclo[3.2.1]octane-8-carboxylate (**24b**)

Compound **24b** was prepared using the method outlined for compound **24a**, with **23b** as the starting material, yielding a white solid at 43.62%.

#### 3.2.43. (2S,4R)-1-((S)-2-(6-(4-(4-((1R,5S)-3,8-Diazabicyclo[3.2.1]octan-3-yl)-7-(8-ethynyl-7-fluoro-3-hydroxynaphthalen-1-yl)-8-fluoropyrido[4,3-d]pyrimidin-2-yl)-1,4-diazepan-1-yl)hexanamido)-3,3-dimethylbutanoyl)-4-hydroxy-*N*-((S)-1-(4-(4-methylthiazol-5-yl)phenyl)ethyl)pyrrolidine-2-carboxamide (**26b**)

Compound **26b** was synthesized following the procedure described for compound **10k**, using **24b** as the starting material, yielding a white solid at 34.65%. Mp 116.3–118.7 °C. ESI-HRMS *m*/*z* calculated for C_59_H_70_F_2_N_11_O_5_S^+^ 1082.5245 [M + H]^+^, found 1082.5252 [M + H]^+^.

### 3.3. KRAS-G12D Enzyme Inhibition Assay

The KRAS-G12D enzyme inhibition assay was performed following literature-reported procedures [8,10].

### 3.4. Cell Proliferation Assay

The cell proliferation assay was performed following literature-reported procedures [8,10].

### 3.5. Molecular Docking

The crystal structure of KRAS-G12D (PDB 7RPZ) from the Protein Data Bank was utilized for docking studies. Prior to docking, both the protein structure and docking molecules were optimized using Sybyl X V2.0 software, followed by docking studies performed with the Surflex-Dock GeomX module of the same software.

## 4. Conclusions

In order to explore new classes of small-molecule inhibitors and targeted protein degraders (PROTACs) targeting KRAS-G12D, eleven novel inhibitors featuring a seven-membered ring pharmacophore were successfully synthesized. Through evaluations at the molecular and cellular levels, it was found that compound **10c** exhibited potent anti-proliferative activity, while **10k** showed strong enzymatic inhibitory activity (IC50 0.009 μM). Using **10k** as the warhead, a linker was introduced at the *N*-methyl position of the seven-membered ring. Two PROTACs were subsequently designed and synthesized by conjugating either pomalidomide or VHL ligands as E3 ubiquitin ligase recruiters. When tested in the human gastric cancer AGS cell line and human pancreatic cancer ASPC-1 cell line carrying the KRAS-G12D mutation, the two PROTACs did not exhibit significant advantages over the inhibitor **10k** and the positive control drug MRTX1133. It is speculated that this may be due to the poor cell penetration ability of the PROTACs or the type and length of the linker, which may have a certain inhibitory effect on the formation of the ternary complex. Nevertheless, this study is the first to use a seven-membered ring as a pharmacophore and as a transition group between the warhead and the linker, providing valuable insights for the development of new therapeutic strategies targeting the KRAS family.

## Figures and Tables

**Table 1:** Cell proliferation experimental results of the inhibitors **10a**–**10k**.

Structure	ID	IC_50_ (μM)			
		AGS (G12D)	Panc1 (G12D)	HCT116 (G13D)	A549 (WT)
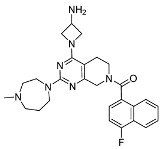	**10a**	ND ^a^	6.17 ± 0.20 **	12.60 ± 1.01 ^ns^	>20
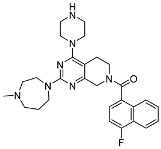	**10b**	ND	5.87 ± 0.37 ***	11.24 ± 0.94 ^ns^	7.89 ± 0.17 **
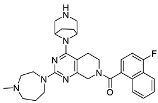	**10c**	3.40 ± 0.25 ***	1.40 ± 0.19 *	5.13 ± 0.40 ***	6.88 ± 0.19 ***
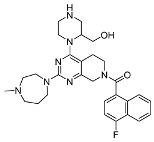	**10d**	ND	>20	>20	7.48 ± 0.19 **
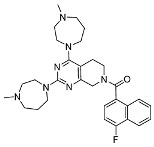	**10f**	ND	3.48 ± 0.22 **	10.12 ± 0.31 **	>20
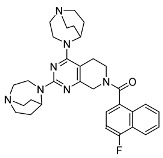	**10g**	ND	5.10 ± 0.32 **	7.12 ± 0.45 **	12.86 ± 0.38 ^ns^
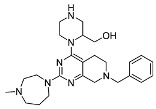	**10h**	ND	>20	>20	>20
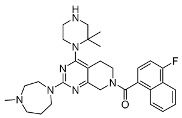	**10e**	ND	>20	11.88 ± 1.59 ^ns^	12.79 ± 1.84 ^ns^
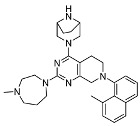	**10i**	ND	3.92 ± 0.58 **	4.51 ± 0.21 ***	5.50 ± 1.35 ***
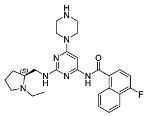	**10j**	ND	5.96 ± 0.64 ***	14.47 ± 1.22 ^ns^	>20
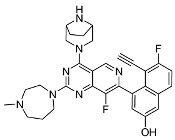	**10k**	2.22 ± 0.53 **	11.49 ± 0.41 ***	7.87 ± 0.16 ***	14.20 ± 2.15 ***
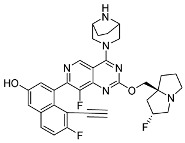	MRTX1133	1.46 ± 0.18	0.39 ± 0.05	12.62 ± 0.47	12.74 ± 0.98

^a^ Not determined; * *p* < 0.05, ** *p* < 0.01, *** *p* < 0.001 compared to MRTX1133; ^ns^ indicates no statistical significance (*p* > 0.05).

**Table 2:** The antiproliferative activity of a novel PROTAC targeting KRAS-G12D.

Structure	ID	IC_50_ (μM)	
		AGS	ASPC-1
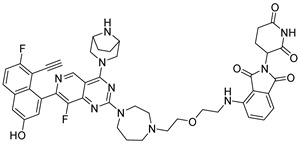	**26a**	~3.00	>10.00
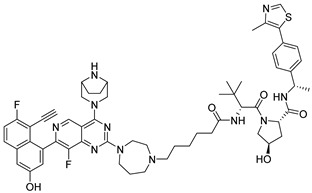	**26b**	ND ^a^	5.09 ± 0.89
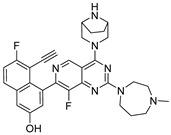	**10k**	2.22 ± 0.53 *	ND
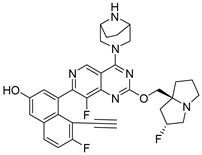	MRTX1133	1.46 ± 0.18	<0.10

^a^ Not determined; * *p* < 0.05.

## Data Availability

Data are contained within this article and Supplementary Materials.

## Supplementary Materials

The following supporting information can be downloaded at: https://www.mdpi.com/article/10.3390/ph18050696/s1, Figure S1: ^1^H NMR spectrum of **8b**; Figure S2: ^13^C NMR spectrum of **8b**; Figure S3: ^1^H NMR spectrum of **9b**; Figure S4: ^13^C NMR spectrum of **9b**; Figure S5: ^1^H NMR spectrum of **10b**; Figure S6: ^13^C NMR spectrum of **10b**; Figure S7: HR-MS chromatogram spectrum of **10b**; Figure S8: ^1^H NMR spectrum of **8d**; Figure S9: ^1^H NMR spectrum of **9d**; Figure S10: ^13^C NMR spectrum of **9d**; Figure S11: ^1^H NMR spectrum of **10d**; Figure S12: ^13^C NMR spectrum of **10d** Figure S13: HR-MS chromatogram spectrum of **10d**; Figure S14: ^1^H NMR spectrum of **19**; Figure S15: ^13^C NMR spectrum of **19**; Figure S16: ^1^H NMR spectrum of **20**; Figure S17: ^13^C NMR spectrum of **20**; Figure S18: ^1^H NMR spectrum of **21**; Figure S19: ^13^C NMR spectrum of **21**; Figure S20: ^1^H NMR spectrum of **10j**; Figure S21: HR-MS chromatogram spectrum of **10j**; Figure S22: ^1^H NMR spectrum of **12**; Figure S23: ^13^C NMR spectrum of **12**; Figure S24: ^1^H NMR spectrum of **13**; Figure S25: ^13^C NMR spectrum of **13**; Figure S26: ^1^H NMR spectrum of **10h**; Figure S27: ^13^C NMR spectrum of **10h**; Figure S28: HR-MS chromatogram spectrum of **10h**; Figure S29: ^1^H NMR spectrum of **10f**; Figure S30: HR-MS chromatogram spectrum of **10f**; Figure S31: ^1^H NMR spectrum of **10a** Figure S32: HR-MS chromatogram spectrum of **10a**; Figure S33: ^1^H NMR spectrum of **8c**; Figure S34: ^13^C NMR spectrum of **8c**; Figure S35: ^1^H NMR spectrum of **10c**; Figure S36: HR-MS chromatogram spectrum of **10c**; Figure S37: ^1^H NMR spectrum of **23**; Figure S38: ^13^C NMR spectrum of **23**; Figure S39: ^1^H NMR spectrum of **24**; Figure S40: ^1^H NMR spectrum of **10K**; Figure S41: HR-MS chromatogram spectrum of **10k**; Figure S42: ^1^H NMR spectrum of **15**; Figure S43: ^1^H NMR spectrum of **16**; Figure S44: ^1^H NMR spectrum of **11**; Figure S45: ^1^H NMR spectrum of **10i**; Figure S46: HR-MS chromatogram spectrum of **10i**; Figure S47: ^1^H NMR spectrum of **28**; Figure S48: ^1^H NMR spectrum of **23a**; Figure S49: ^13^C NMR spectrum of **23a**; Figure S50: ^1^H NMR spectrum of **24a**; Figure S51: HR-MS chromatogram spectrum of **26a**; Figure S52: ^1^H NMR spectrum of **31**; Figure S53: ^13^C NMR spectrum of **31**; Figure S54: ^1^H NMR spectrum of **32**; Figure S55: ^13^C NMR spectrum of **32**; Figure S56: ^1^H NMR spectrum of **33**; Figure S57: ^13^C NMR spectrum of **33**; Figure S58: ^1^H NMR spectrum of **23b**; Figure S59: ^13^C NMR spectrum of **23b**; Figure S60: HR-MS chromatogram spectrum of **26b**; Table S1: KRAS-G12D-inhibitory activity of the inhibitors.
