# Supplementary material for: Design, Synthesis, Anticancer Evaluation and Molecular Docking of Pyrimidine, Pyrido[4,3-d]pyrimidine and 5,6,7,8-Tetrahydropyrido[3,4-d]pyrimidine Derivatives as Novel KRAS-G12D Inhibitors and PROTACs
Source: Pharmaceuticals (Basel). 2025 May 8;18(5):696. doi: 10.3390/ph18050696 (PMC12114952; doi:10.3390/ph18050696)
Supplement: Supplementary file 1 [file pharmaceuticals-18-00696-s001.zip › pharmaceuticals-3586599-supplementary.pdf]

Supplementary Data

Design, Synthesis, Anticancer Evaluation and Molecular Docking of Pyrimidine, Pyrido[4,3-d]pyrimidine and 5,6,7,8-Tetrahydropyrido[3,4-d]pyrimidine Derivatives as Novel KRAS-G12D Inhibitors and PROTACs

Hailong Yang, Lu Gan and Huabei Zhang *

Key Laboratory of Radiopharmaceuticals of the Ministry of Education, College of Chemistry, Beijing Normal University, Beijing 100875, China; huaxuehailong@163.com (H.Y.); 202221150066@mail.bnu.edu.cn (L.G.)
* Correspondence: hbzhang@bnu.edu.cn

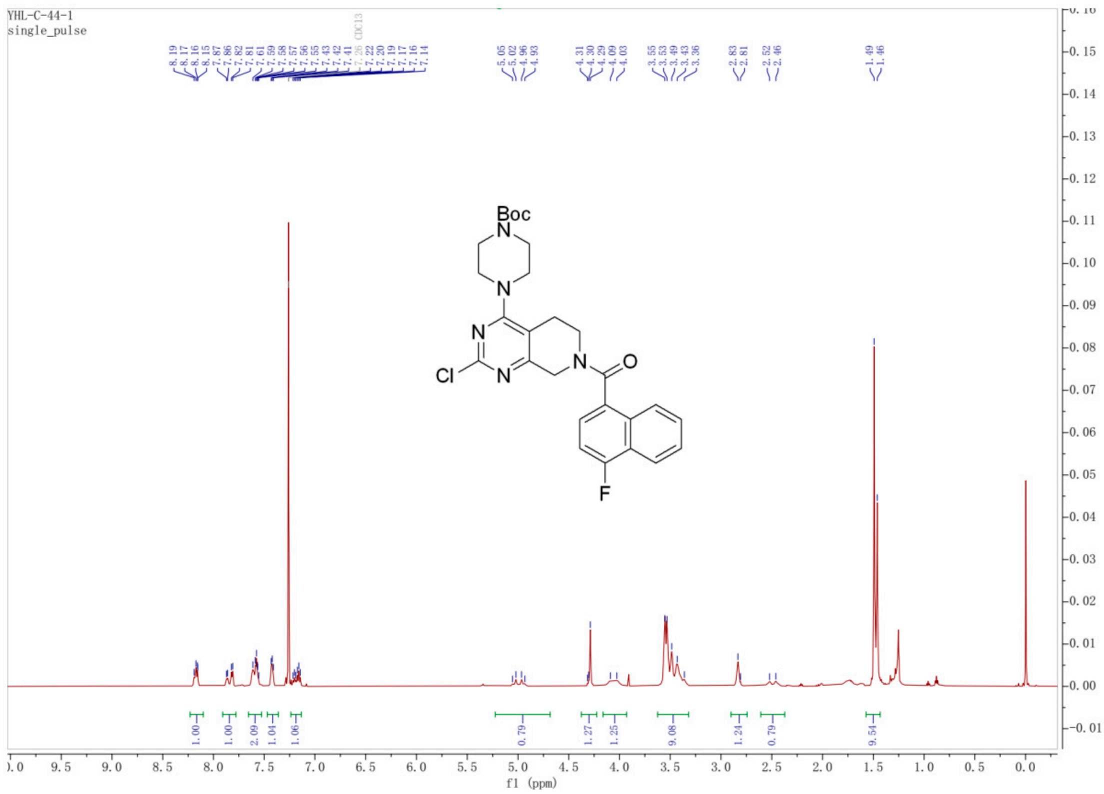

Figure S1. ^{1}H NMR spectrum of **8b**.

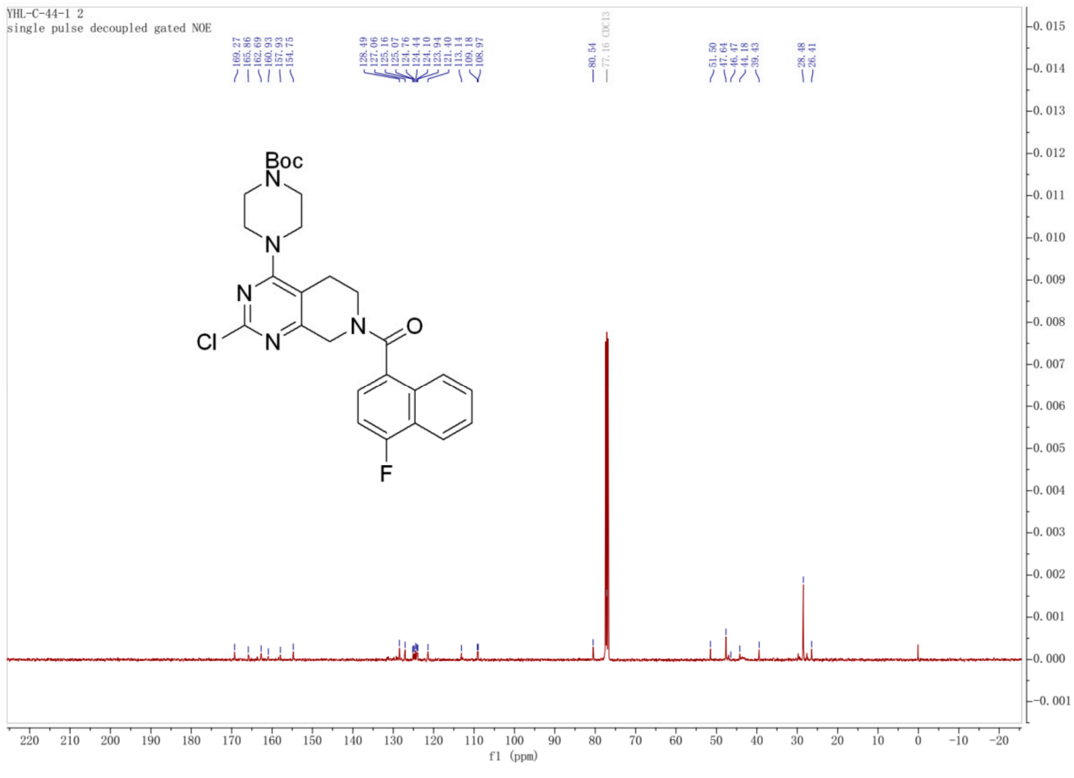

Figure S2. ^{13}C NMR spectrum of **8b**.

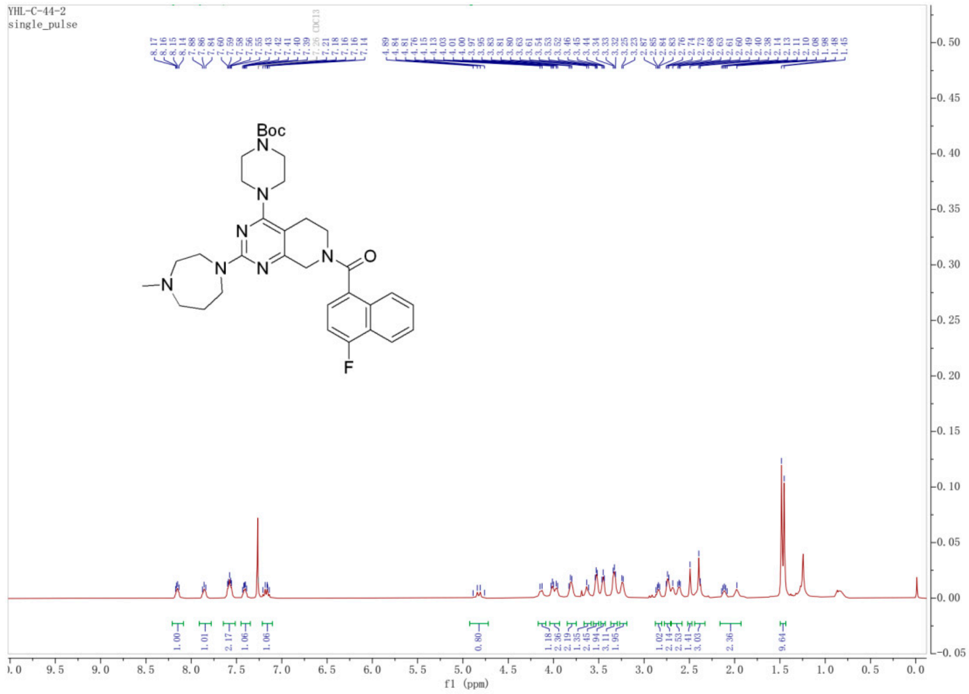

Figure S3. ^{1}H NMR spectrum of **9b**.

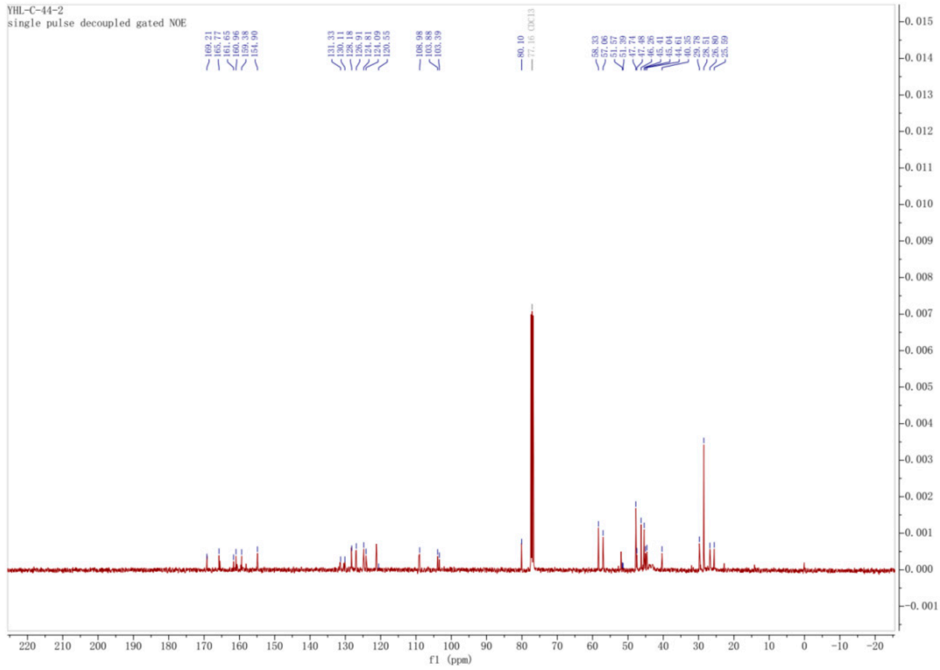

Figure S4. ^{13}C NMR spectrum of **9b**.

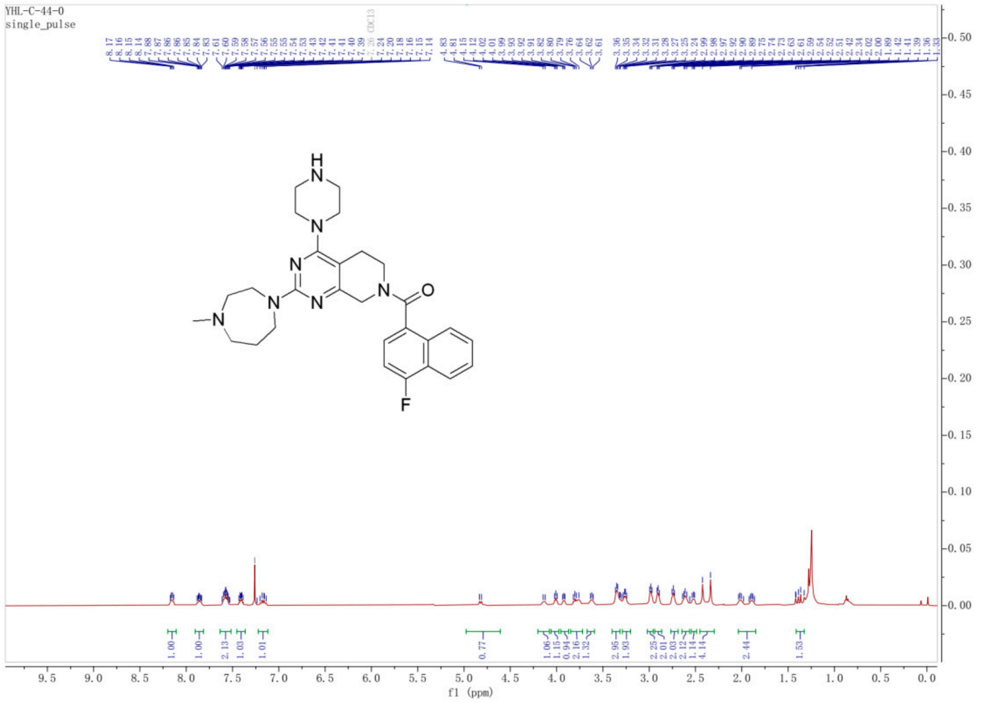

Figure S5. ^{1}H NMR spectrum of **10b**.

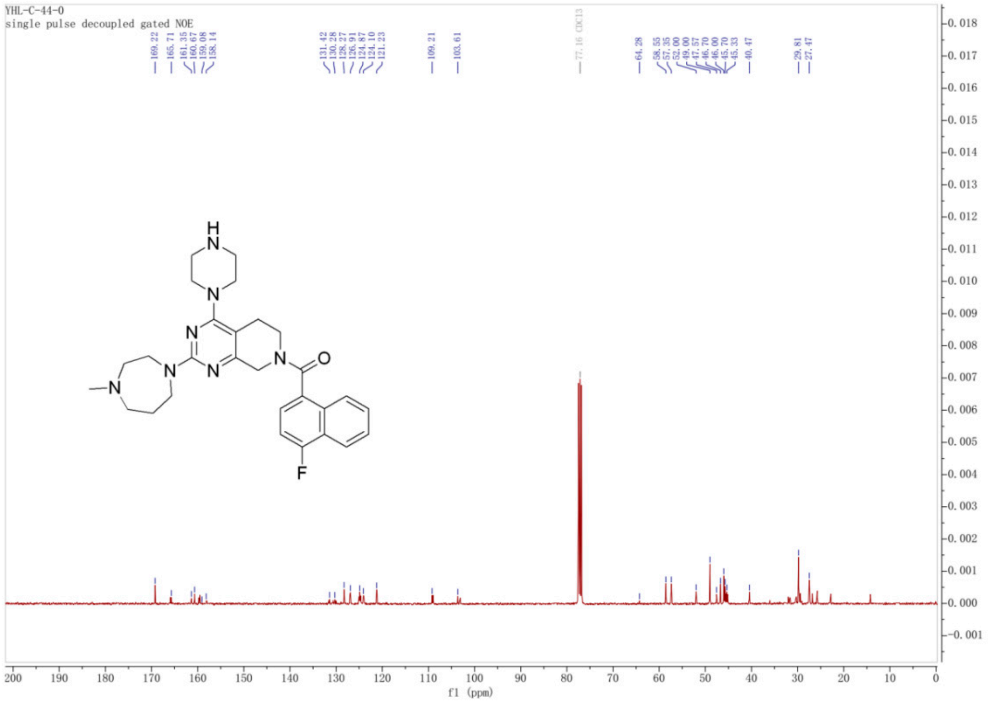

Figure S6. ^{13}C NMR spectrum of **10b**.

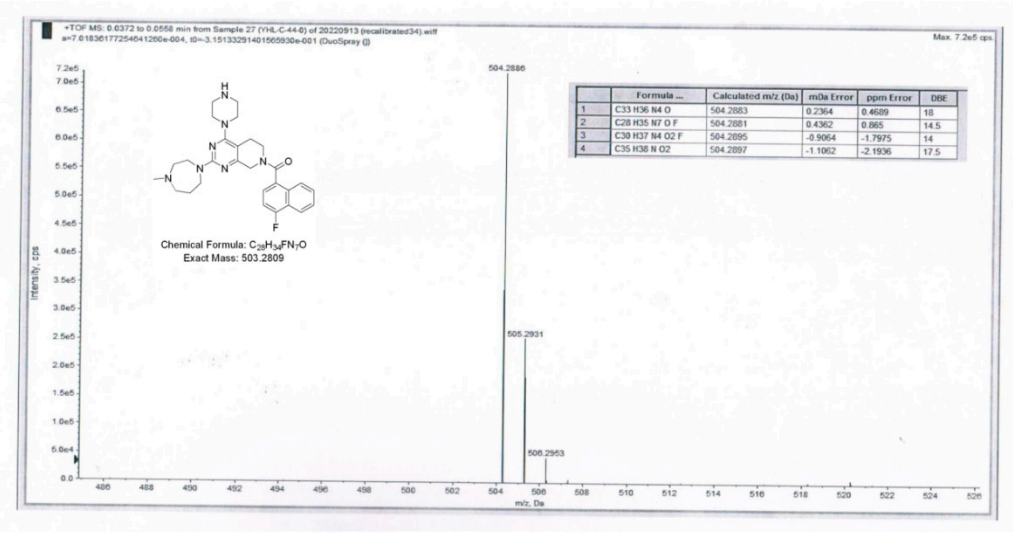

Figure S7. HR-MS chromatogram spectrum of **10b**.

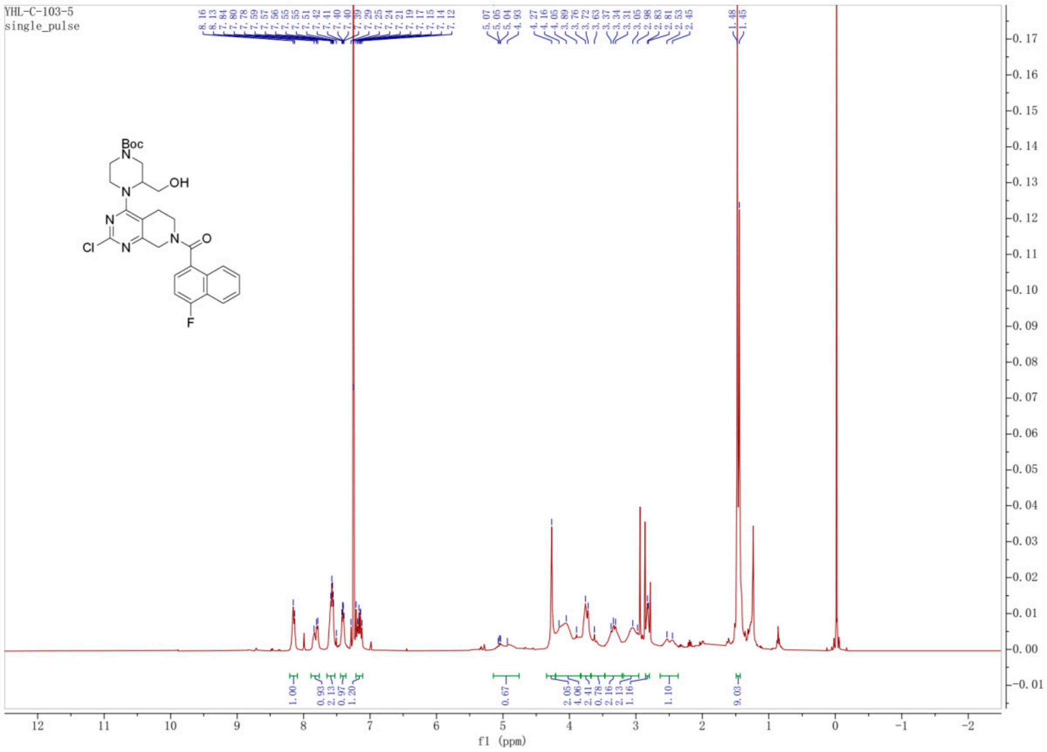

Figure S8. ^{1}H NMR spectrum of **8d**.

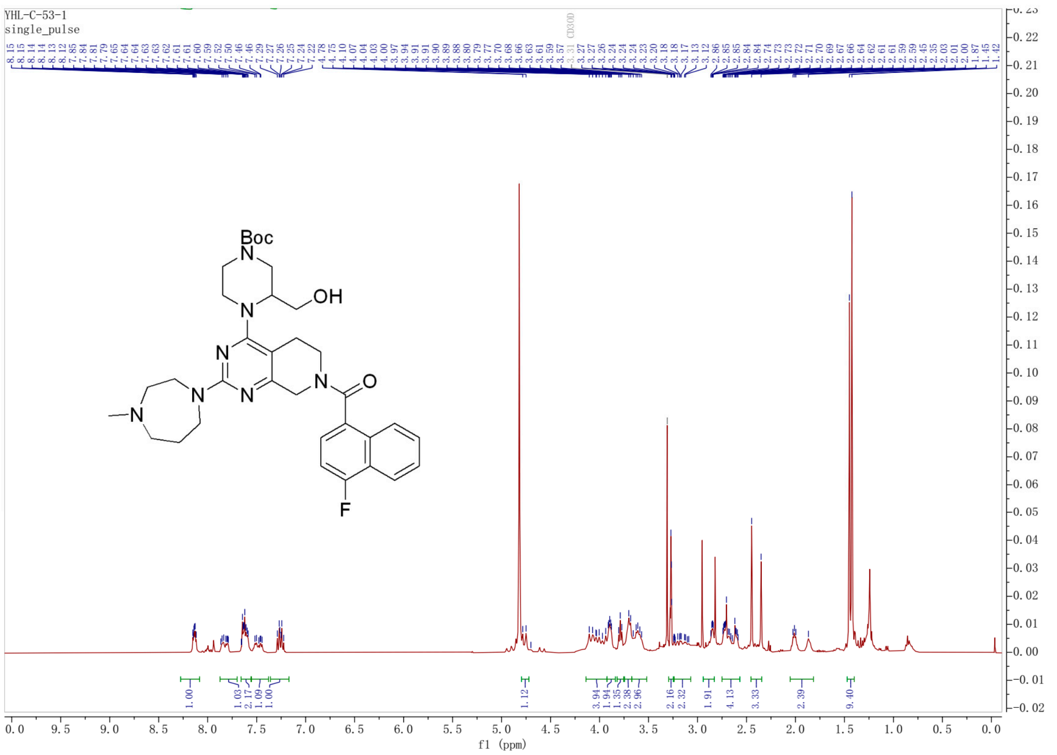

Figure S9. ^{1}H NMR spectrum of **9d**.

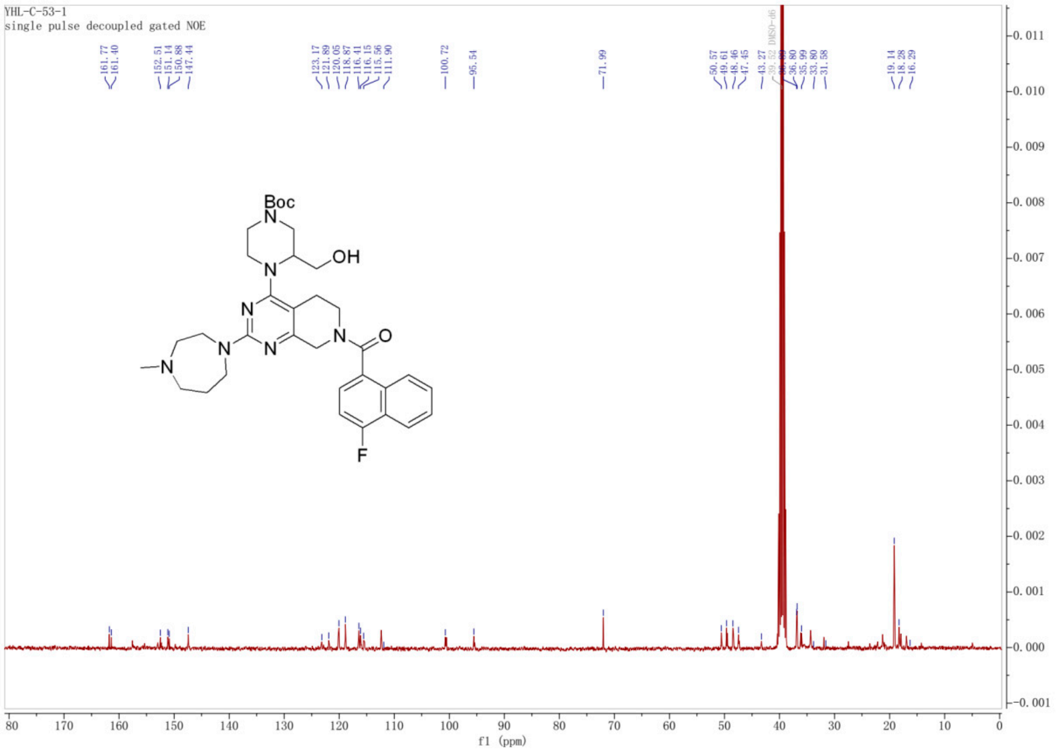

Figure S10. ^{13}C NMR spectrum of **9d**.

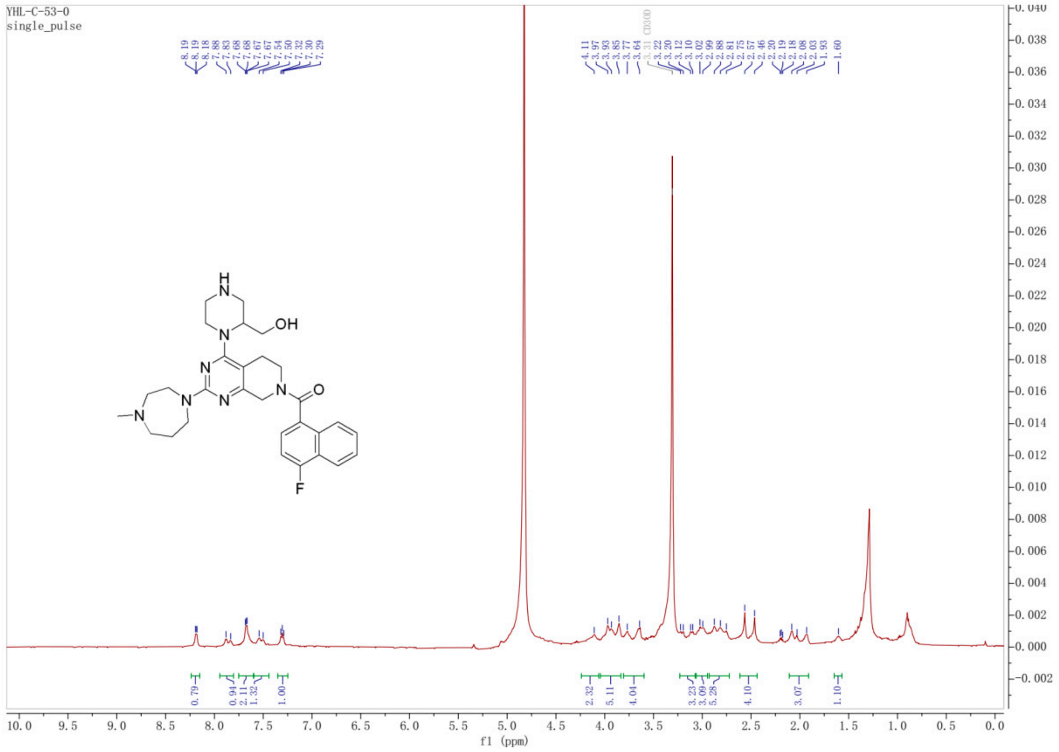

Figure S11. ^{1}H NMR spectrum of **10d**.

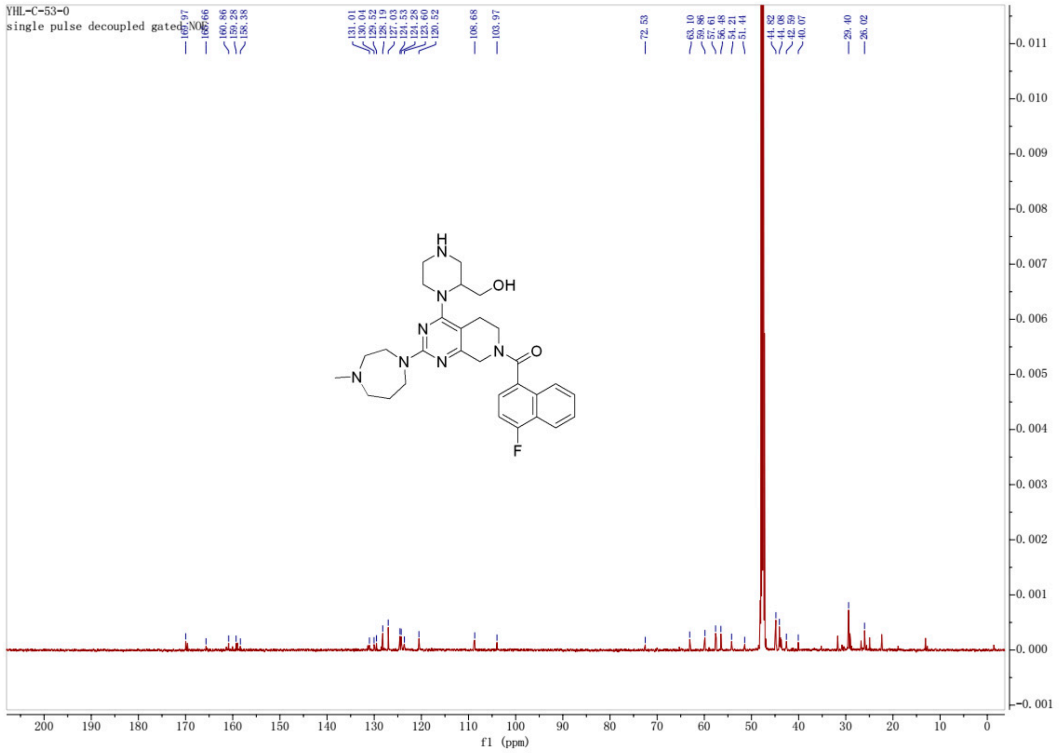

Figure S12. ^{13}C NMR spectrum of **10d**.

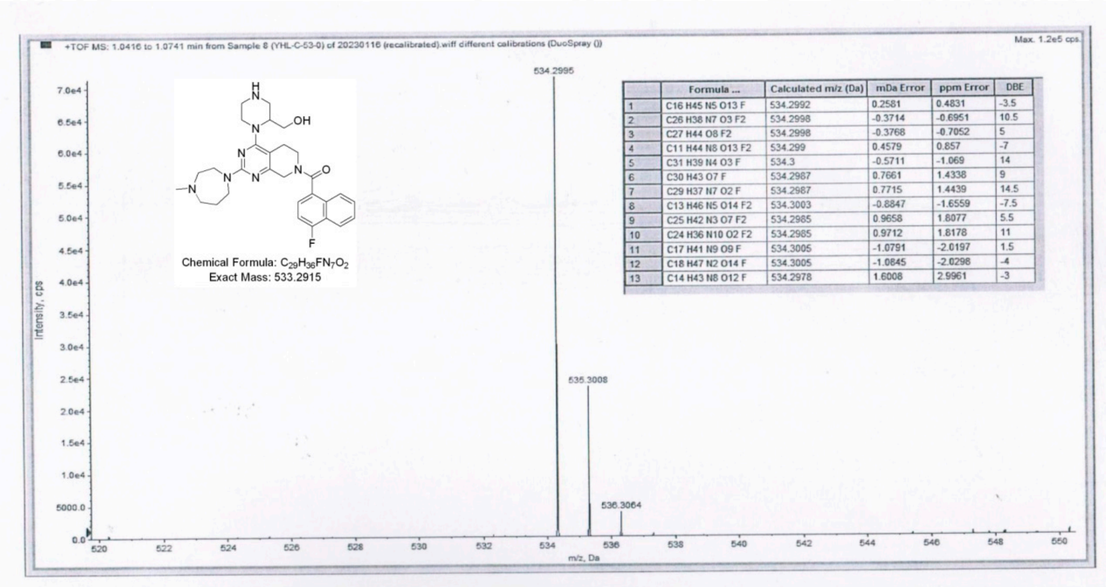

	Formula ...	Calculated m/z (Da)	mDa Error	ppm Error	DBE
1	C16 H45 N5 O13 F	534.2992	0.2581	0.4831	-3.5
2	C26 H38 N7 O3 F2	534.2998	-0.3714	-0.6951	10.5
3	C27 H44 O8 F2	534.2998	-0.3768	-0.7052	5
4	C11 H44 N8 O13 F2	534.299	0.4579	0.857	-7
5	C31 H39 N4 O3 F	534.3	-0.5711	-1.069	14
6	C30 H43 O7 F	534.2987	0.7661	1.4338	9
7	C29 H37 N7 O2 F	534.2987	0.7715	1.4439	14.5
8	C13 H46 N5 O14 F2	534.3003	-0.8847	-1.6559	-7.5
9	C25 H42 N3 O7 F2	534.2985	0.9658	1.8077	5.5
10	C24 H36 N10 O2 F2	534.2985	0.9712	1.8178	11
11	C17 H41 N9 O9 F	534.3005	-1.0791	-2.0197	1.5
12	C18 H47 N2 O14 F	534.3005	-1.0845	-2.0298	-4
13	C14 H43 N8 O12 F	534.2978	1.6008	2.9961	-3

Figure S13. HR-MS chromatogram spectrum of **10d**.

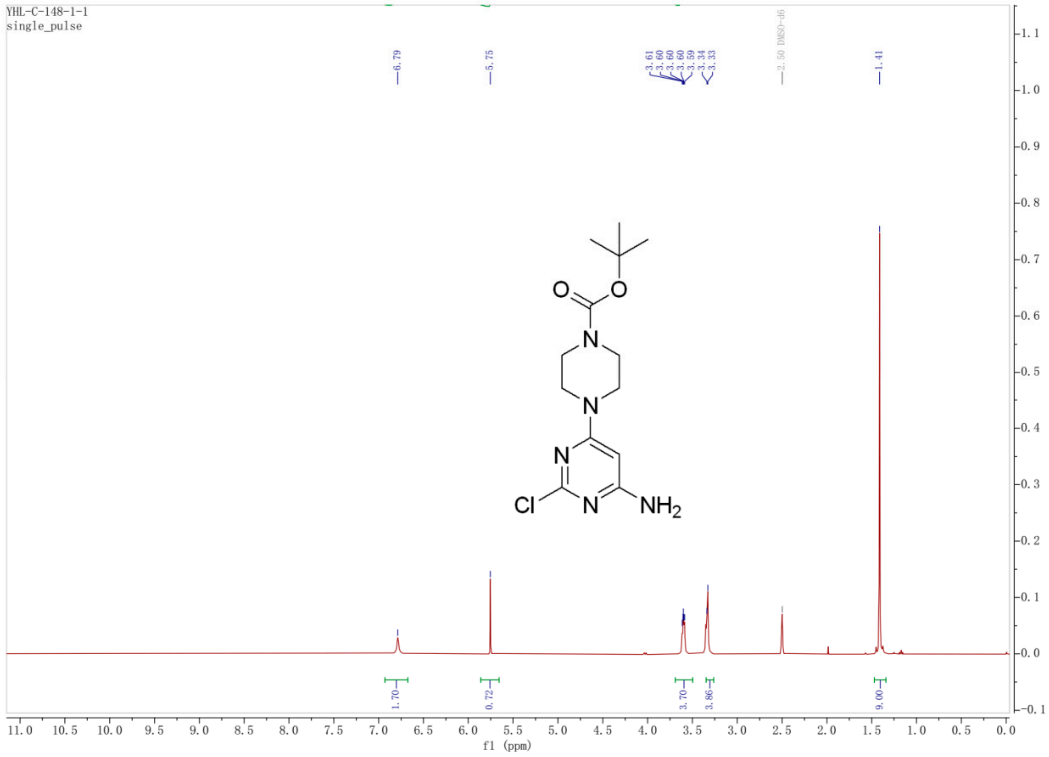

Figure S14. ^{1}H NMR spectrum of **19**.

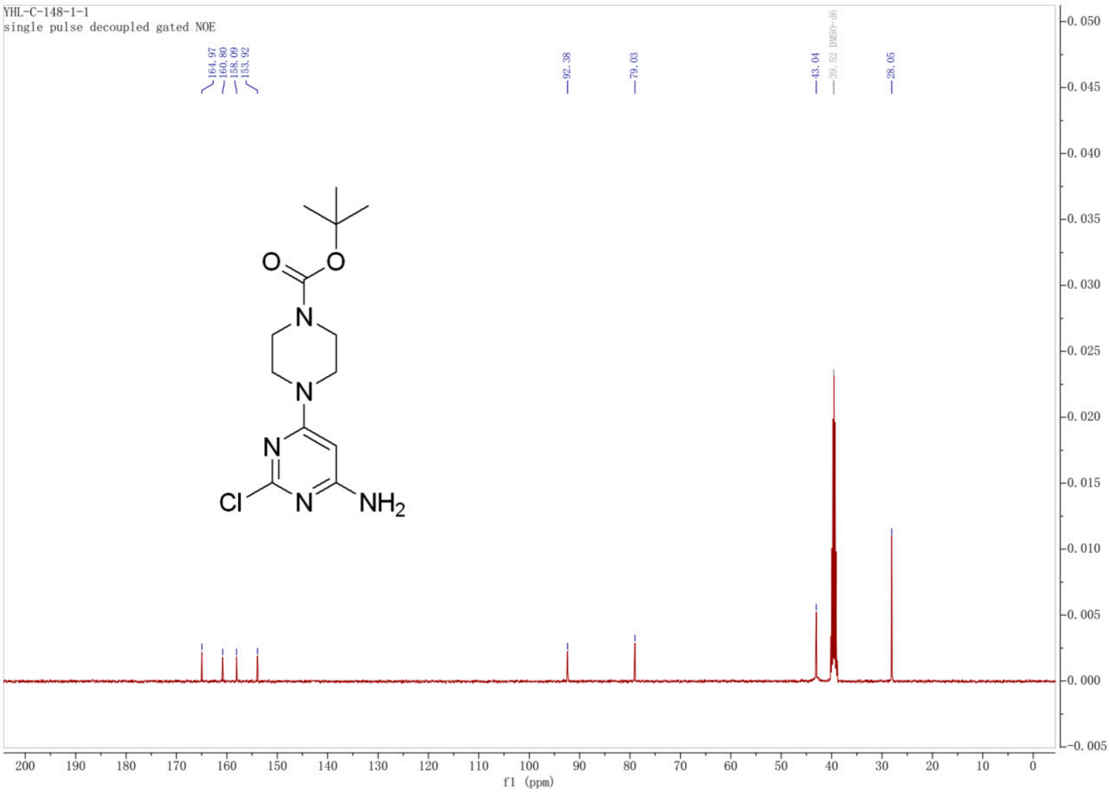

Figure S15. ^{13}C NMR spectrum of **19**.

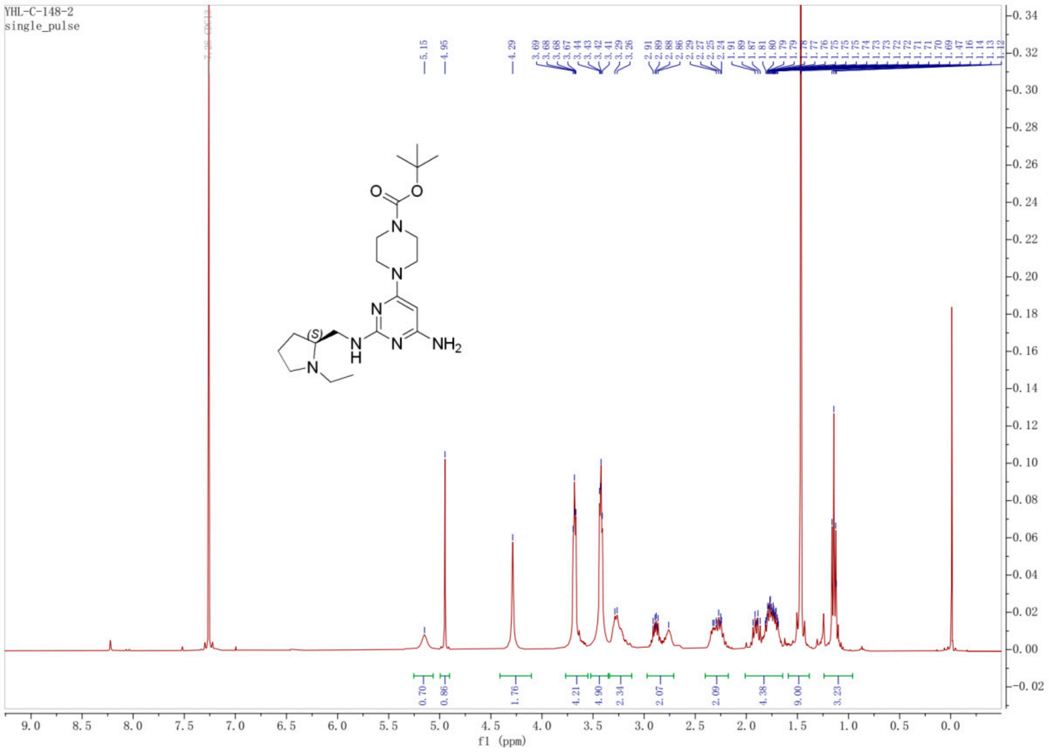

Figure S16. ^{1}H NMR spectrum of **20**.

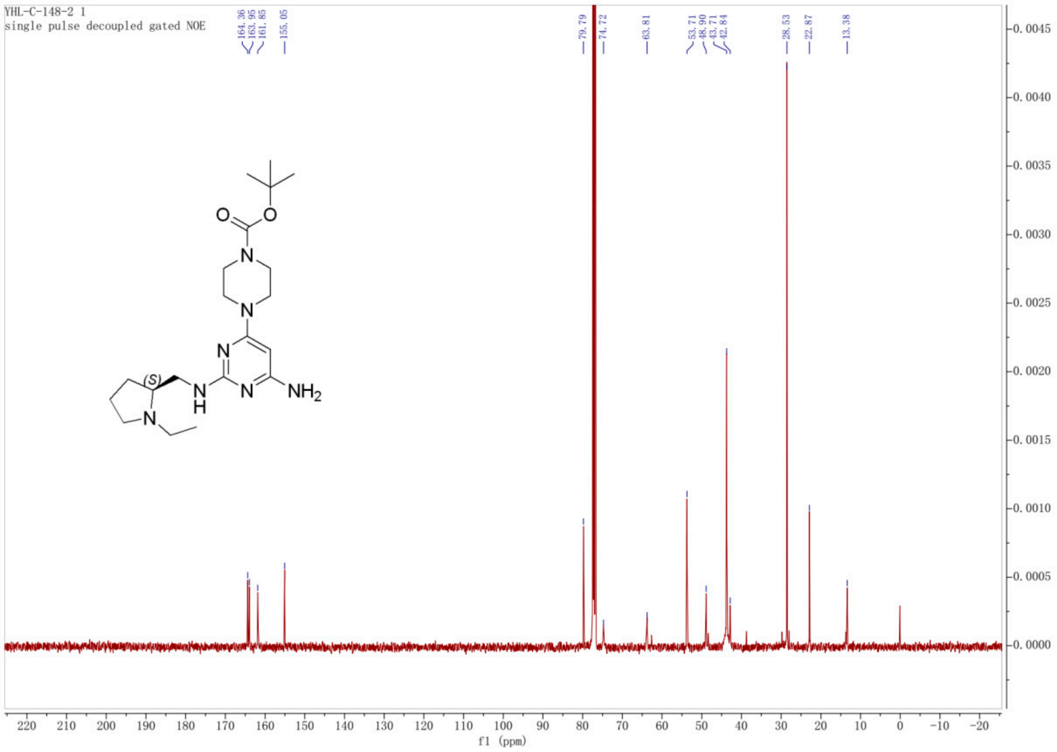

Figure S17. ^{13}C NMR spectrum of **20**.

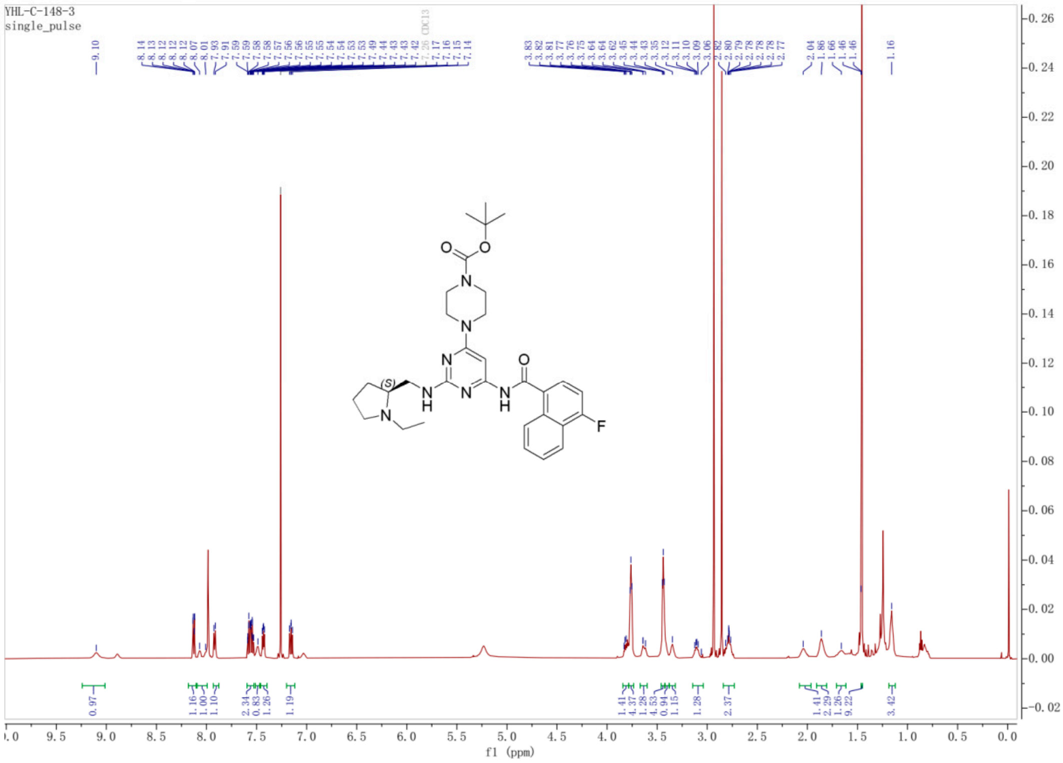

Figure S18. ^{1}H NMR spectrum of **21**.

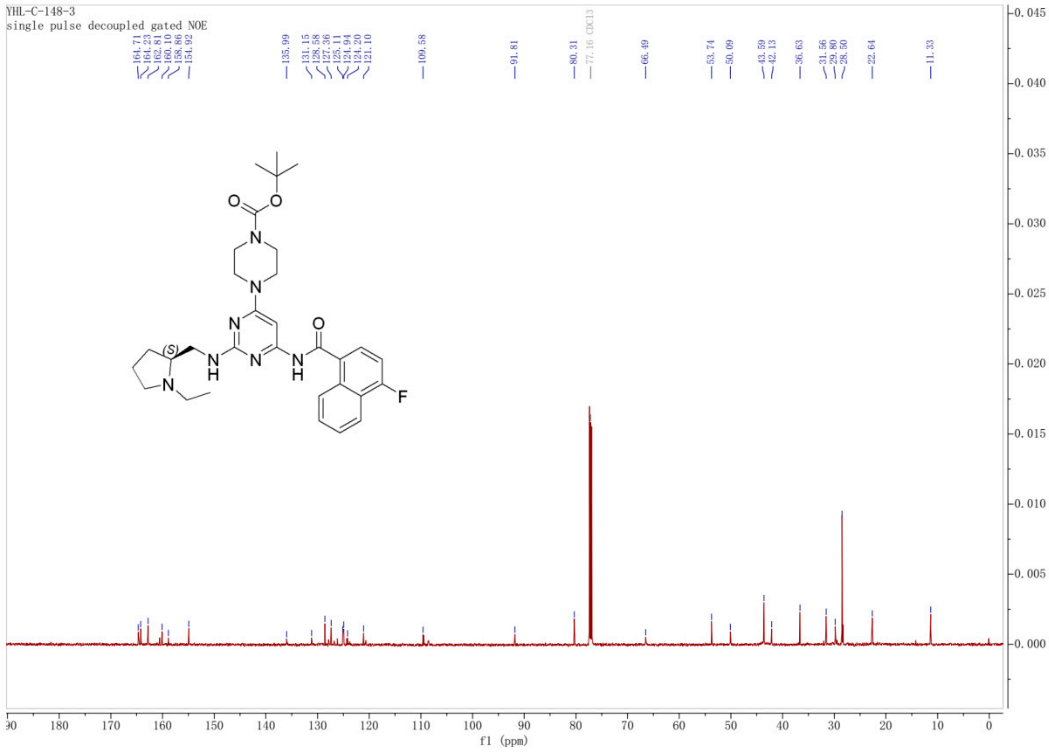

Figure S19. ^{13}C NMR spectrum of **21**.

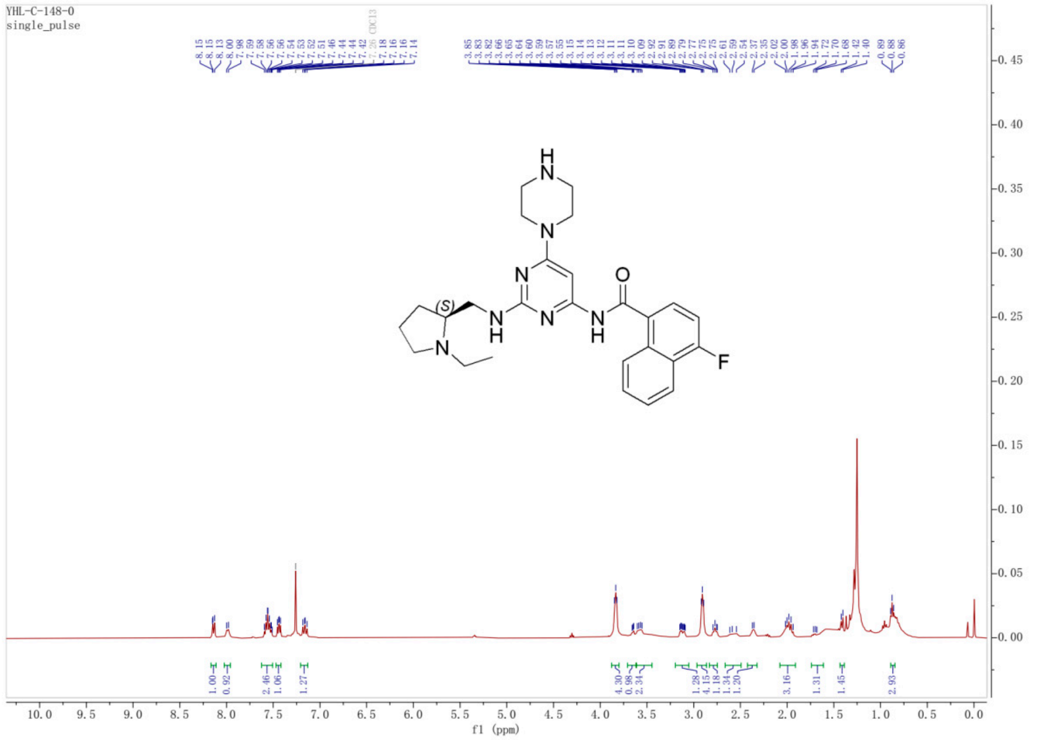

Figure S20. ^{1}H NMR spectrum of **10j**.

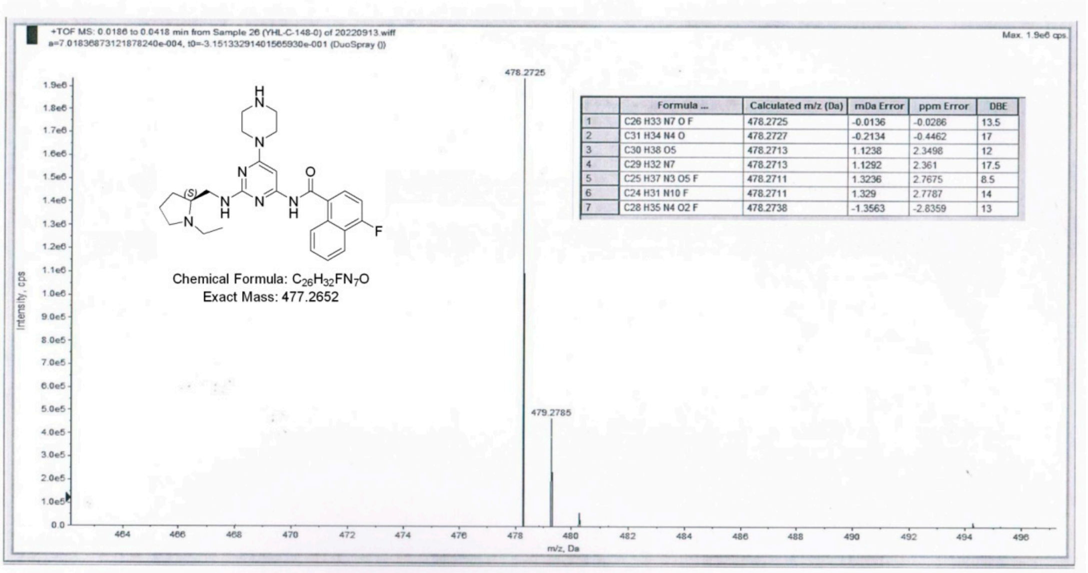

	Formula ...	Calculated m/z (Da)	mDa Error	ppm Error	DBE
1	C26 H33 N7 O F	478.2725	-0.0136	-0.0286	13.5
2	C31 H34 N4 O	478.2727	-0.2134	-0.4462	17
3	C30 H38 O5	478.2713	1.1238	2.3498	12
4	C29 H32 N7	478.2713	1.1292	2.361	17.5
5	C25 H37 N3 O5 F	478.2711	1.3236	2.7675	8.5
6	C24 H31 N10 F	478.2711	1.329	2.7787	14
7	C28 H35 N4 O2 F	478.2738	-1.3563	-2.8359	13

Figure S21. HR-MS chromatogram spectrum of **10j**.

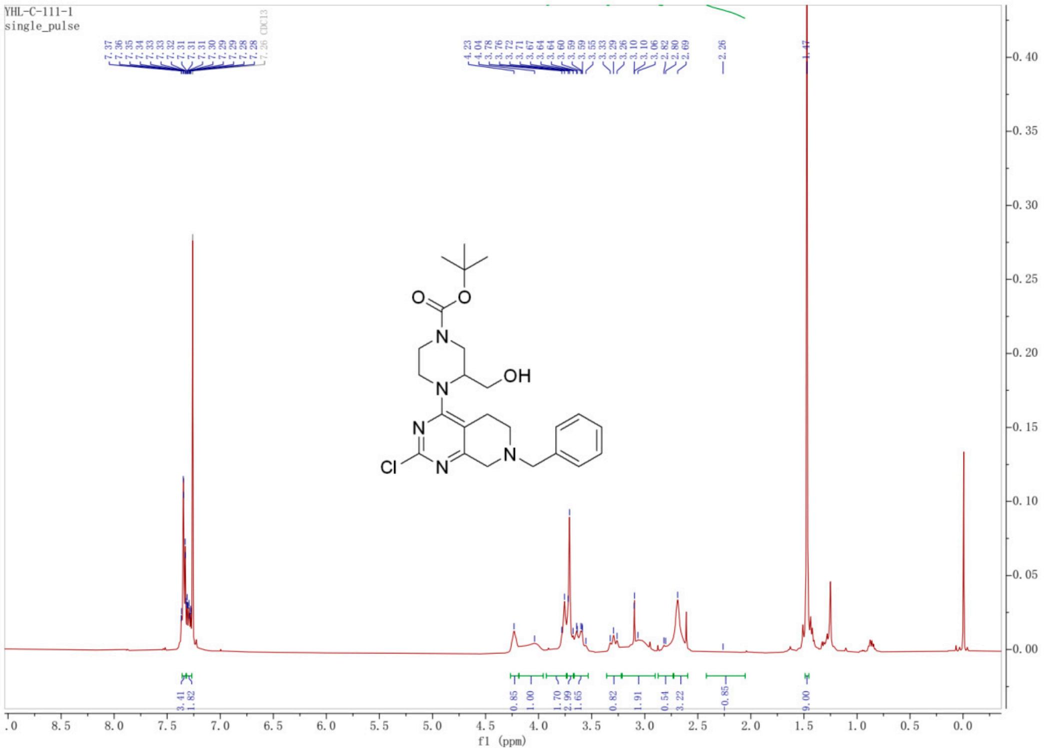

Figure S22. ^{1}H NMR spectrum of **12**.

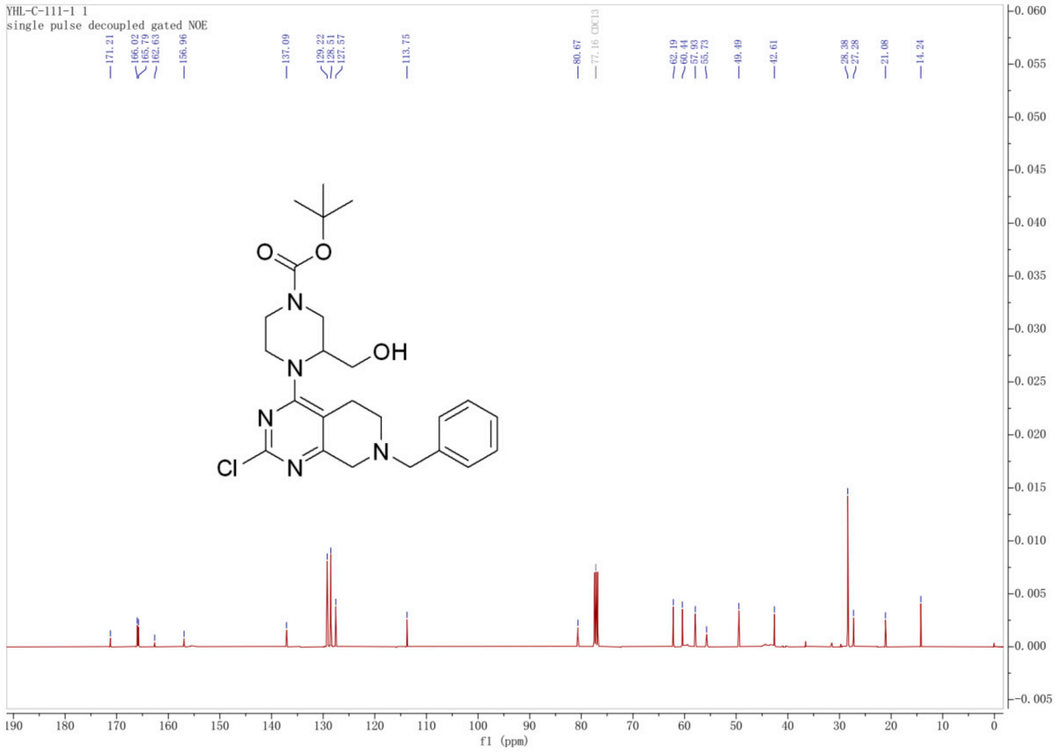

Figure S23. ^{13}C NMR spectrum of **12**.

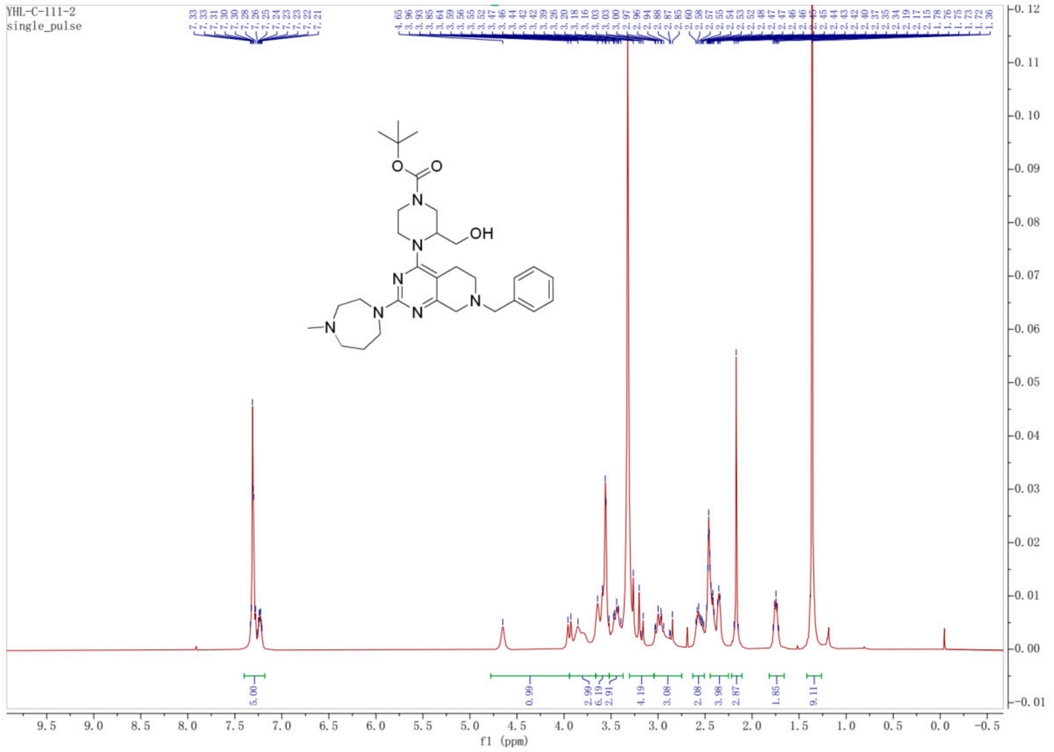

Figure S24. ^{1}H NMR spectrum of **13**.

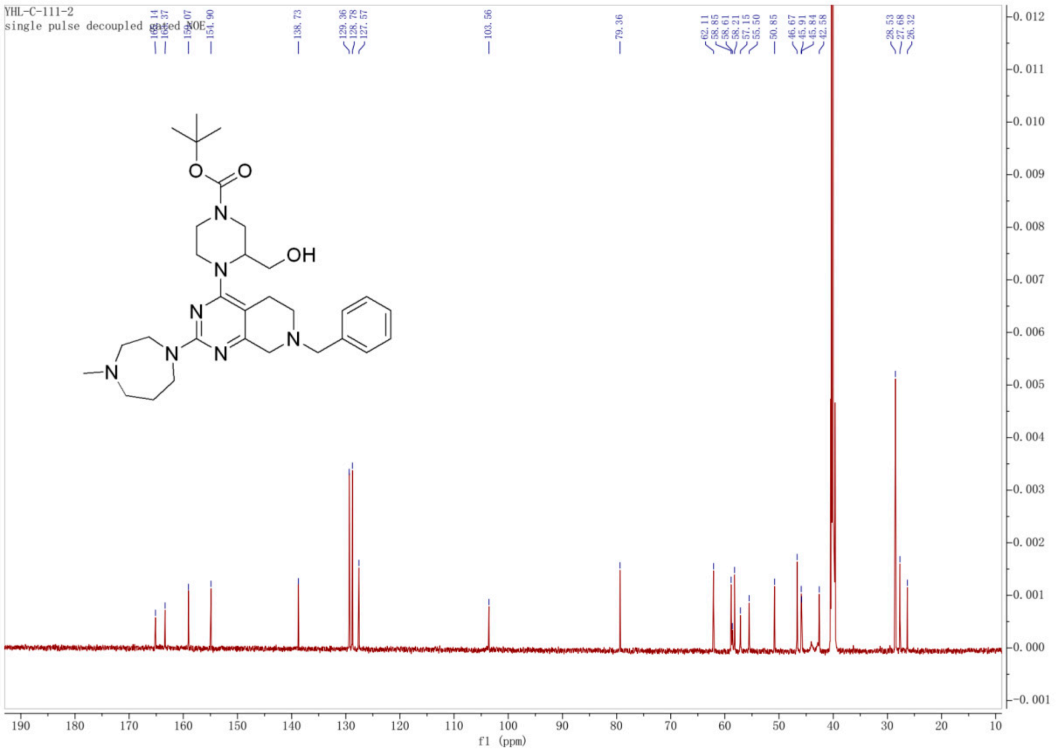

Figure S25. ^{13}C NMR spectrum of **13**.

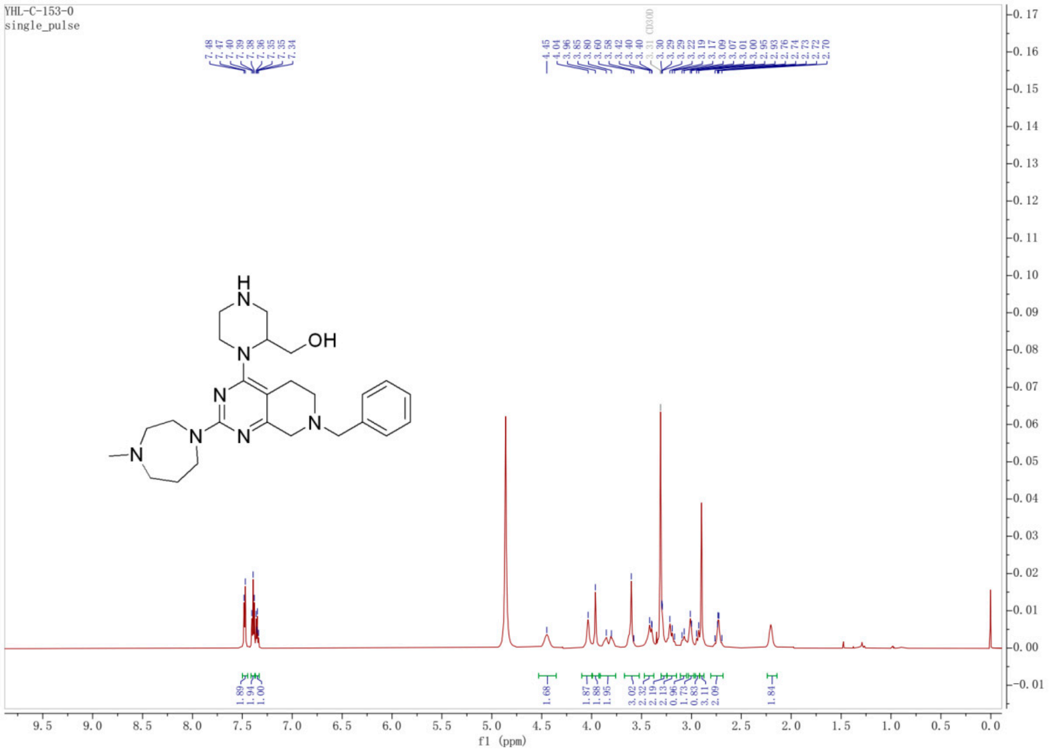

Figure S26. ^{1}H NMR spectrum of **10h**.

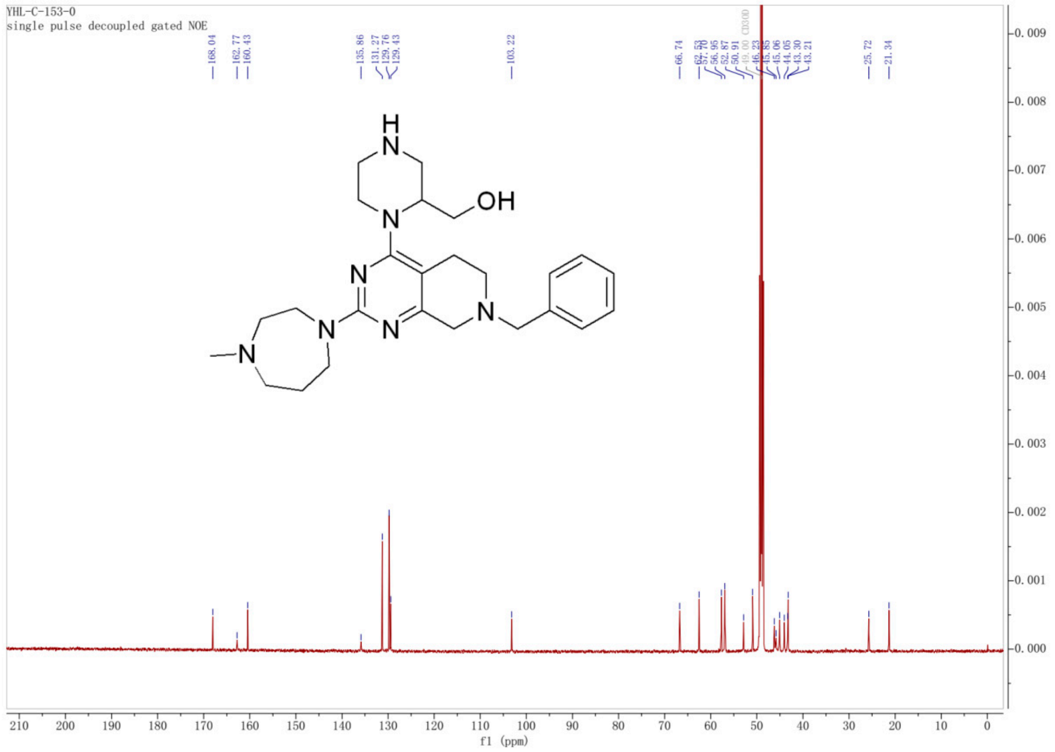

Figure S27. ^{13}C NMR spectrum of **10h**.

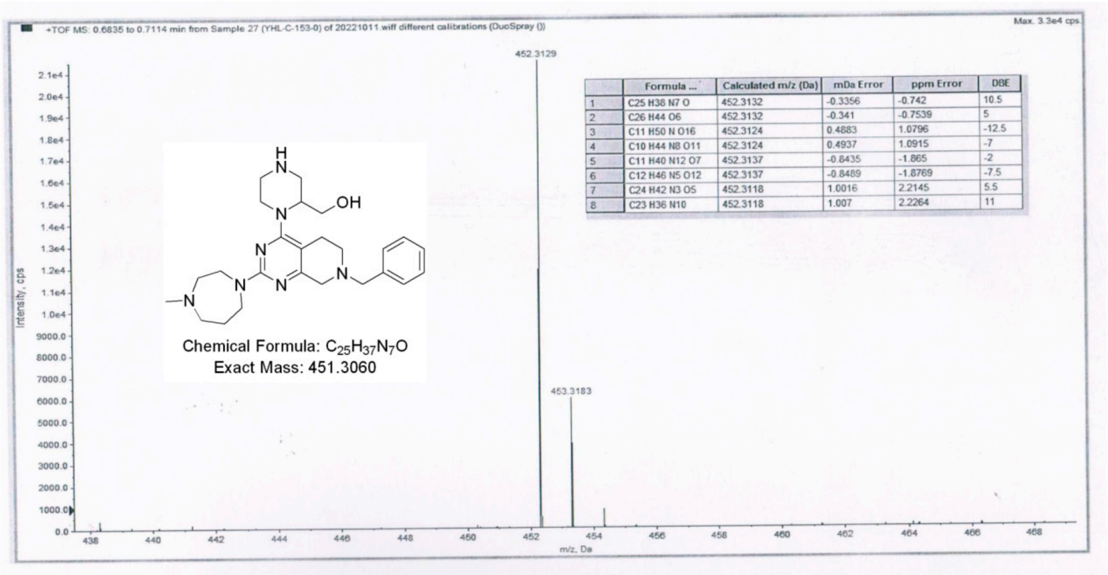

Figure S28. HR-MS chromatogram spectrum of **10h**.

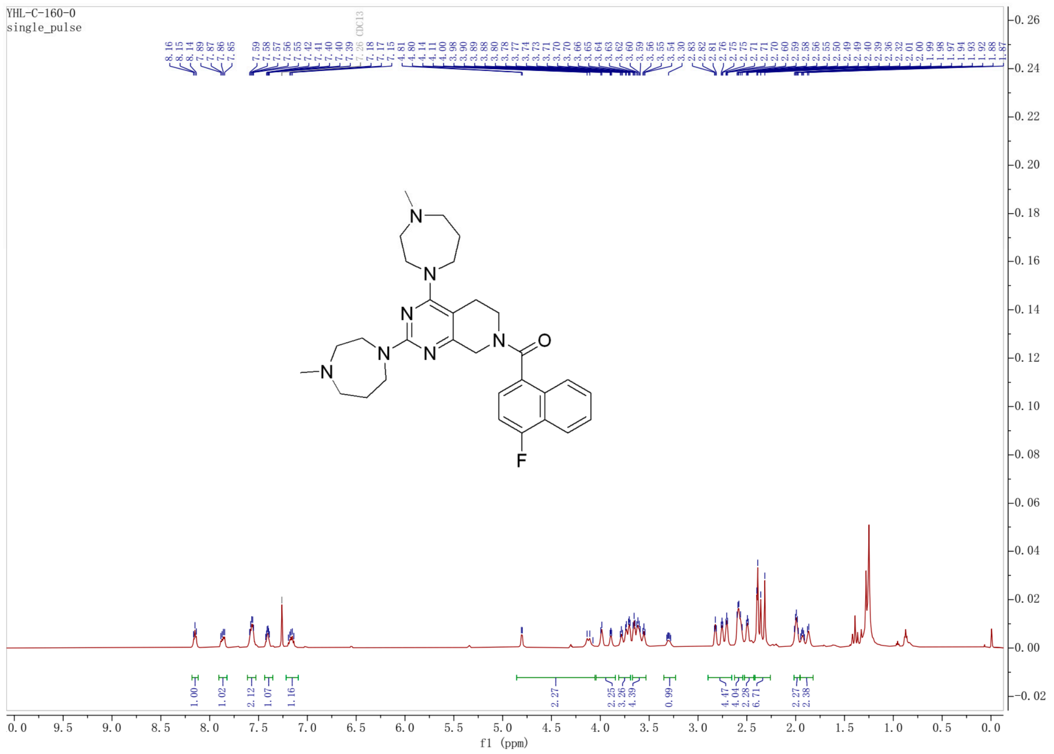

Figure S29. ^{1}H NMR spectrum of **10f**.

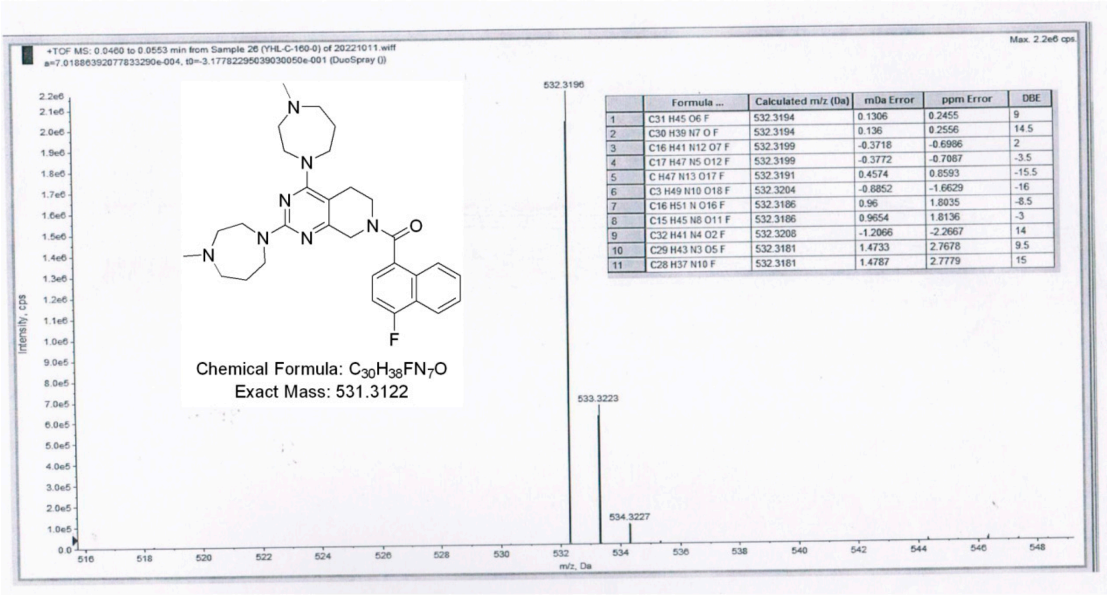

Figure S30. HR-MS chromatogram spectrum of **10f**.

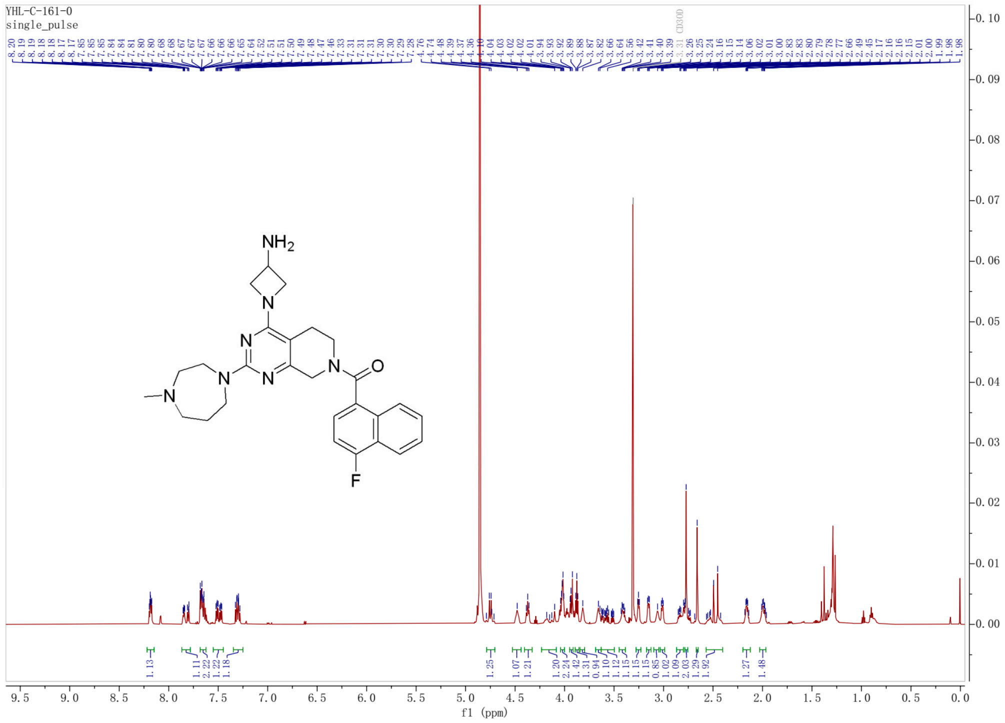

Figure S31. ^{1}H NMR spectrum of **10a**.

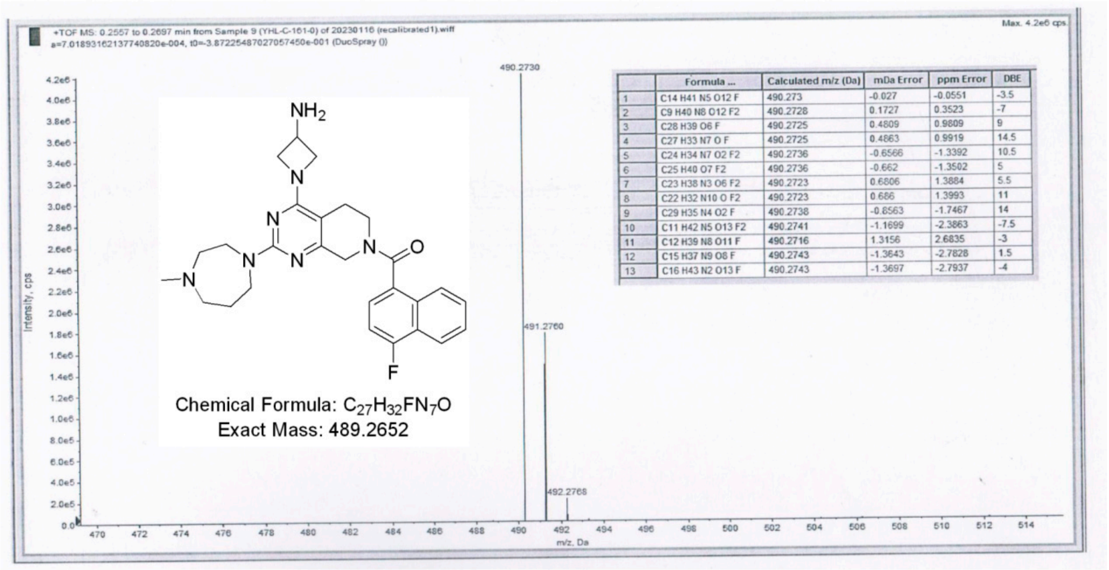

Figure S32. HR-MS chromatogram spectrum of **10a**.

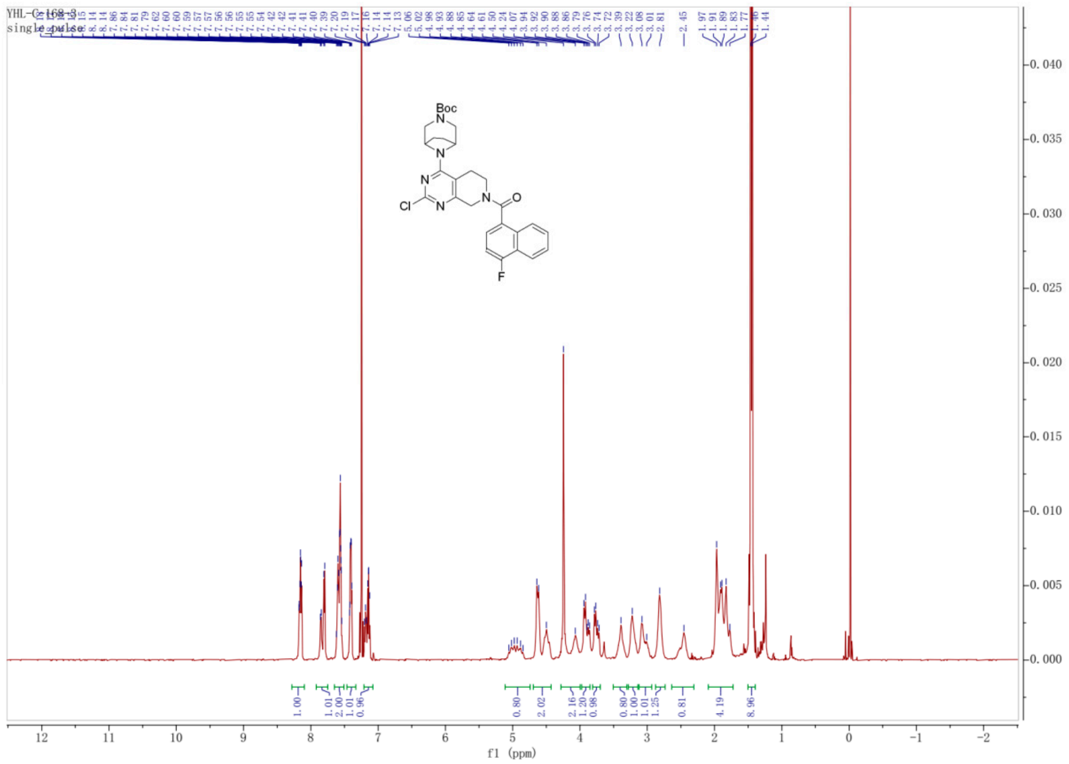

Figure S33. ^{1}H NMR spectrum of **8c**.

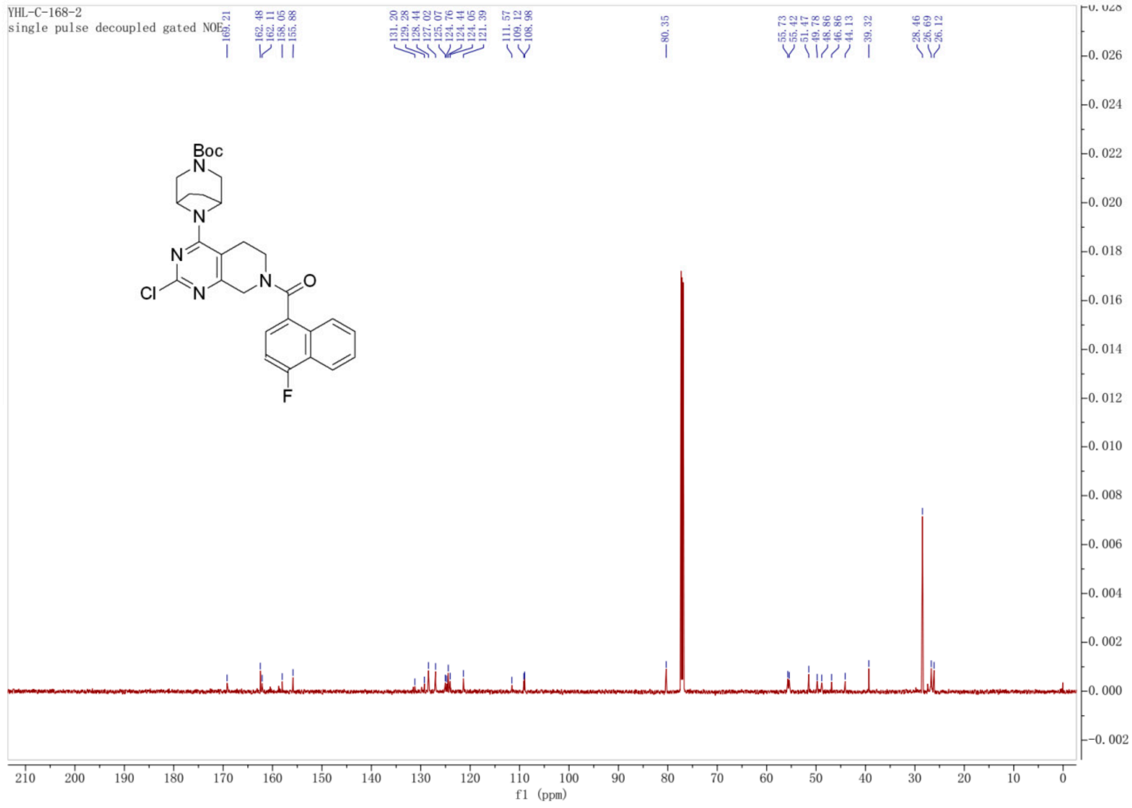

Figure S34. ^{13}C NMR spectrum of **8c**.

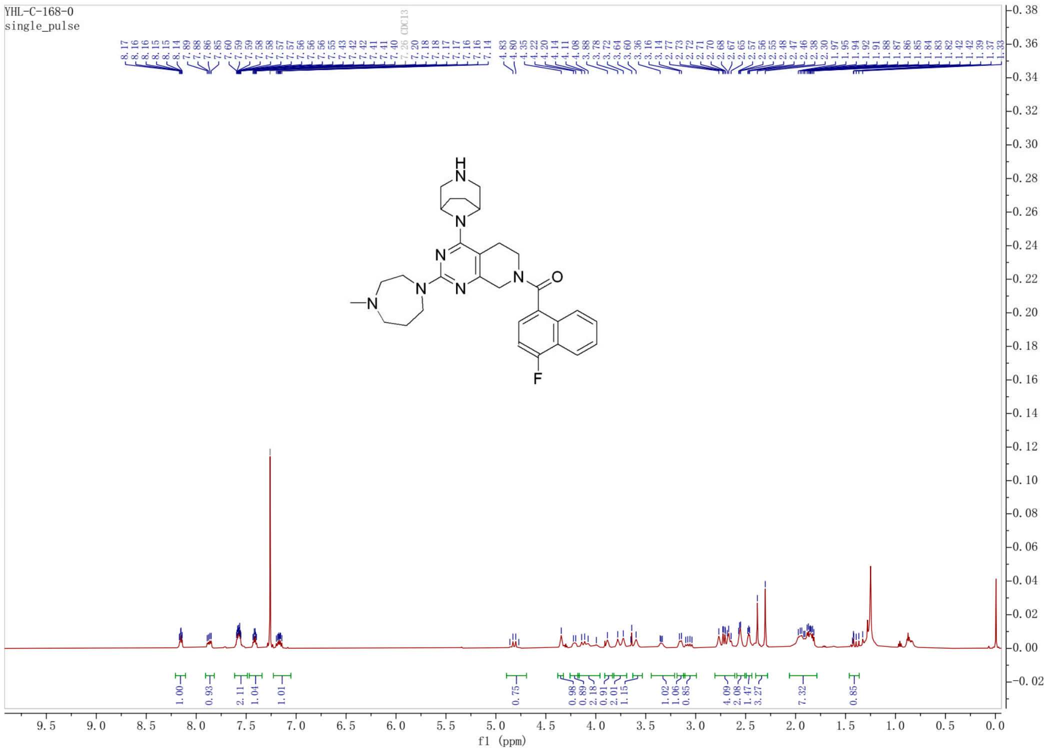

Figure S35. ^{1}H NMR spectrum of **10c**.

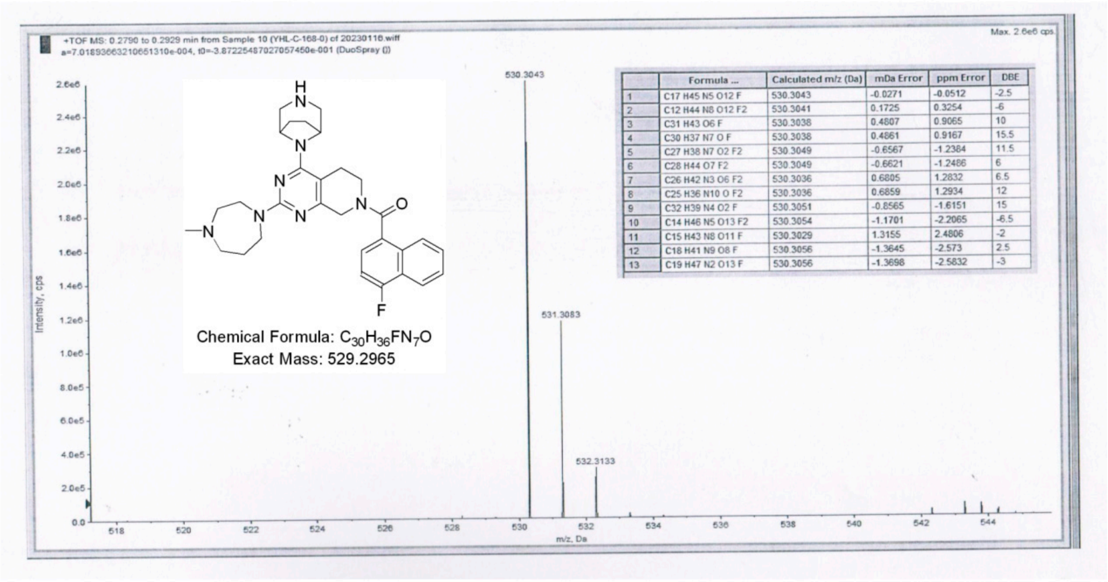

	Formula ...	Calculated m/z (Da)	mDa Error	ppm Error	DBE
1	C17 H45 N5 O12 F	530.3043	-0.0271	-0.0512	-2.5
2	C12 H44 N8 O12 F2	530.3041	0.1725	0.3254	-6
3	C31 H43 O6 F	530.3038	0.4807	0.9065	10
4	C30 H37 N7 O F	530.3038	0.4861	0.9167	15.5
5	C27 H38 N7 O2 F2	530.3049	-0.6567	-1.2384	11.5
6	C28 H44 O7 F2	530.3049	-0.6621	-1.2486	6
7	C26 H42 N3 O6 F2	530.3036	0.6805	1.2832	6.5
8	C25 H36 N10 O F2	530.3036	0.6859	1.2934	12
9	C32 H39 N4 O2 F	530.3051	-0.8565	-1.6151	15
10	C14 H46 N5 O13 F2	530.3054	-1.1701	-2.2065	-6.5
11	C15 H43 N8 O11 F	530.3029	1.3155	2.4806	-2
12	C18 H41 N9 O8 F	530.3056	-1.3645	-2.573	2.5
13	C19 H47 N2 O13 F	530.3056	-1.3698	-2.5832	-3

Figure S36. HR-MS chromatogram spectrum of **10c**.

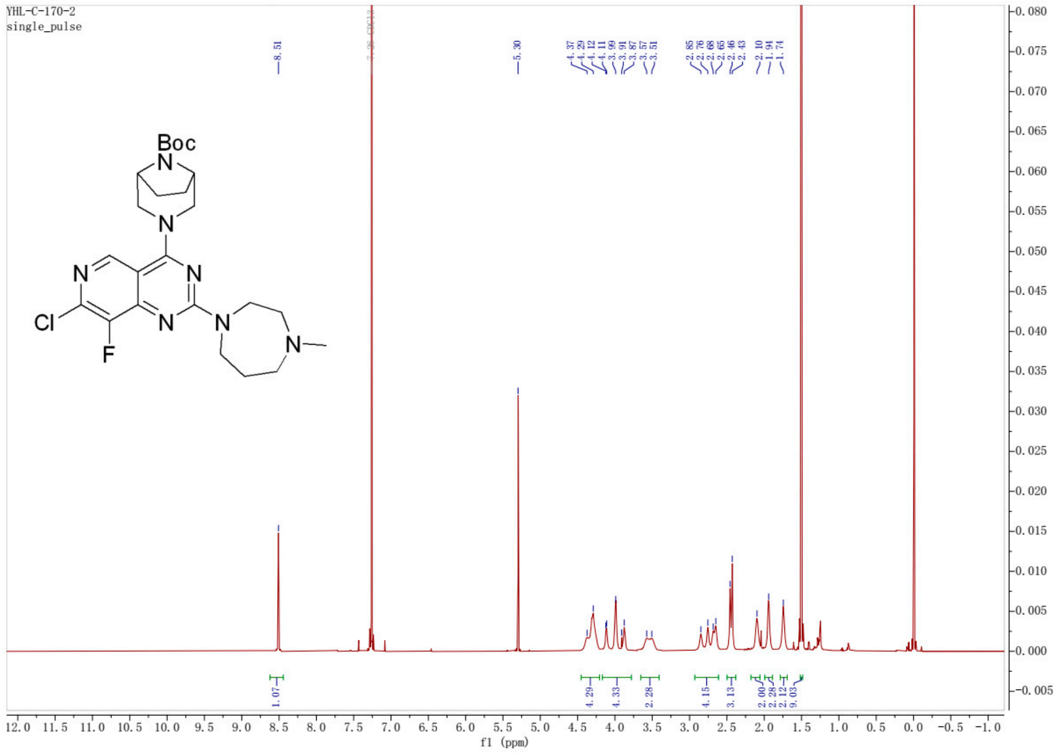

Figure S37. ^{1}H NMR spectrum of **23**.

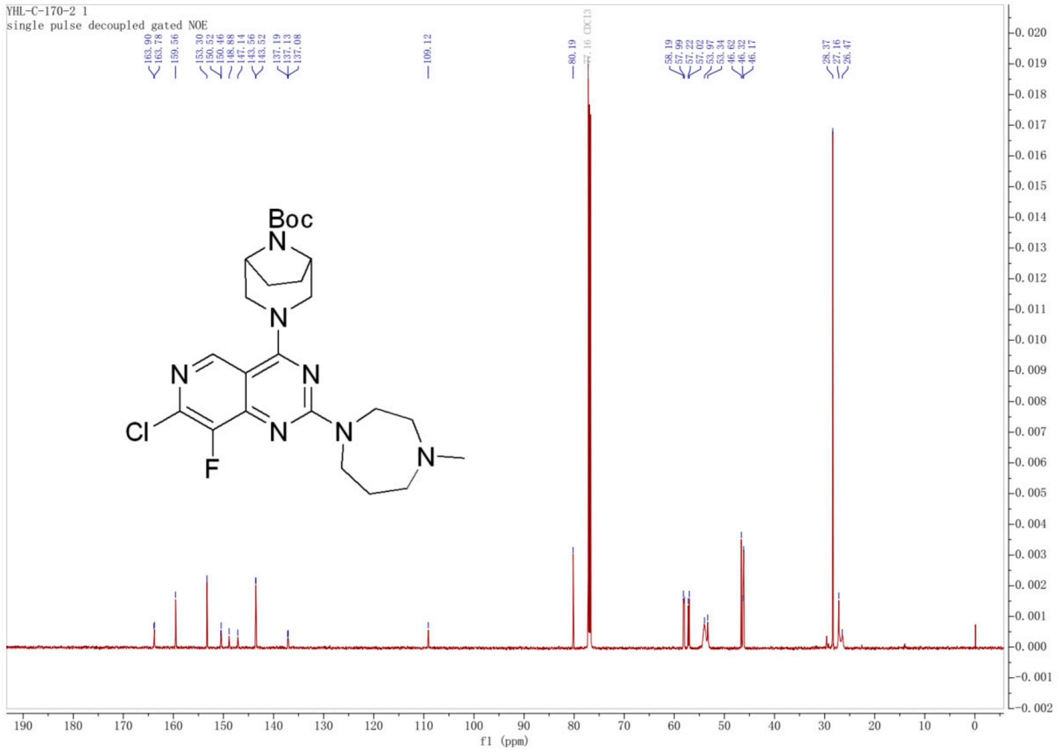

Figure S38. ^{13}C NMR spectrum of **23**.

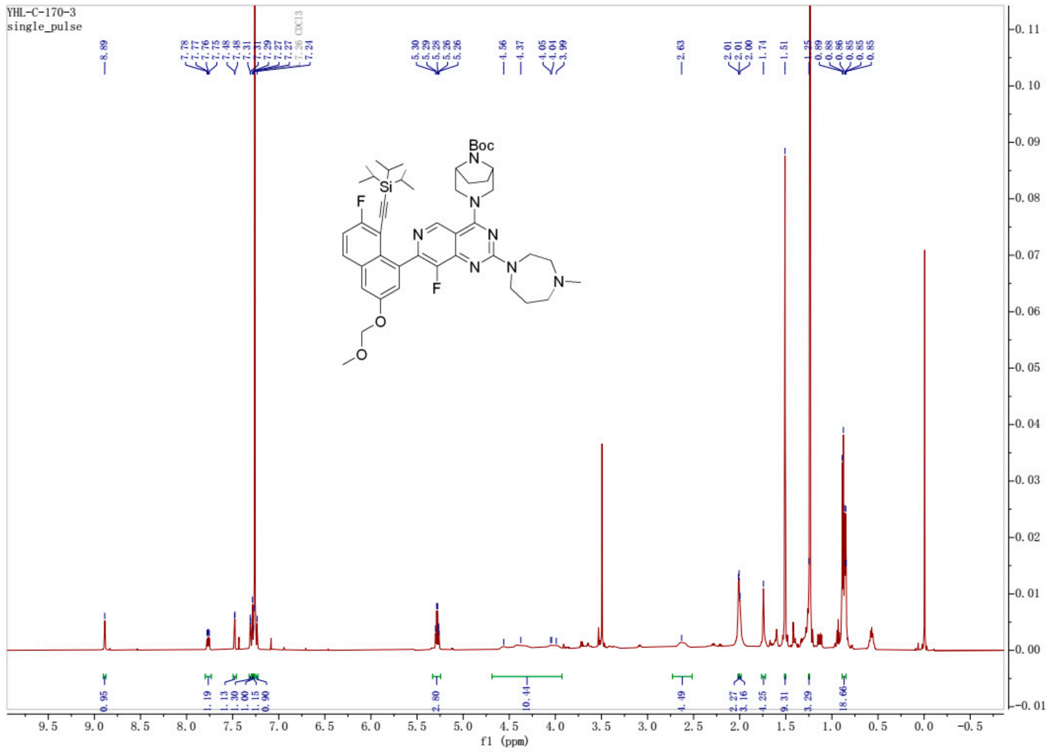

Figure S39. ^{1}H NMR spectrum of **24**.

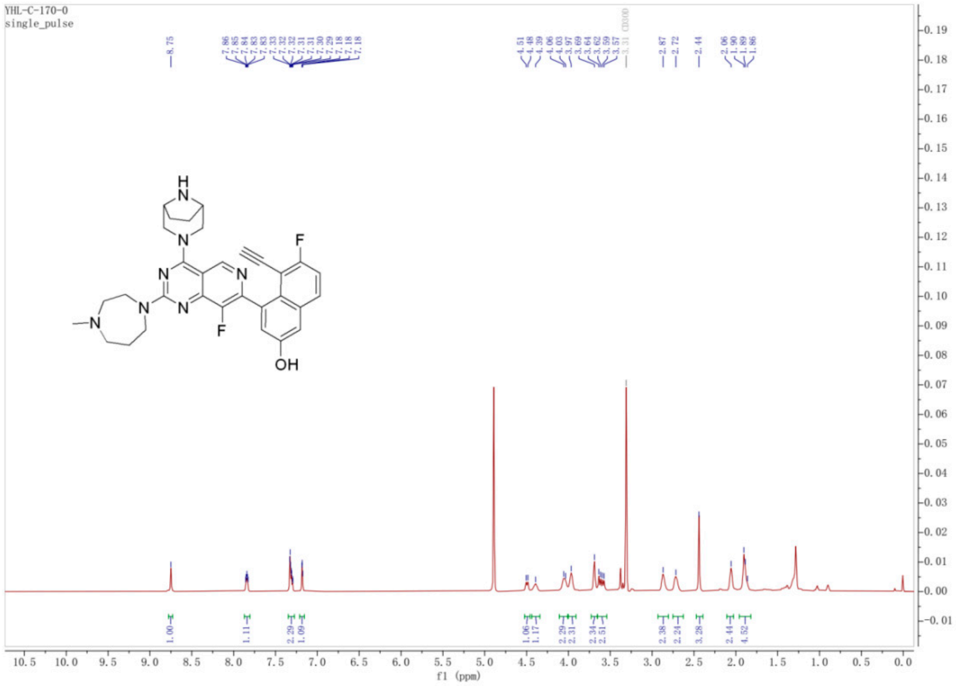

Figure S40. ^{1}H NMR spectrum of **10k**.

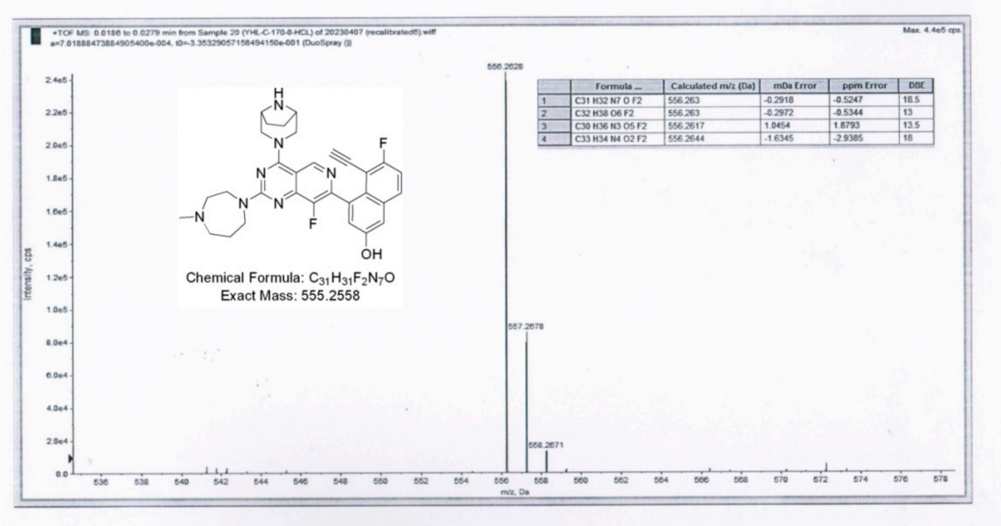

Figure S41. HR-MS chromatogram spectrum of **10k**.

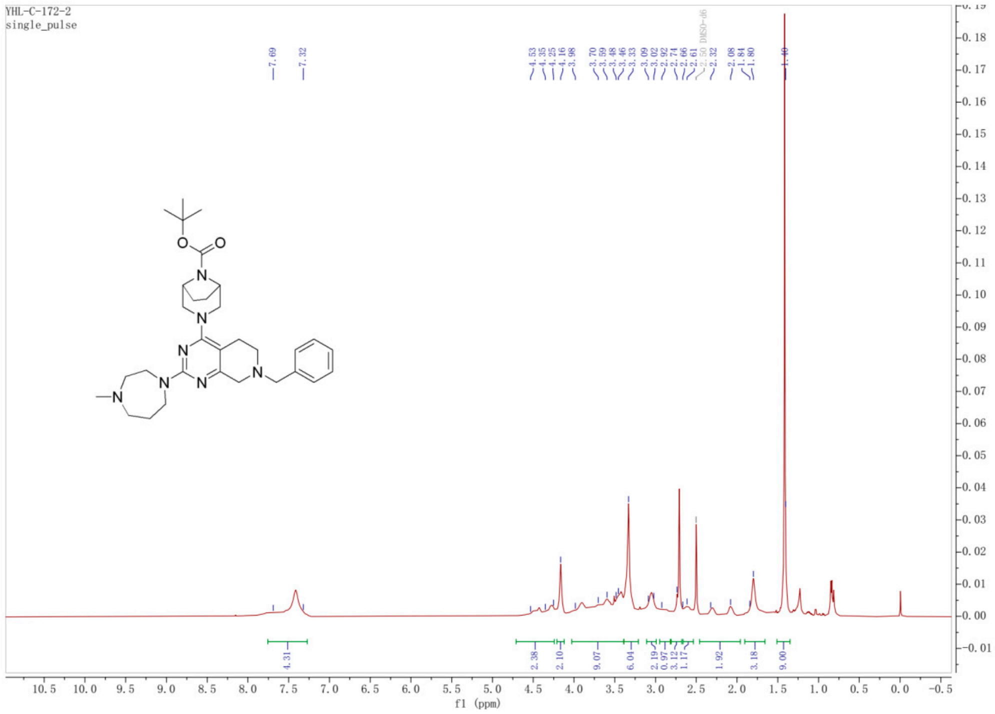

Figure S42. ^{1}H NMR spectrum of **15**.

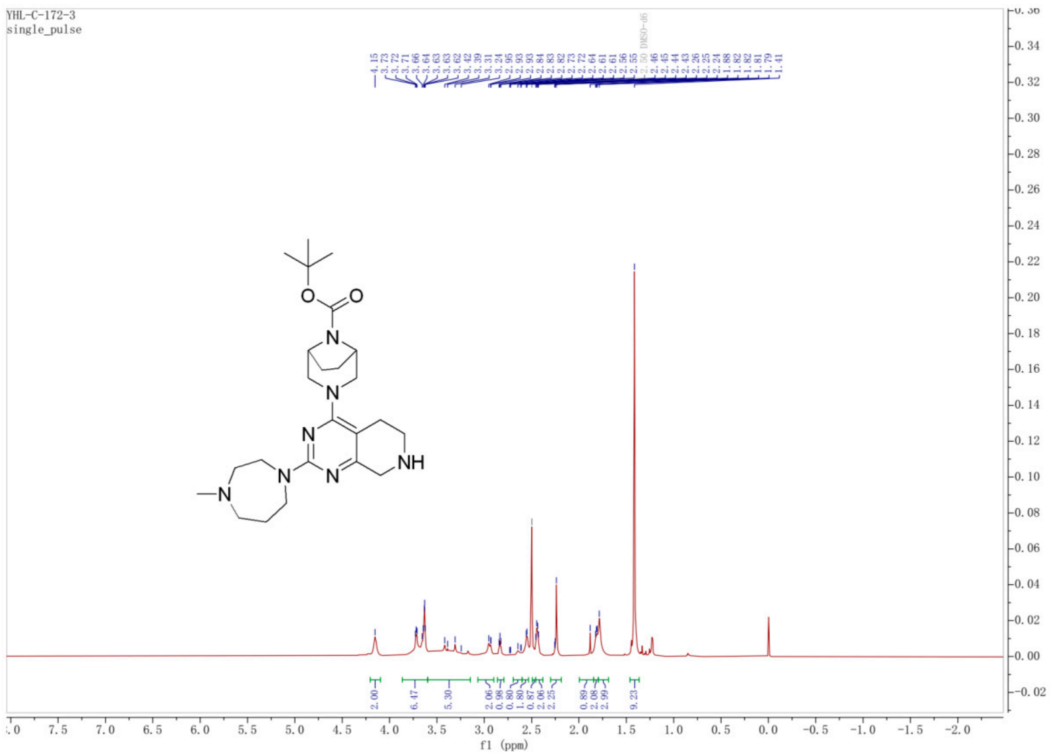

Figure S43. ^{1}H NMR spectrum of **16**.

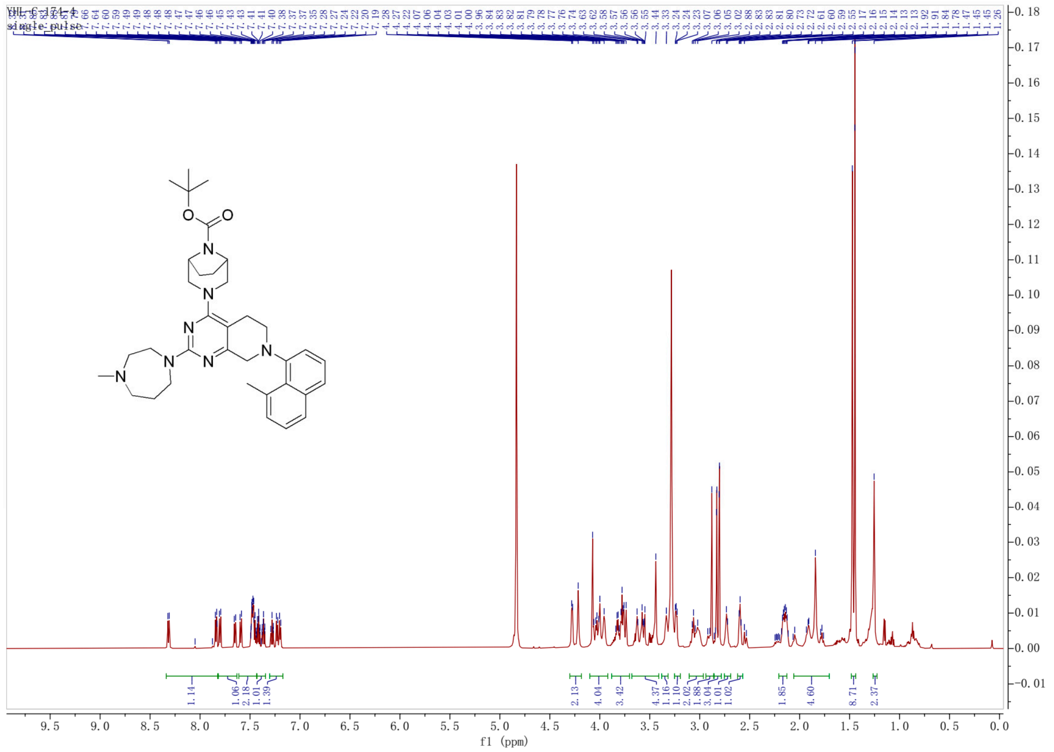

Figure S44. ^{1}H NMR spectrum of **17**.

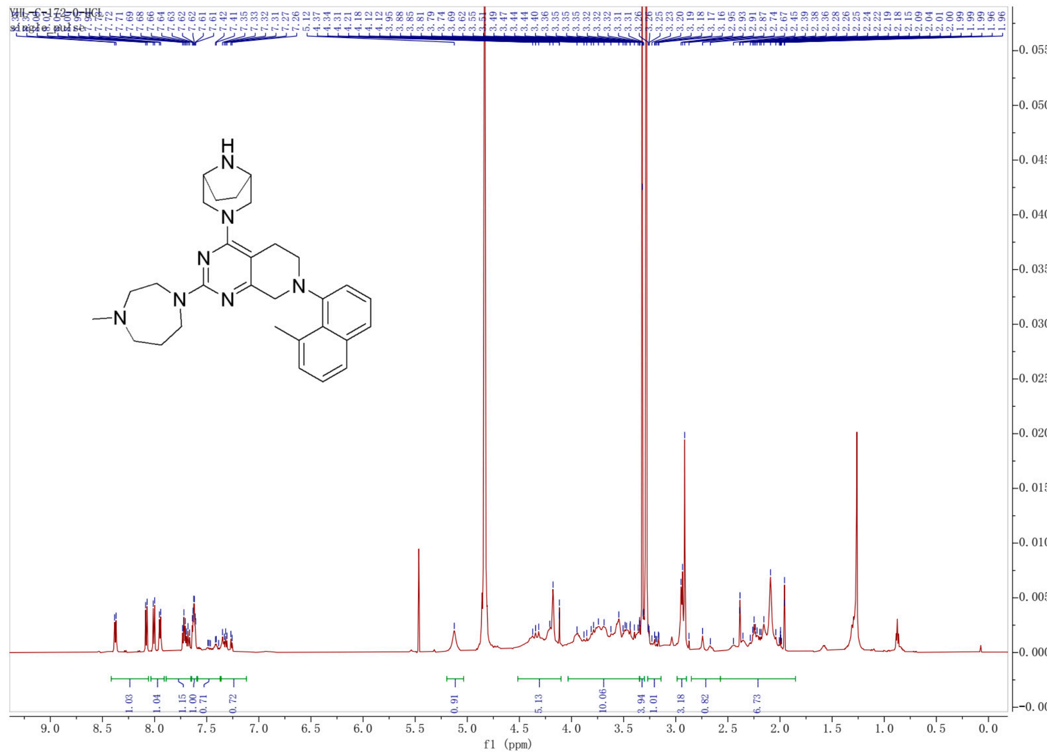

Figure S45. ^{1}H NMR spectrum of **10i**.

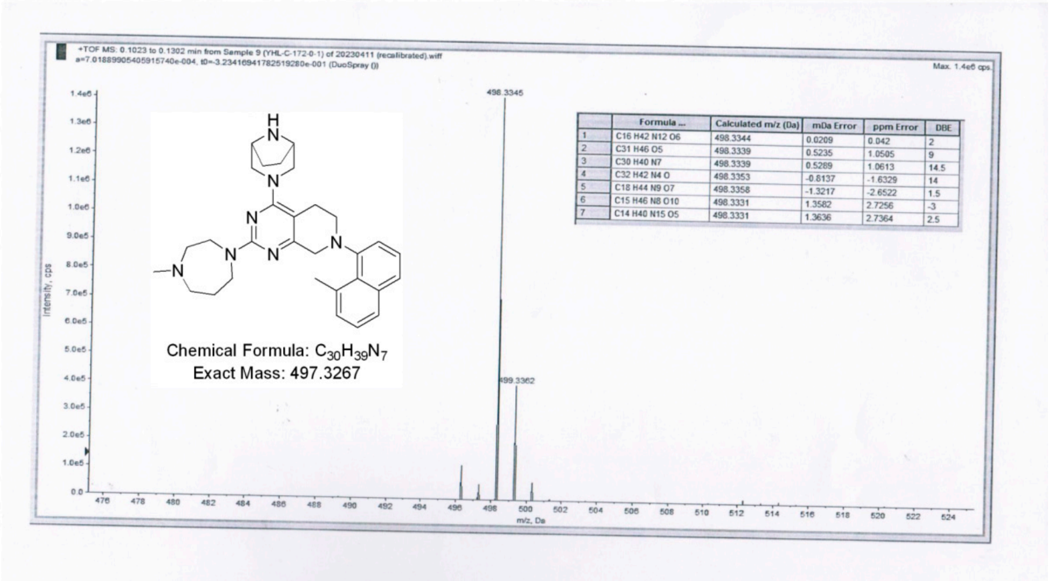

	Formula ...	Calculated m/z (Da)	mDa Error	ppm Error	DBE
1	C16 H42 N12 O6	498.3344	0.0209	0.042	2
2	C31 H46 O5	498.3339	0.5235	1.0505	9
3	C30 H40 N7	498.3339	0.5289	1.0613	14.5
4	C32 H42 N4 O	498.3353	-0.8137	-1.6329	14
5	C18 H44 N9 O7	498.3358	-1.3217	-2.6522	1.5
6	C15 H46 N8 O10	498.3331	1.3582	2.7256	-3
7	C14 H40 N15 O5	498.3331	1.3636	2.7364	2.5

Figure S46. HR-MS chromatogram spectrum of **10i**.

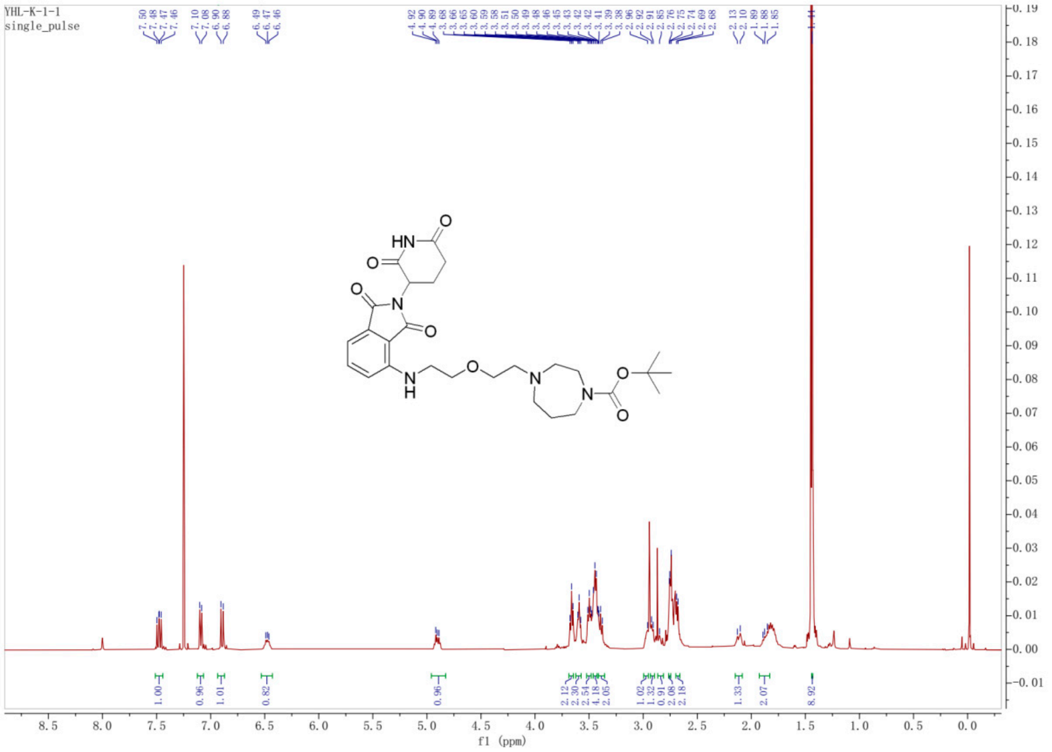

Figure S47. ^{1}H NMR spectrum of **28**.

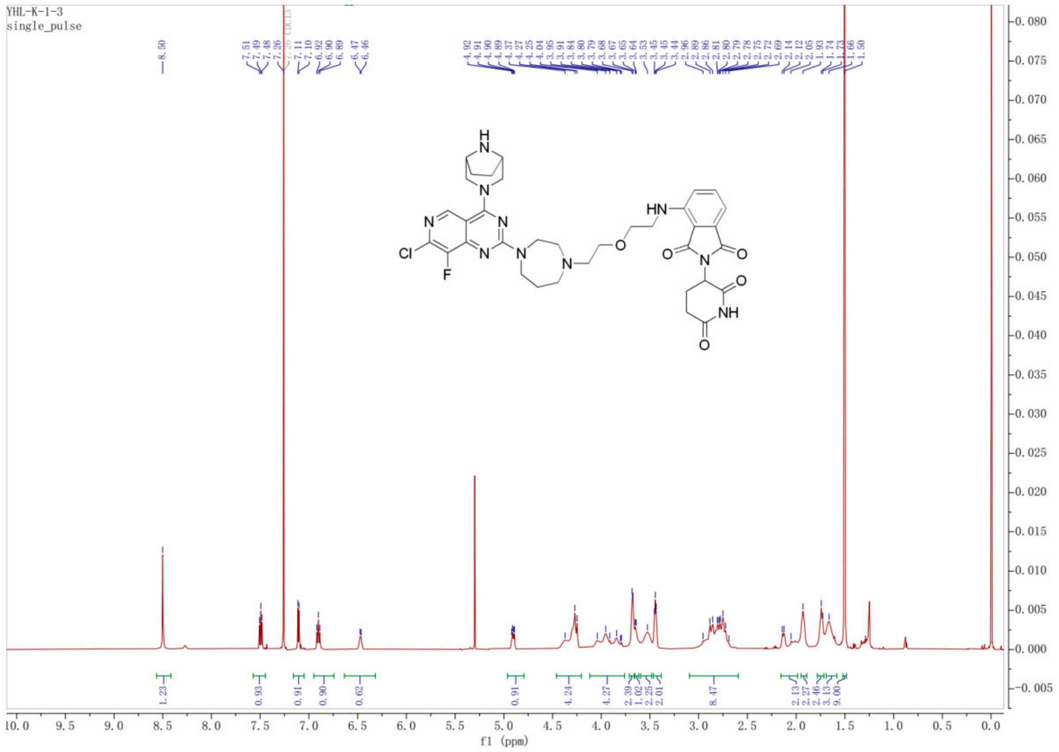

Figure S48. ^{1}H NMR spectrum of **23a**.

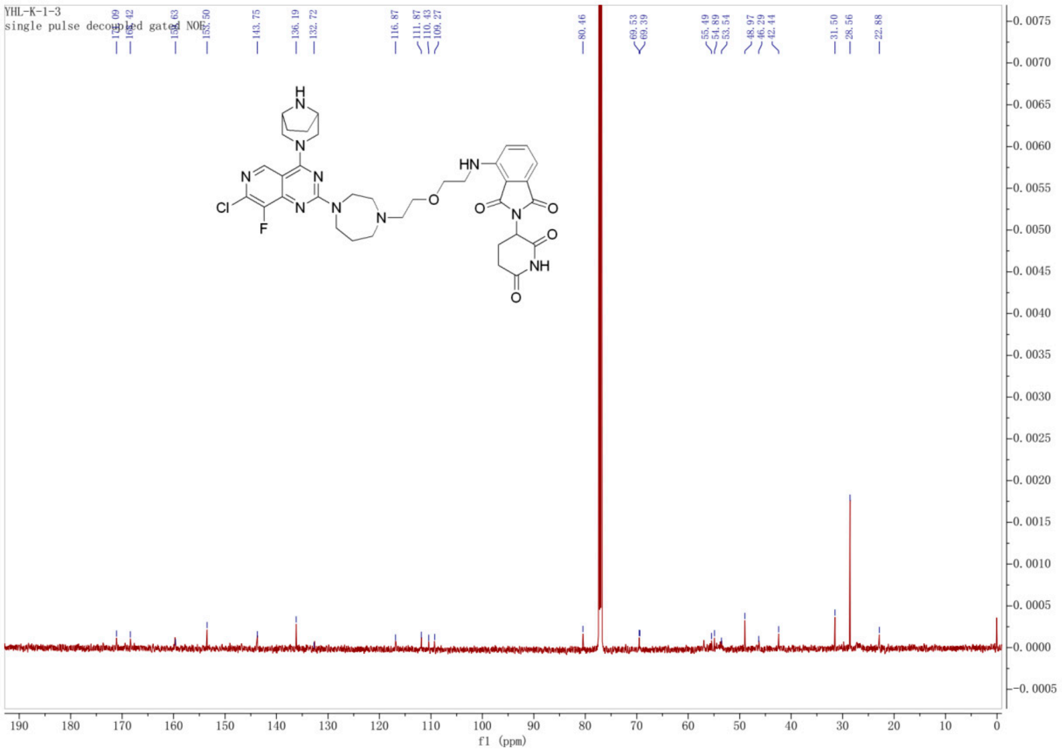

Figure S49. ^{13}C NMR spectrum of **23a**.

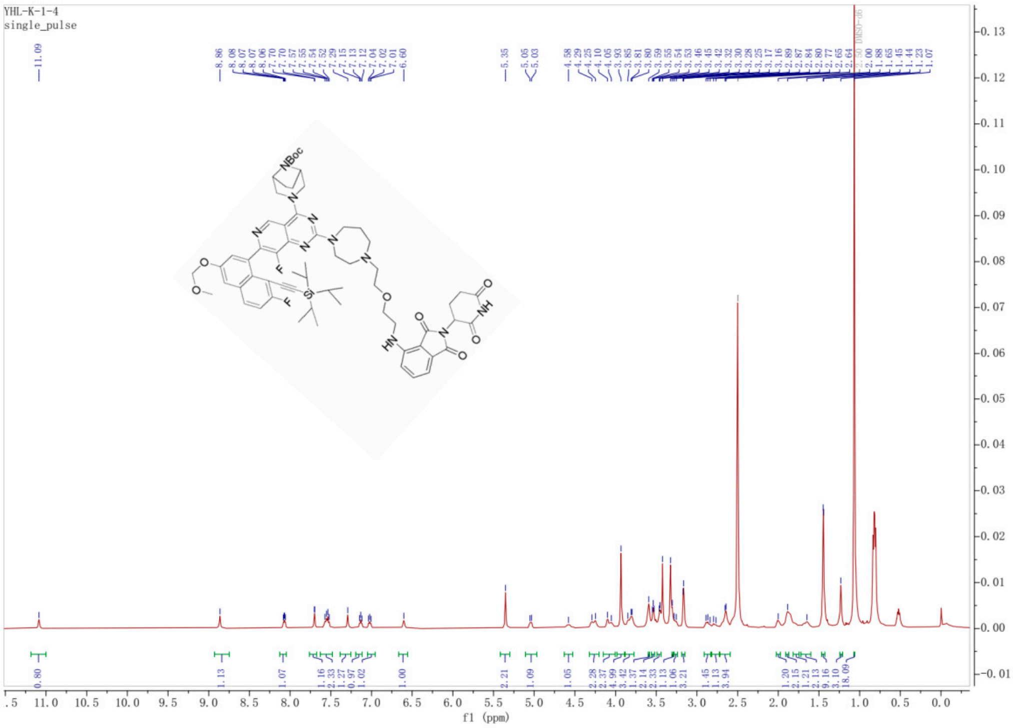

Figure S50. ^{1}H NMR spectrum of **24a**.

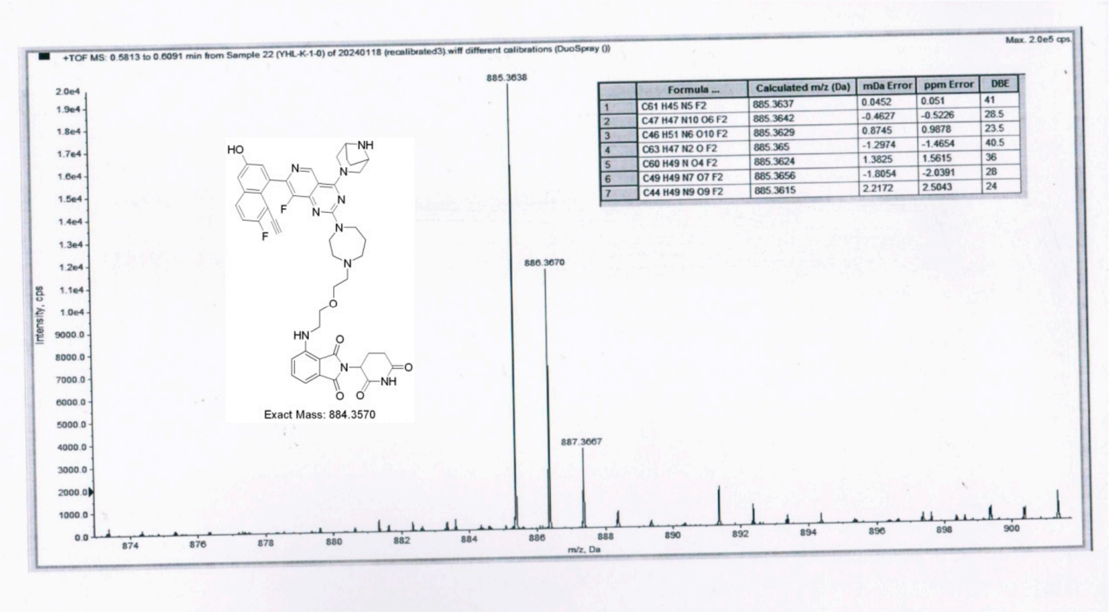

Figure S51. HR-MS chromatogram spectrum of **26a**.

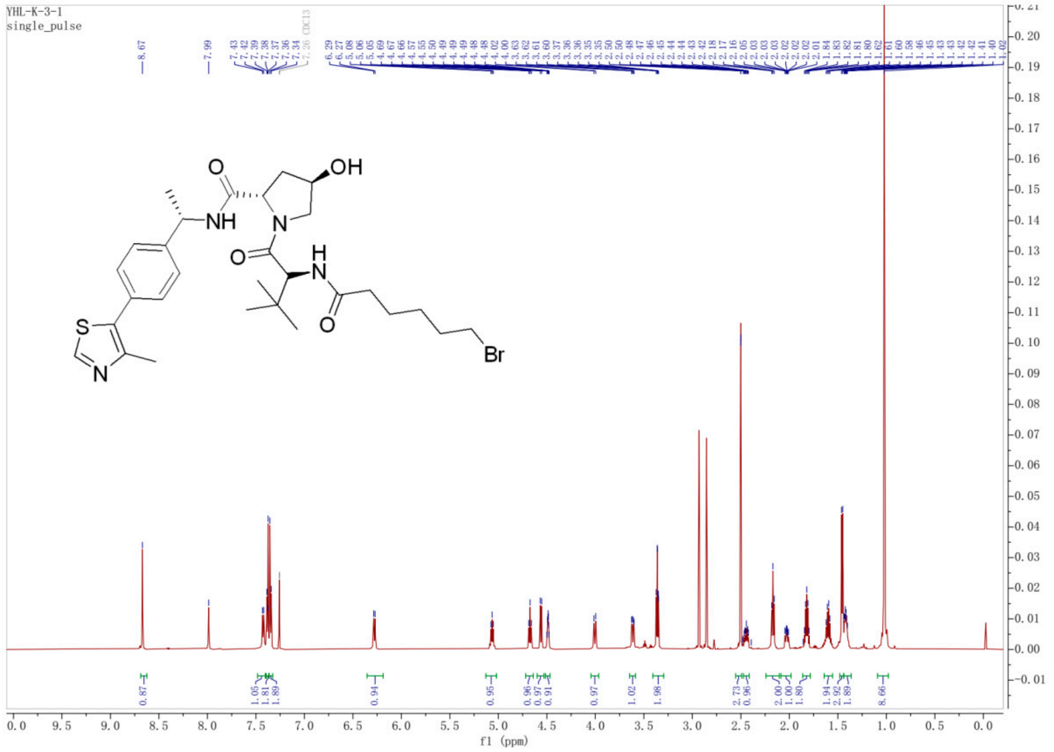

Figure S52. ^{1}H NMR spectrum of **31**.

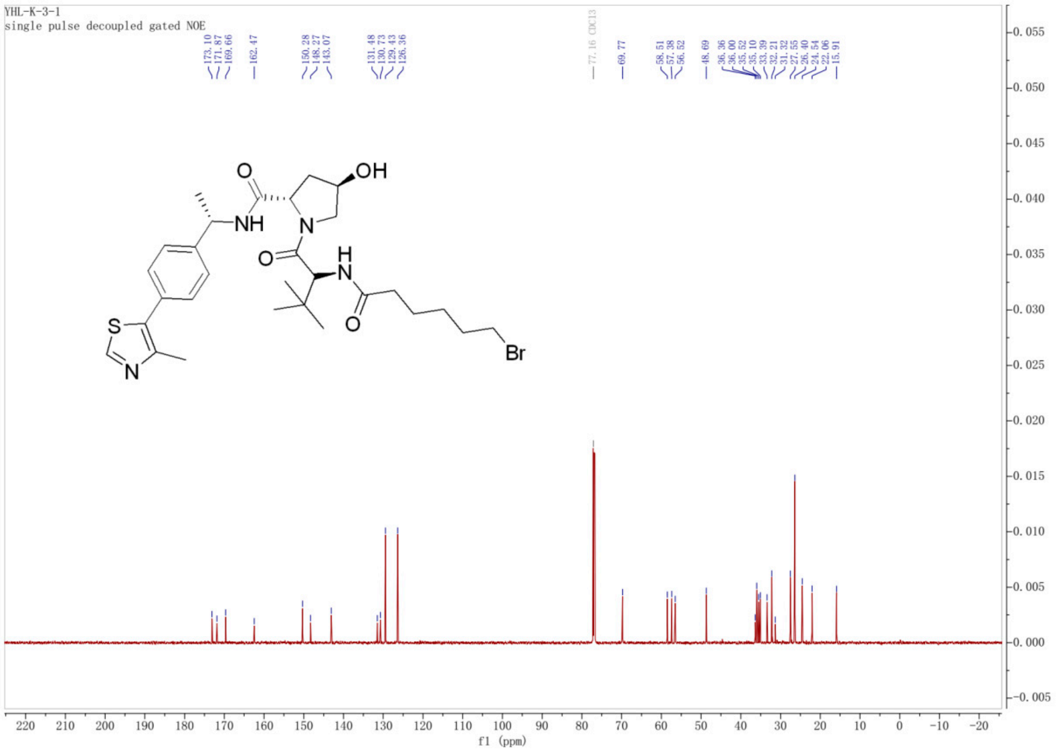

Figure S53. ^{13}C NMR spectrum of **31**.

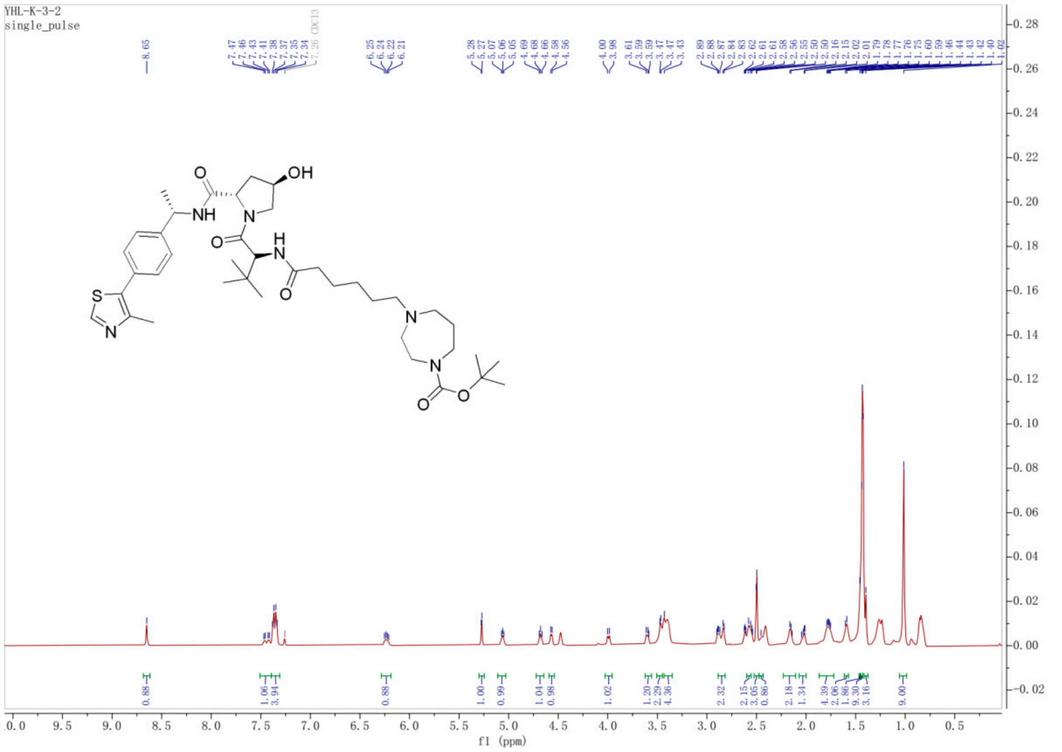

Figure S54. ^{1}H NMR spectrum of **32**.

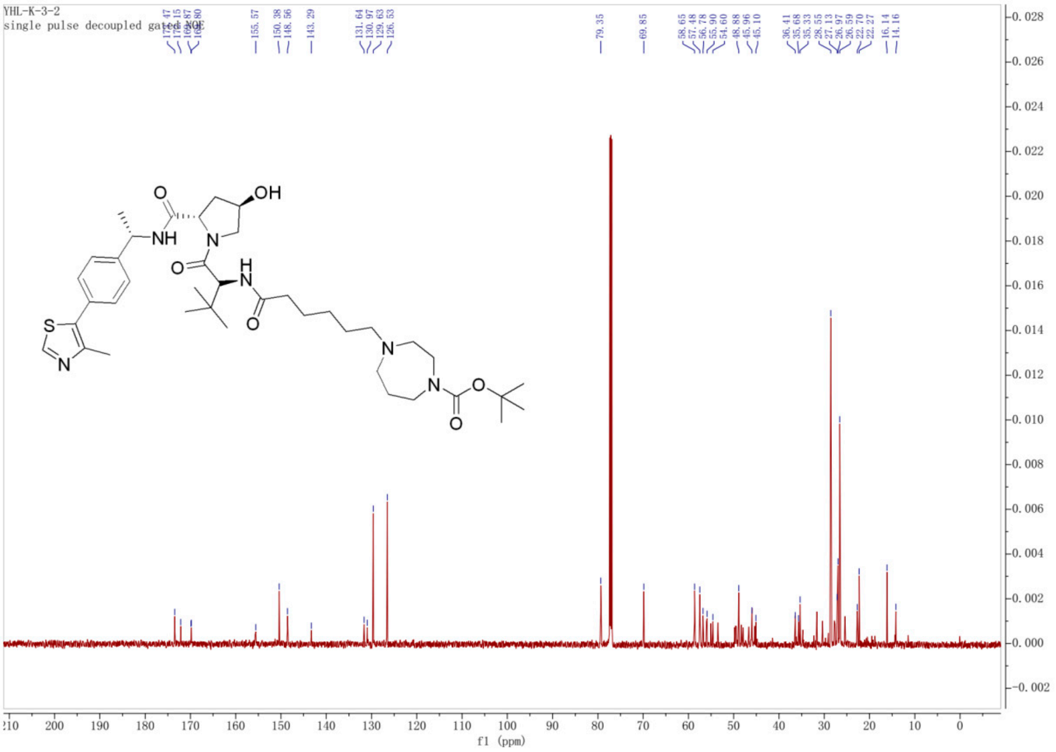

Figure S55. ^{13}C NMR spectrum of **32**.

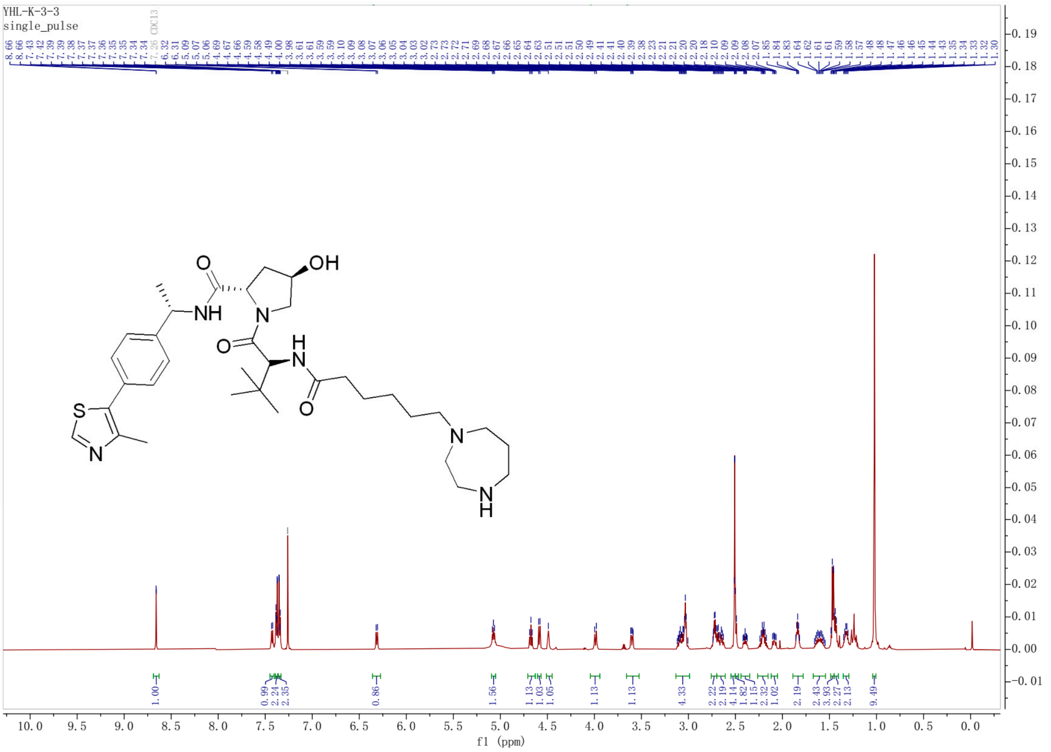

Figure S56. ^{1}H NMR spectrum of **33**.

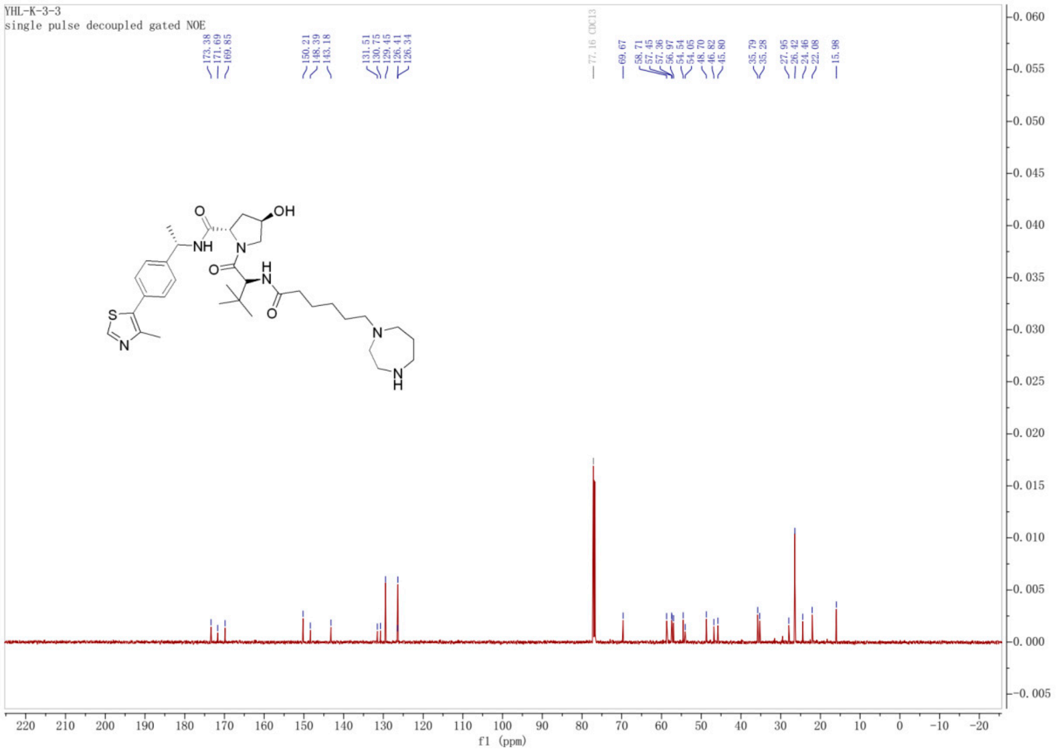

Figure S57. ^{13}C NMR spectrum of **33**.

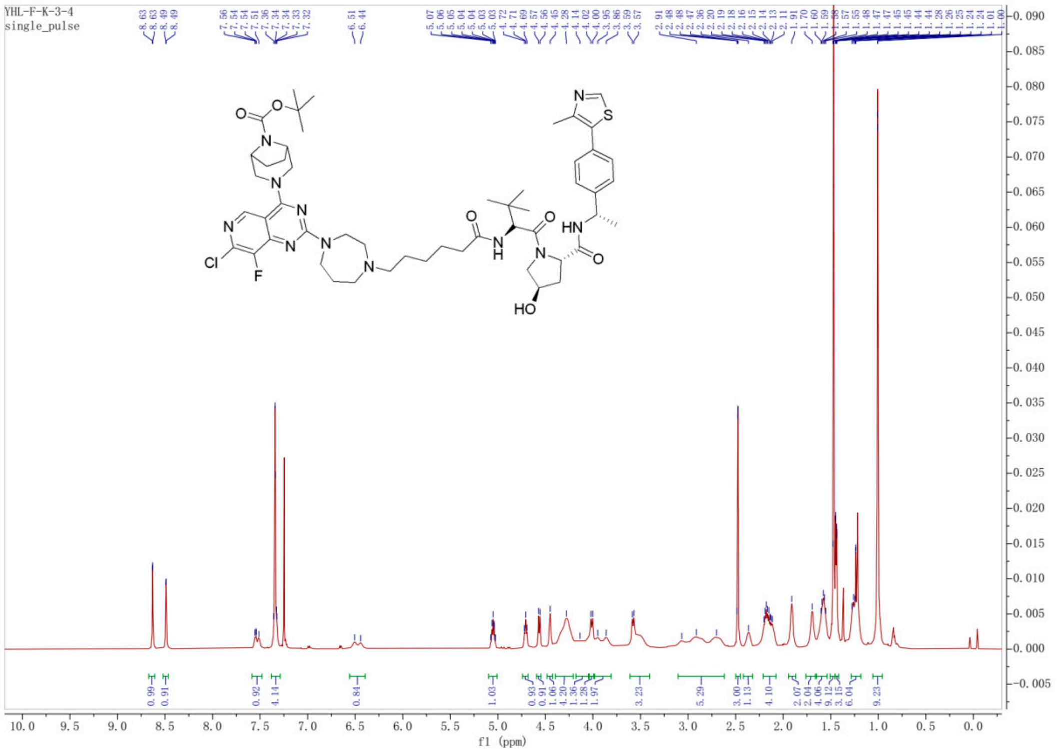

Figure S58. ^{1}H NMR spectrum of **23b**.

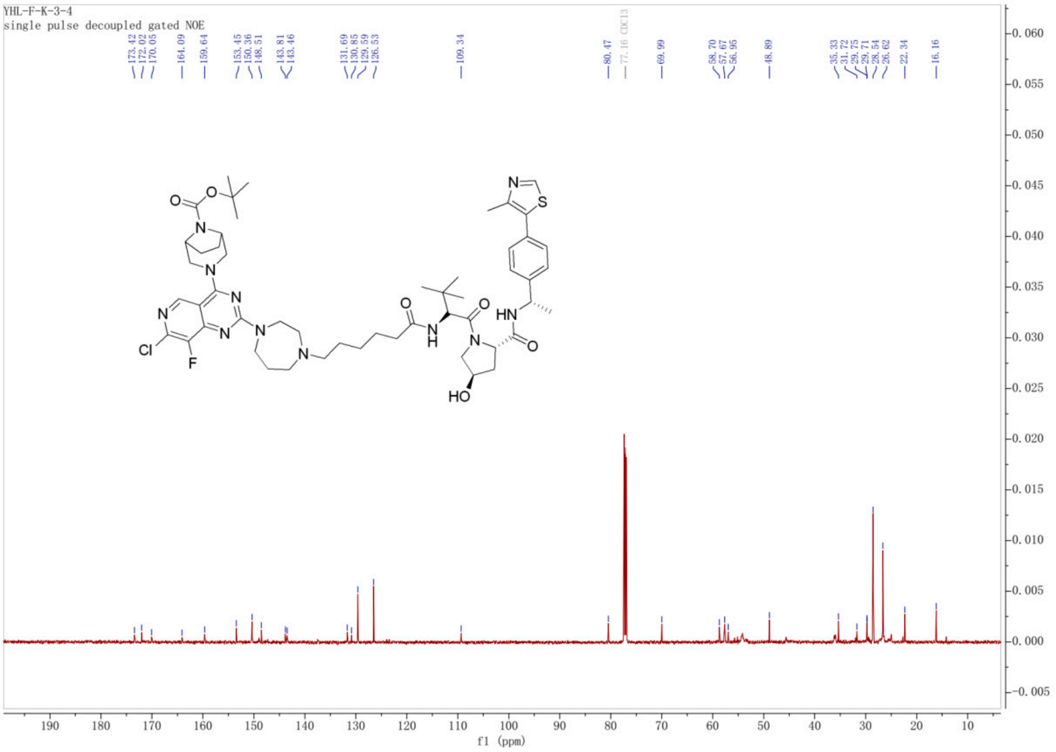

Figure S59. ^{13}C NMR spectrum of **23b**.

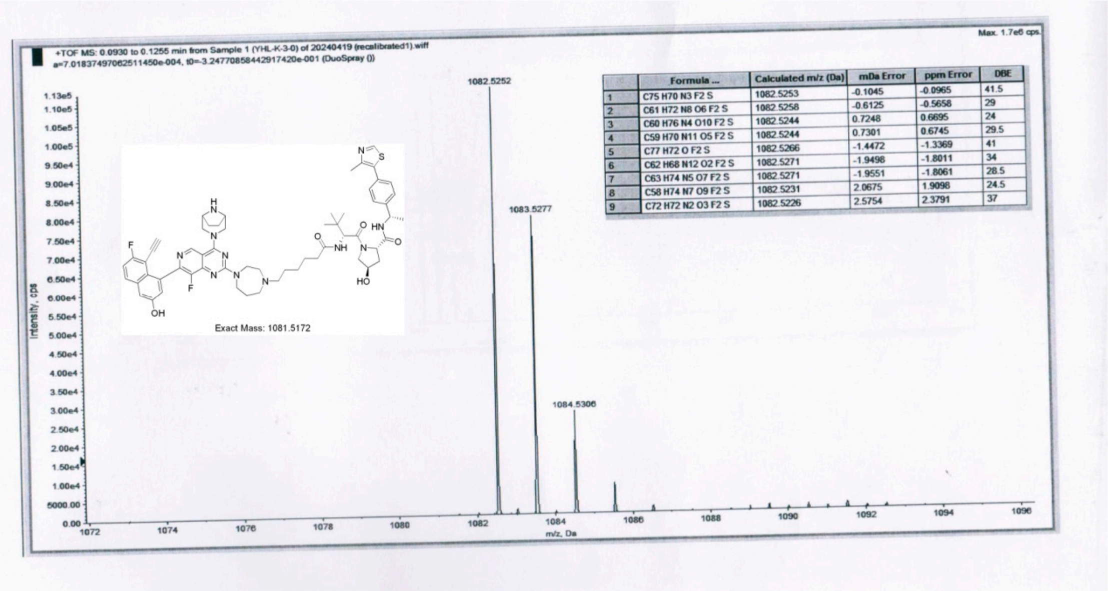

	Formula ...	Calculated m/z (Da)	mDa Error	ppm Error	DBE
1	C75 H70 N3 F2 S	1082.5253	-0.1045	-0.0965	41.5
2	C61 H72 N8 O6 F2 S	1082.5258	-0.6125	-0.5658	29
3	C60 H76 N4 O10 F2 S	1082.5244	0.7248	0.6695	24
4	C59 H70 N11 O5 F2 S	1082.5244	0.7301	0.6745	29.5
5	C77 H72 O F2 S	1082.5266	-1.4472	-1.3369	41
6	C62 H68 N12 O2 F2 S	1082.5271	-1.9498	-1.8011	34
7	C63 H74 N5 O7 F2 S	1082.5271	-1.9551	-1.8061	28.5
8	C58 H74 N7 O9 F2 S	1082.5231	2.0675	1.9098	24.5
9	C72 H72 N2 O3 F2 S	1082.5226	2.5754	2.3791	37

Figure S60. HR-MS chromatogram spectrum of **26b**.

Table S1 KRAS-G12D inhibitory activity of the inhibitors

ID	Structure	Curve	IC_{50} (nM)
**10a**	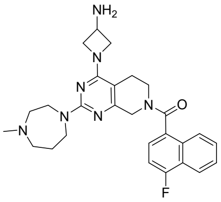	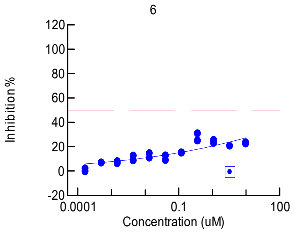	>1000
**10b**	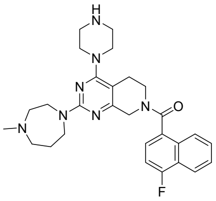	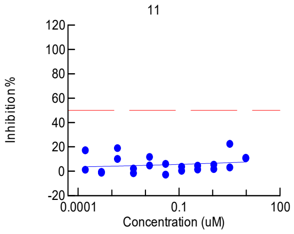	>1000
**10c**	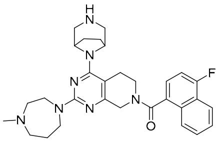	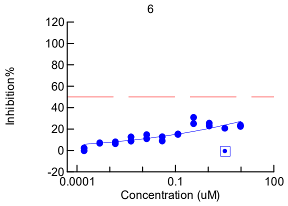	>1000
**10d**	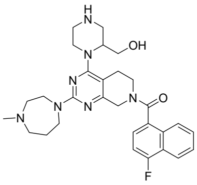	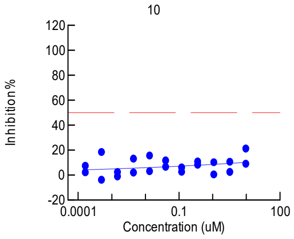	>1000

ID	Structure	Curve	IC50 (nM)
**10f**	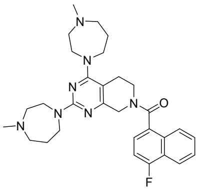	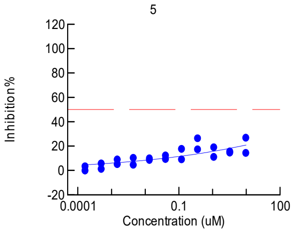	>1000
**10g**	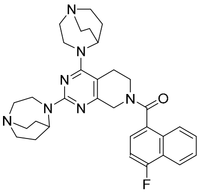	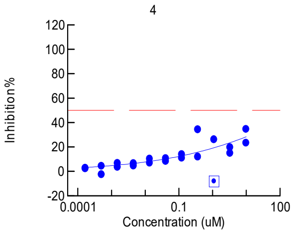	>1000
**10h**	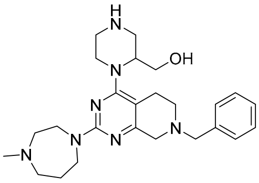	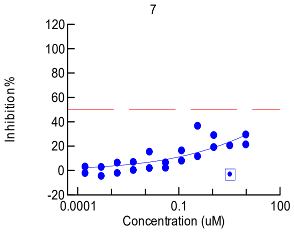	>1000
**10e**	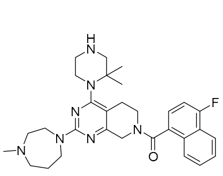	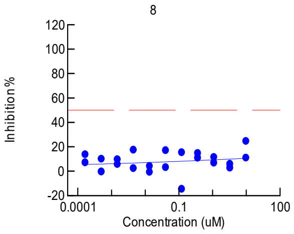	>1000

ID	Structure	Curve	IC_{50} (nM)
**10i**	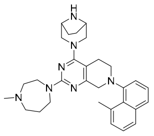	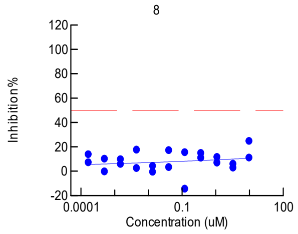	>1000
**10j**	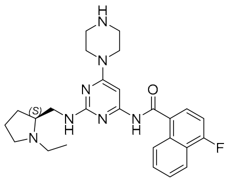	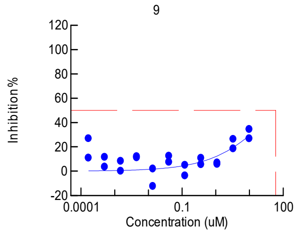	>1000
**10k**	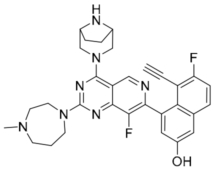	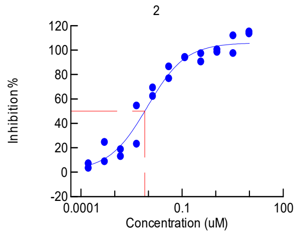	9.30
MRTX1133	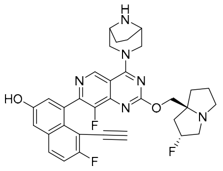	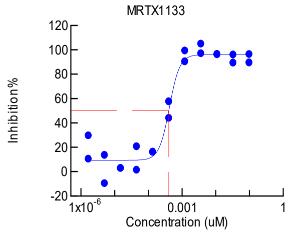	0.42
